# Design, synthesis and biological evaluation of buthutin derivatives as cardioprotective agents

**DOI:** 10.1007/s13659-025-00497-9

**Published:** 2025-02-05

**Authors:** Yuan Liu, Fa-Qi Wang, Xin-Hao Hua, Shu-Han Yang, Li-Ning Wang, Yun-Sheng Xu, Chen-Yue Shao, Xiang-Bo Gou, Yu-Ming Liu

**Affiliations:** 1 Department of Pharmacy Engineering, Tianjin University of Technology, Tianjin, 300384 People’s Republic of China; 2 College of Traditional Chinese Medicine, Tianjin Univerisity of Traditional Chinese Medicine, Tianjin, 300193 People’s Republic of China

**Keywords:** *Buthus martensii*, Amide-guanidine derivatives, Cardioprotective agents, NHE-1

## Abstract

Natural products are the important sources in cardiovascular drug development. In this study, twenty-nine buthutin derivatives were designed, synthesized, and evaluated for their NHE-1 inhibition and protective effects on cardiomyocyte injury. The structure of the newly synthesized compounds had been confirmed by ^1^H-NMR, ^13^C-NMR, and HR-ESI-MS spectra. Among all target compounds at 1 μM, compounds **9d**, **9f**, **9k**, **9m**, and** 9n**, with a protection ratio exceeding 30%, exerted stronger protective effects on H9c2 cardiomyocyte than positive control dexrazoxane and buthutin A. Meanwhile, compounds **9k**, **9m**, and **9o** showed the significant NHE-1 inhibitory activities on H9c2 cardiomyocyte, all with a dpHi/min value less than 0.23. What is more, compounds **9k**, **9m**, **9o** and buthutin A all exhibited the specificity on NHE-1 inhibition. Molecular modelling studies suggested the ability of compounds **9m** and **9o** to establish interactions with three hydrogen bonds to Asp267 and Glu346 of NHE-1, but also the ability with much lower CDOCKER energies than positive control cariporide and buthutin A. The structure–activity relationship (SAR) studies suggested that the presences of amide group, four-carbon linker, and *para* hydroxyl benzene ring were advantageous pharmacophores for above two pharmacological actions. This research would open new avenues for developing amide-guanidine-based cardioprotective agents.

**Graphical Abstract:**

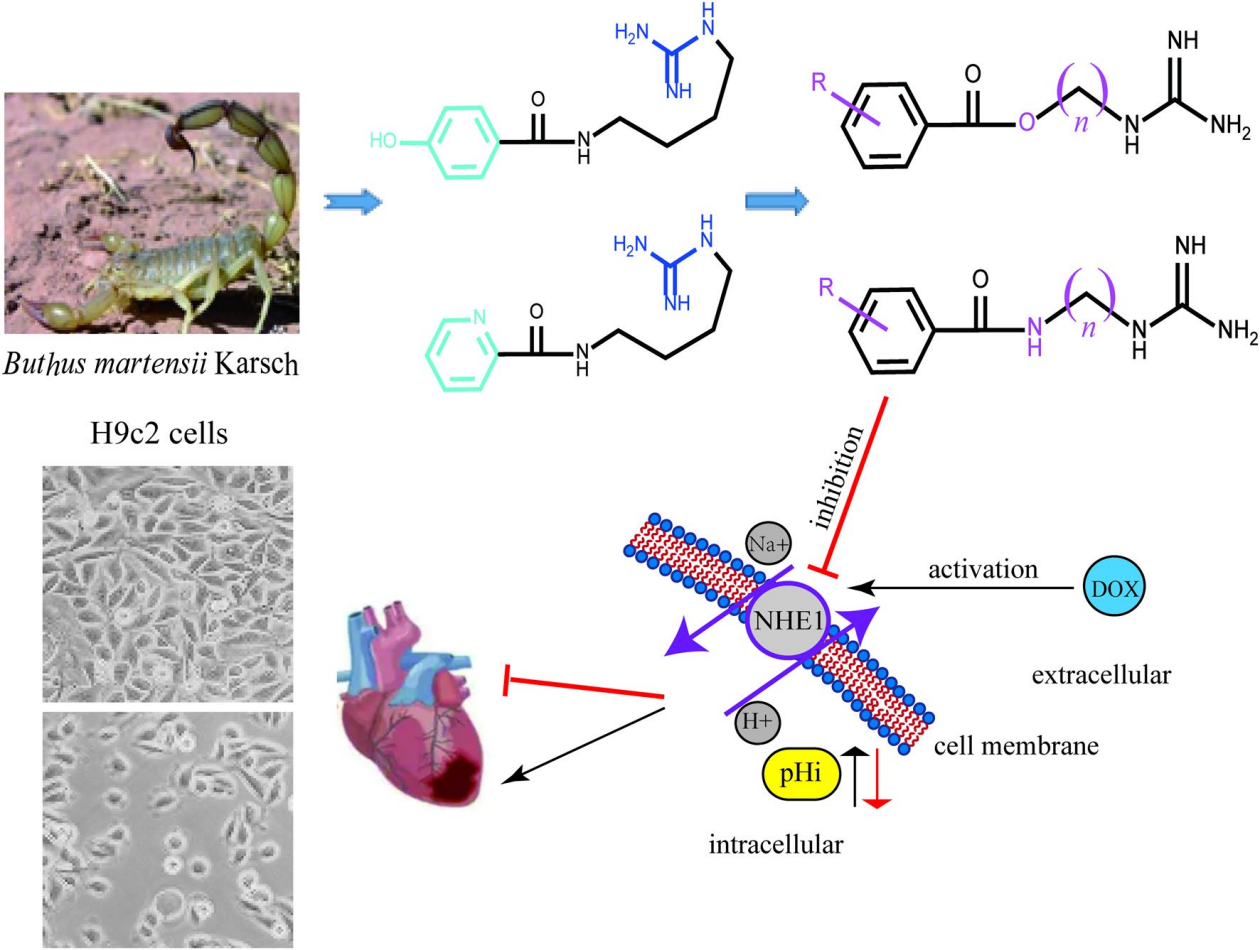

**Supplementary Information:**

The online version contains supplementary material available at 10.1007/s13659-025-00497-9.

## Introduction

The Na^+^ /H^+^ exchangers (NHEs) are a group of membrane proteins that transport one Na^+^ into the cells and one H^+^ out of the cells in order to regulate the intracellular pH and cell volume [1, 2]. There are about ten NHEs isoforms, and NHE-1 is the major isoform expressed in the heart, which plays a key role in the progression of cardiac hypertrophy to heart failure [3]. It was reported that the increased NHE-1 gene expression led to the increased NHE-1 activity, which ultimately was participated in the cardiac hypertrophy [4]. Besides, the over-expression of NHE-1 in H9c2 cardiomyocytes could also induce myocardial hypertrophy [5]. In contrast, the myocardial NHE-1 expression knockdown by the specific siRNA could significantly alleviate the myocardial hypertrophy of rats [6].

As known, doxorubicin (Dox) is a broad-spectrum anthracycline drug widely used in the treatment of several solid tumors (such as breast cancer, ovarian cancer and gastrointestinal malignancies) and hematological malignancies (such as lymphoma and leukemia) in adults and children [7]. However, Dox can also lead to severe cardiotoxicity, including irreversible degenerative cardiomyopathy and heart failure [8]. Some researches reported that Dox could cause cardiotoxicity because of triggering free radical release, calcium overload, increased NHE-1 activity, apoptosis and so on [9–11]. At present, dexrazoxane (Dex) was the only drug approved by Food and Drug Administration (FDA) to alleviate the cardiotoxicity caused by Dox [12]. Notably, selective inhibition of NHE-1 could also alleviate the Dox-induced cardiotoxicity in rats [13]. Considering Dex’s adverse effects such as the secondary malignancies induction [14], it is worthy of further developing innovative drugs for Dox-induced cardiotoxicity considering the role of NHE-1 inhibition.

**Fig. 1:**
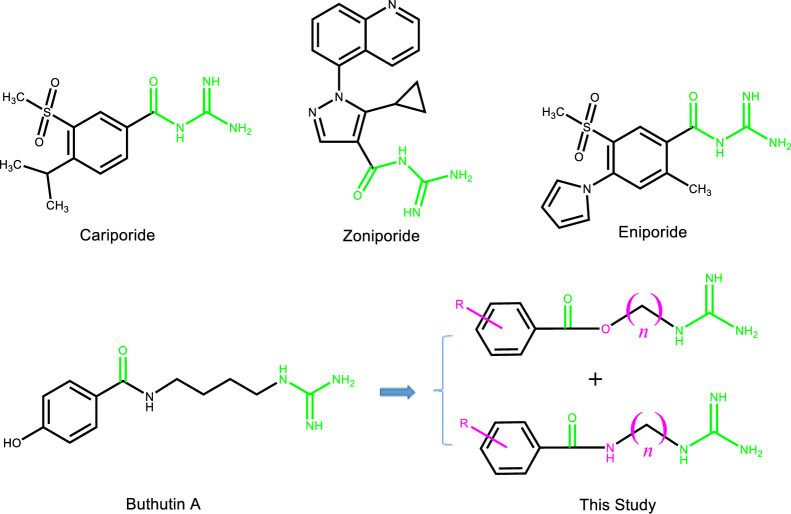
Reference NHE-1 inhibitors and design of target compounds

Now the structural types of selective NHE-1 inhibitors are mainly acylguanidine, and the animal experiments have shown that they have a good protective effect on myocardial ischemia and reperfusion injury, and can reduce the mortality of myocardial infarction, infarction size and arrhythmia occurrence [15]. It was reported that cariporide, eniporide and zoniporide (Fig. 1) had entered the clinical stage, but the clinical investigations of cariporide and eniporide didn’t obtain the expected results [16]. Then it is very meaningful to search for the NHE-1 inhibitors with novel structural types.

Natural products are the important sources in cardiovascular drug development [17–20]. In the process of our successively developing cardioprotective candidates [21, 22], we found that buthutin A (Fig. 1), previously isolated from *Buthus martensii* by our team [23], had the protective effect on cardiomyocyte injury caused by Dox and the inhibitory activity on NHE-1 (Table 1). In view of its rare amide-guanidine skeleton, we modified the chemical structure of buthutin A and synthesized twenty-nine compounds. The structural types of above amide-guanidine derivatives were obviously different from that of acylguanidine references, because we embedded different lengths of carbon chains and amino functional groups (or ether bonds) in the middle by keeping apart the acyl group from the guanidine group (Fig. 1). We further examined the inhibitory effects of all synthesized compounds on the NHE-1 activity and protective effects on the H9c2 cardiomyocyte injury induced by Dox. The results showed that some compounds revealed significant protective effects on the H9c2 cardiomyocyte injury induced by Dox, and that they also exerted significant inhibition on NHE-1, which would open new avenues for developing amide-guanidine-based cardioprotective agents.

**Table 1:** The effects of all compounds at 1 μM against H9c2 cardiomyocyte injury and NHE-1 activity 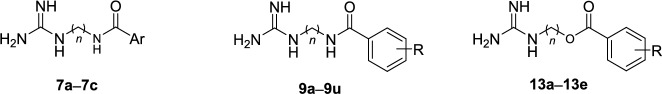

Compounds	n	R		Protection rate (%)	dpHi/min
**7a**	4		2-py	–	–
**7b**	4		3-py	20.87 ± 0.063	0.55 ± 0.035^&^
**7c**	4		4-py	–	–
**9a**	3	2-OH		6.56 ± 0.055	–
**9b**	3	3-OH		11.02 ± 0.15	–
**9c**	3	4-OH		29.40 ± 0.033*	1.00 ± 0.11^&^
**9d**	3	3-OCH_3_,4-OH		32.52 ± 0.041*	0.94 ± 0.16^&^
**9e**	3	2,4-OH		17.24 ± 0.093	0.73 ± 0.066^&^
**9f**	3	3,4-OH		40.05 ± 0.060**	0.64 ± 0.19^&^
**9g**	3	3,5-OH		18.26 ± 0.031	0.65 ± 0.045^&^
**9h**	3	3,4,5-OH		–	–
**9i**	4	2-OH		9.76 ± 0.012	0.67 ± 0.16^&^
**9j**	4	3-OH		27.12 ± 0.079	0.73 ± 0.051^&^
**9k**	4	3-OCH_3_,4-OH		33.14 ± 0.028***	0.15 ± 0.099^&&#^
**9l**	4	2,4-OH		19.90 ± 0.051	0.55 ± 0.16^&&^
**9m**	4	3,4-OH		31.50 ± 0.037**	0.23 ± 0.064^&&#^
**9n**	4	3,5-OH		37.80 ± 0.026***	0.51 ± 0.069^&^
**9o**	4	3,4,5-OH		22.02 ± 0.030	0.18 ± 0.083^&&#^
**9p**	5	2-OH		–	–
**9q**	5	3-OH		–	–
**9r**	5	4-OH		–	–
**9s**	5	3-OCH_3_,4-OH		–	–
**9t**	5	3,5-OH		–	–
**9u**	5	3,4,5-OH		15.67 ± 0.17	–
**13a**	3	3-OH		8.67 ± 0.11	–
**13b**	3	4-OH		–	–
**13c**	3	3-OCH_3_,4-OH		–	–
**13d**	3	3,5-OH		–	–
**13e**	4	4-OH		–	–
Buthutin A				30.67 ± 0.014***	0.054 ± 0.011^&&&##^
Dexrazoxane				22.31 ± 0.15	
Cariporide					0.56 ± 0.08^&&^

The results of the protection rate and dpHi/min value were expressed as mean ± SEM

i) For the protection rate measurement, the value in Dex group set as positive control group. ^*^p < 0.05, ^**^p < 0.01, and ^***^p < 0.001 *vs.* Dex group

ii) For the NHE1 activity, the dpHi/min value in cariporide group set as positive control group. The dpHi/min value of the non-drug treated group as model group was 1.18 ± 0.011. ^&^*p* < 0.05, ^&&^*p* < 0.01, and ^&&&^*p* < 0.001 *vs.* model group; ^#^*p* < 0.05, ^##^*p* < 0.01, and ^###^*p* < 0.001 *vs.* cariporide group

iii) “–” represented no effect on the H9c2 cardiomyocyte injury or NHE-1 activity

## Results and discussion

### Design and synthesis of buthutin derivatives

**Fig. 2:**
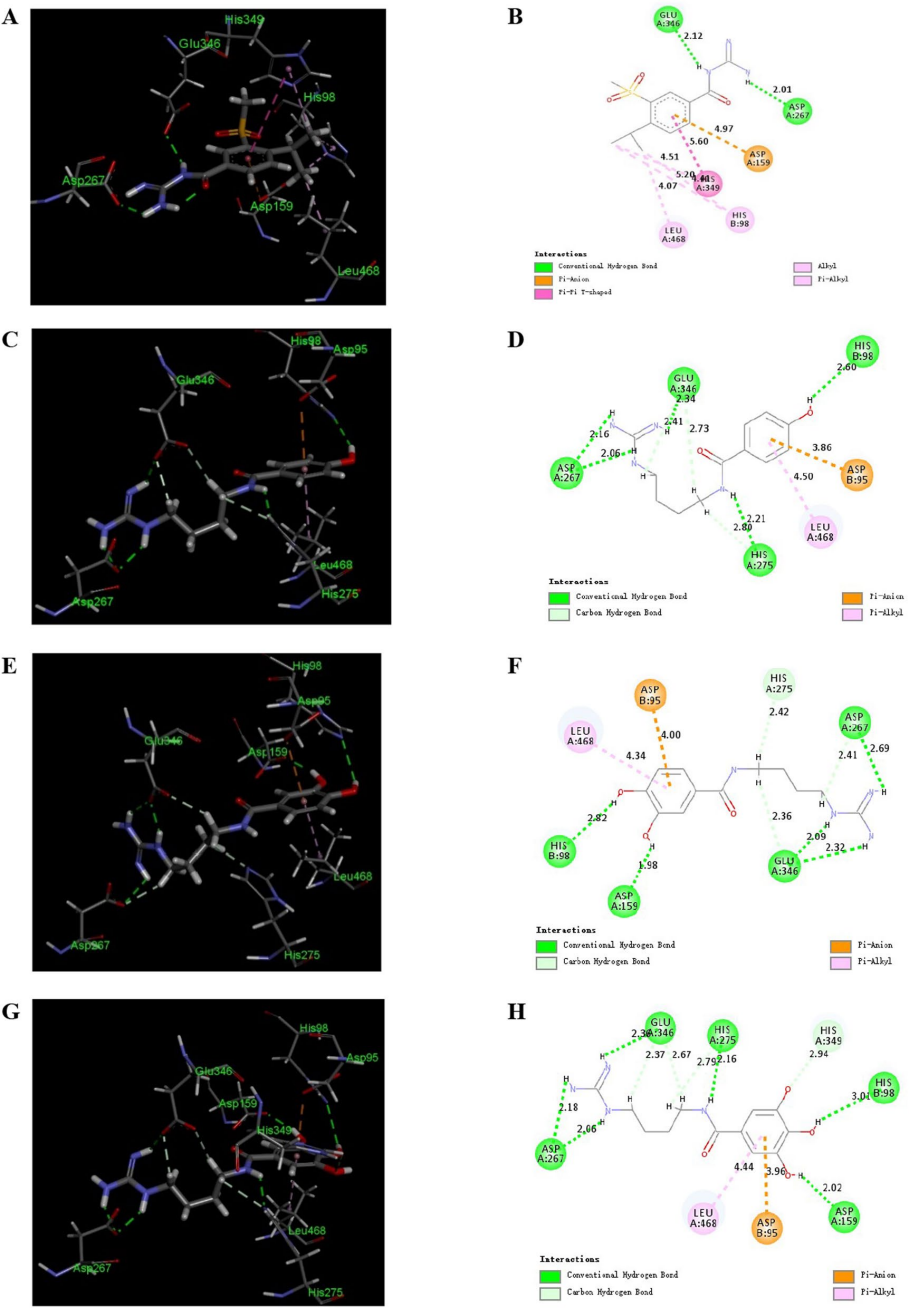
Binding models of cariporide, buthutin A, compounds **9m** and **9o** with NHE-1 (PDB: 7DSX). **A** 3D binding mode of cariporide with NHE-1; **B** 2D binding mode of cariporide with NHE-1; **C** 3D binding mode of buthutin A with NHE-1; **D** 2D binding mode of buthutin A with NHE-1; **E** 3D binding mode of compound **9m** with NHE-1; **F** 2D binding mode of compound **9m** with NHE-1; **G** 3D binding mode of compound **9o** with NHE-1; **H** 2D binding mode of compound **9o** with NHE-1

Inspired by the evident inhibition of buthutin A on NHE-1, we conducted the molecular docking of buthutin A with NHE-1 by comparison with cariporide. Although being similar binding mode to cariporide, buthutin A with three hydrogen bonds to Asp267 and Glu346, had more binding sites than cariporide (with two hydrogen bonds to Asp267 and Glu346), and caused much lower CDOCKER energy (−34.71 kcal/mol) with NHE-1 in comparison to that (−24.17 kcal/mol) of cariporide (Fig. 2). Cariporide is the best studied specific and selective NHE-1 inhibitor [24]. Interactions of the guanidine group of cariporide with Asp267 are believed to stabilize the complex in the outward-facing state, while Glu346 is a crucial residue involved in interactions with cariporide [24]. Now it was evidenced that the hot-spot NHE-1 residues Asp267 and Glu346 coordinate the Na^+^ ion in the outward-facing state during ion exchange, because the guanidine group of cariporide is of a similar size and charge with a (partially) hydrated Na^+^ ion [25]. Accordingly, these modeling studies provided crucial insights for guiding further drug design using buthutin A as lead compound. As shown in Fig. 1, buthutin A can be divided into four regions, i.e., guanidine group, phenyl ring, amide linkage and four-carbon linker, respectively. The guanidine group was kept unchanged, while modifications were focused on the other three parts (Fig. 1). Firstly, introducing diverse substituent groups to phenyl ring or replacement of phenyl ring with pyridine ring prompted the syntheses of compounds **7a**–**7c** and **9i**–**9o**. Additionally, the lengths of carbon chains were varied to synthesize compounds **9a**–**9h** and **9p**–**9u**. Subsequently, only replacement of amide linkage with ester linkage or concurrently adjusting the carbon linker lengths provided compounds **13a**–**13e**.

**Scheme 1:**
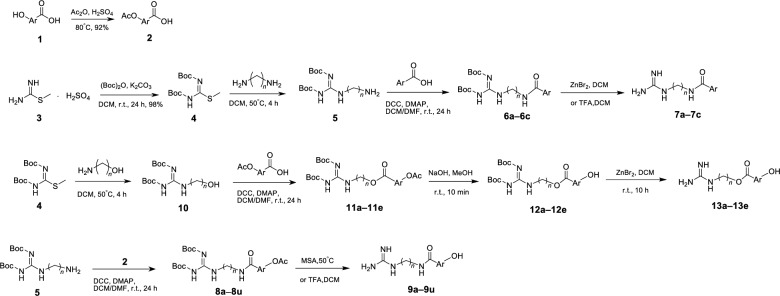
Synthesis of target compounds **7a**–**7c** and **9a**–**9u** and **13a**–**13e**

Synthesis of the target compounds was outlined in Scheme 1. In this study, methyl isothioureide sulfate was used as the starting material, and its amino group was protected by Boc group to obtain compound **4**. Then compound **4** was reacted with 1, 3-propanediamine, 1, 4-butanediamine and 1, 5-pentylenediamine (or 3-amino-1-propanol and 4-amino-1-butanol) under certain conditions to give series intermediates **5** (or **10**). The hydroxybenzoic acids **1** were protected with the acetyl group beforehand to give acetoxybenzoic acid intermediates **2**. Series compounds **5** were subjected to amidation with pyridinic acid and acetoxybenzoic acid intermediates **2** to give compounds **6a**–**6c** and **8a**–**8u**, respectively. After removing the Boc protecting group of compounds **6a**–**6c**, target compounds **7a**–**7c** were obtained. Two kinds of protection groups were removed from compounds **8a**–**8u** with methanesulfonic acid (MSA) or trifluoroacetic acid (TFA) through one step to obtain target compounds **9a**–**9u**. Series compounds **10** were esterified with the acetoxybenzoic acid intermediates **2** to afford compounds **11a**–**11e**. The acetyl protecting groups of compounds **11a**–**11e** were first removed with NaOH solution to obtain the compounds **12a**–**12e**, and then their Boc protection groups were taken off using ZnBr_2_ to acquire target compounds **13a**–**13e**.

The structures of all target compounds were confirmed by ^1^H-NMR, ^13^C-NMR and HR-ESI–MS spectra. As a representative example, we analyze the spectrum of compound **9h** as follows. Its molecular formula was determined to be C_11_H_16_N_4_O_4_ based on a protonated molecule peak at *m/z* 269.1272 [M + H]^+^ (calcd 269.1250 for C_11_H_17_N_4_O_4_) in its HR-ESI–MS. In its ^1^H-NMR spectrum, the presence of symmetric benzene ring was confirmed by the single signal with two aromatic protons at δ_H_ 6.74. The methylene protons at δ_H_ 3.08 and δ_H_ 3.23 arose as low field values due to the adjacent nitrogen atoms, and the quintet signal at δ_H_ 1.71 showed the presence of a three-carbon aliphatic chain. In its ^13^C-NMR spectrum, four aromatic carbon resonances at δ_C_ 144.6, δ_C_ 136.2, δ_C_ 124.8, and δ_C_ 107.3 indicated one symmetric benzene ring. Characteristic signals at δ_C_ 171.2 and δ_C_ 158.2 confirmed the presence of an amide group and a guanidine group, respectively. The peaks of three-carbon linker chain were observed at δ_C_ 38.7, δ_C_ 36.9, and δ_C_ 27.6.

### Pharmacological evaluation

**Fig. 3:**
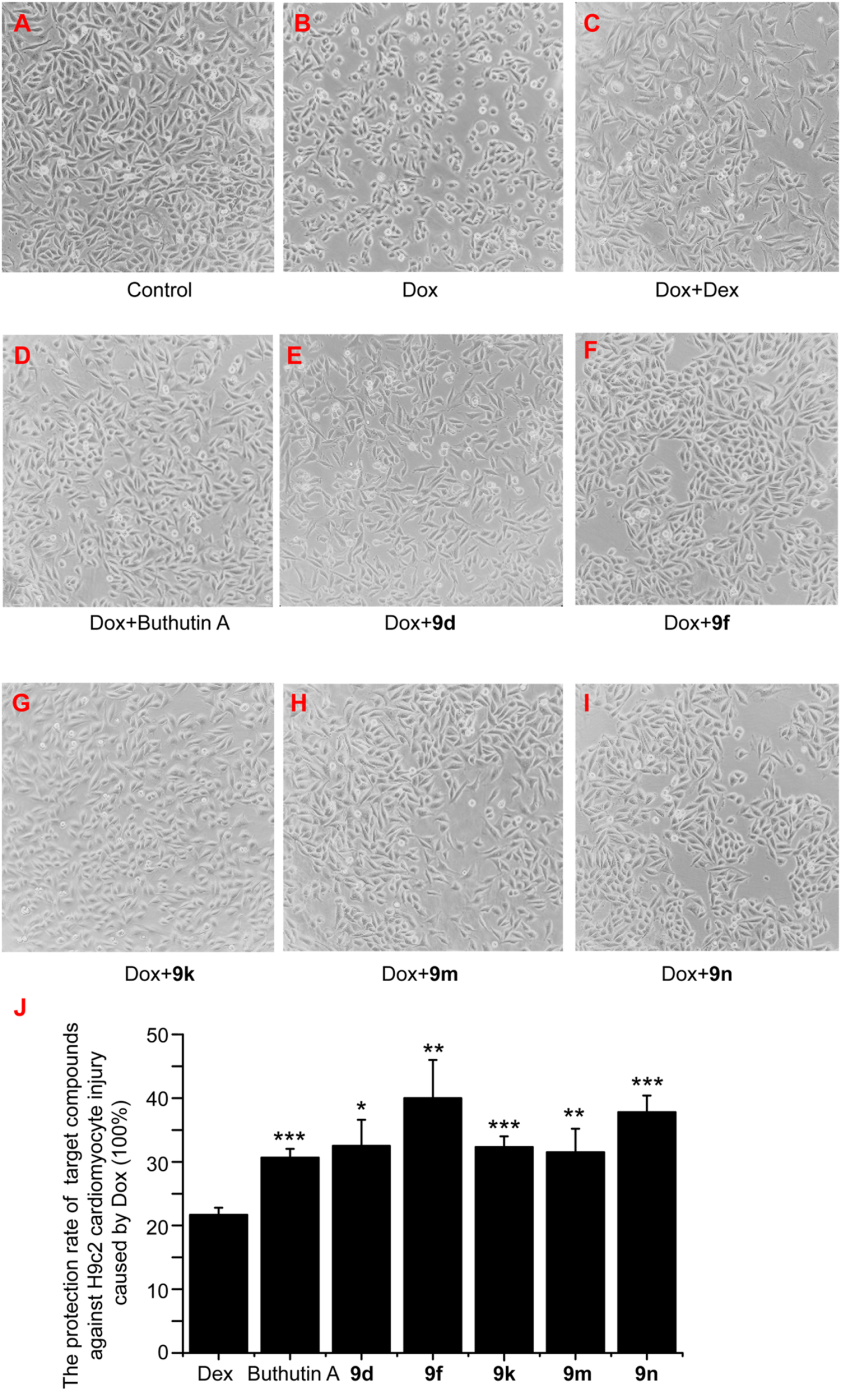
The effect of compounds **9d**, **9f**, **9k**, **9m**, **9n** and buthutin A on the morphology of H9c2 cardiomyocyte at the presence of Dox. **A** Control group; **B** Dox (1 μM) treated group for 24 h; **C** Dox (1 μM) and Dex (20 μM) treated group for 24 h; (D) Dox (1 μM) and buthutin A (1 μM) treated group for 24 h; **E** Dox (1 μM) and **9d** (1 μM) treated group for 24 h; **F** Dox (1 μM) and **9f** (1 μM) treated group for 24 h; **G** Dox (1 μM) and **9 k** (1 μM) treated group for 24 h; **H** Dox (1 μM) and **9m** (1 μM) treated group for 24 h; **I** Dox (1 μM) and **9n** (1 μM) treated group for 24 h; **J** the protection rate of Dex, buthutin A, **9d**, **9f**, **9k**, **9m** and **9n** on the injury caused by Dox. ^*^p < 0.05, ^**^p < 0.01, and ^***^p < 0.001 vs Dex group

In existence of Dox, we examined the cell survival rate of H9c2 cardiomyocyte under the treatment of target compounds and positive control Dex and buthutin A, with the results shown in Table 1. It can be seen that compounds **9d**, **9f**, **9k**, **9m**, and **9n** at 1 μM showed better protective effects against Dox-induced H9c2 cardiomyocyte injury than Dex and buthutin A, which exerted stronger protective effects with a protection ratio exceeding 30% (Table 1 and Fig. 3).

Taken as an ensemble, the general features of structure activity relationship (SAR) can be deduced from these data in Table 1: (1). By comparing the activities of compounds **7a–7c** with the corresponding compounds **9i**–**9j** and buthutin A, it was demonstrated that the aromatic derivatives with electron deficiency were not conducive to the protective effect against myocardial injury. (2). By comparing the activities of compounds **13a**–**13c** with the corresponding compounds **9b**–**9d**, it was shown that the protective effect of amide-guanidine derivatives was stronger than that of ester-guanidine derivatives, which was further verified by the comparison of the activities of compounds **13e** and buthutin A. (3). By comparing the activities of compounds **9p**–**9u** with the corresponding compounds **9i**–**9k** and** 9n**–**9o**, it was revealed that the derivatives with five-carbon linker were not conducive to the protective effect against myocardial injury. (4). By comparing the activities of compounds **9a**–**9h** with the corresponding compounds **9i**–**9o**, it was illustrated that most derivatives with three-carbon linker possessed a lower protective effect than those with four-carbon linker, except for compound **9f**, which had a higher protective effect than **9m**. (5). Different positions of hydroxyl groups on the benzene ring in amide-guanidine compounds caused regular variation on protective effects, with the rank order of *para*- > *meta*- > *ortho*-, such as **9c** > **9b** > **9a** or buthutin A > **9j** > **9i**. (6). In amide-guanidine compounds with a *p*-hydroxyl group, when another substituent was introduced into its *ortho* or *meta* position, respectively, the *meta* substitution derivatives had a stronger protective effect than those with *ortho* substitution, such as **9f**, **9d** > **9e** or **9k**, **9m** > **9l**. (7). when the hydroxyl groups of benzene ring were added to the number of three, the bioactivities of amide-guanidine compounds would decrease. For example, the protective effect of **9o** was weaker than all those of buthutin A, **9m**, and **9n**, which might be due to its too large spatial volume of aromatic ring in **9o**.

In order to investigate the protective mechanism of these compounds against myocardial injury, the inhibition on NHE-1 activity was measured with the dpHi/min of H9c2 cardiomyocyte under the treatment of target compounds and positive control cariporide (Table 1). Among all tested compounds at 1 μM, compounds **7b**, **9c, 9d, 9e**, **9f**, **9g**, **9i**, **9j**, **9k**, **9l**, **9m**, **9n**, and **9o** had the obvious inhibition effects on the NHE-1 activity. Furthermore, compounds **9k**, **9m**, and **9o** exerted superior inhibitions on the NHE-1 activity than that of cariporide. As shown in Table 1, the ester-guanidine derivatives **13a**–**13e** and five-carbon linker derivatives **9p**–**9u** had no obvious NHE-1 inhibitory activities. Three-carbon linker derivatives **9a**–**9h** showed weaker NHE-1 inhibitory activities than the corresponding four-carbon linker derivatives **9i**–**9o**, for example, **9g** versus **9n**. Four-carbon linker derivatives with *para* hydroxyl substitution in benzene ring revealed the strongest inhibitory activities on NHE-1, such as buthutin A > **9i**,**9j**. Meanwhile, In amide-guanidine compounds with a *p*-hydroxyl group, when another substituent was introduced into its *ortho* or *meta* position, respectively, the *meta* substitution derivatives exerted a stronger NHE-1 inhibition than those with *ortho* substitution, such as **9k**,**9m** > **9l**. All these results strongly supported the above SARs of myocardial injury protection.

To further evaluate the selectivity of buthutin A derivatives against other NHE isoforms, HEK293 cells expressing endogenous NHE-1, NHE-2 and NHE-3 [26, 27] were used to investigate the inhibitory effect on other NHEs activity. Reportedly, 3 µM cariporide was applied to inhibit most NHE-1 activity with minimal effect on other NHEs activities [28]. The results showed that the dpHi/min value were 1.33 ± 0.11 in model group and 0.44 ± 0.048 in 3 µM cariporide-treated group, which revealed that most NHE-1 activity was inhibited by 3 µM cariporide. However, additions of buthutin A, **9k**, **9m** and **9o** to 3 µM cariporide could not further enhance the inhibition of 3 µM cariporide on the NHEs activities, with the dpHi/min values 0.39 ± 0.028, 0.38 ± 0.044, 0.42 ± 0.023 and 0.37 ± 0.014, respectively. Therefore, buthutin A, **9k**, **9m** and **9o** could not obviously inhibit any other NHEs, mainly NHE-2 and NHE-3, which illustrated the specificity of inhibition on NHE-1 activity.

For clarifying the interaction mode of buthutin A derivatives in the active sites of NHE-1, molecular dockings were performed for compounds **9k**, **9m**, and **9o** using Discovery Studio 2017 R2 software. Amazingly, compounds **9m** and **9o** exhibited much lower CDOCKER energies of –36.05 kcal/mol and –43.53 kcal/mol with NHE-1, respectively, in comparison to that (–34.71 kcal/mol) of buthutin A. Compounds **9m** and **9o** showed very close binding mode to buthutin A, both with three hydrogen bonds to Asp267 and Glu346 (Fig. 2). However, their additional hydroxyl group was stabilized by hydrogen bond interactions with ASP159 or HIS349 (Fig. 2), which should avail to increase the inhibitory activity. Further exploration of their dose–response relationships on NHE-1 was worth investigating.

## Conclusions

In summary, twenty-nine buthutin derivatives (**7a–7b**, **9a–9u**, and **13a–13e**) were designed and synthesized, and their structures were well characterized by ^1^H-NMR, ^13^C-NMR, HR-ESI–MS analyses. These compounds were tested for their NHE-1 inhibitions and protective effects on cardiomyocyte injury for the first time. Among them, compounds **9d**, **9f**, **9k**,** 9m**, and **9n** displayed more potent protective effects against Dox-induced H9c2 cardiomyocyte injury than Dex and buthutin A, and compounds **9k**, **9m**, and **9o** exerted notable inhibitions on the NHE-1 activity surpassing that of positive control cariporide. What is more, compounds **9k**, **9m**, **9o** and buthutin A all exhibited the specificity on NHE-1 inhibition. Molecular modelling studies suggested the ability of compounds **9m** and **9o** to establish interactions with three hydrogen bonds to Asp267 and Glu346 of NHE-1, but also the ability with much lower CDOCKER energies than buthutin A and cariporide. Further, it could be concluded that all compounds had a similar structure–activity relationship between the inhibitory activities on NHE-1 and the protective effects against myocardial injury. The presences of amide group, four-carbon linker, and *para* hydroxyl benzene ring have been identified as pivotal pharmacophore for above two observed pharmacological actions, while both 3-methoxy-4-hydroxy-phenyl ring and 3,4-dihydroxy-phenyl ring also are conducive to the aforementioned activity. However, it could be inferred that the mechanism of myocardial protection was not entirely caused by NHE-1 inhibition, and that other mechanisms should also be involved, since some compounds (**9a**, **9b**, **9u**, **13a**) had cardioprotective effects but no NHE-1 inhibitory activities. To sum up, based on the promising results observed in both cell-based assessments, these derivatives merit further investigation in the pursuit of developing cardioprotective agents.

## Experimental section

### General

A Waters Acquity UPLC Class I/Xevo G2 QTOF mass spectrometer (Milford, MA, USA) was used to obtain the high-resolution ESI–MS (HR-ESI–MS) data. ^1^H-NMR and ^13^C-NMR spectra were acquired on a Bruker Avance III 400 spectrometer spectrometer (Karlsruhe, Germany) with TMS as an internal standard. Melting points were determined using an X-4 digital micro-melting tester (Beijing Teck Instrument Co., Ltd.). Chromatographic analysis for purity determination (HPLC) was achieved on a Ultimate®XB-C18; 4.6 × 250 mm; 5 μm; column (Welch Materials, Inc. Shanghai, China). Gradient elution was performed starting at 5% MeOH increasing to 100% MeOH at 20 min, and detection was conducted at 254 nm. The ratio of peak areas in the chromatograms was used to express the purity in percentage.

### Synthetic procedures

#### General procedure for the synthesis of intermediates *2*

To a 100 mL flask, hydroxybenzoic acids **1** (0.1 mmol) and acetic anhydride (10 mL) were added. While the mixture was stirred, concentrated sulfuric acid (0.15 mL) was drip in. The reaction mixture was refluxed at 80 °C for 2 h. By adding 60 mL distilled water, the white precipitate was formed, and then was filtered and dried to obtain acetoxybenzoic acid intermediate **2**.

#### General procedure for the synthesis of intermediates *4*

Methyl isothioureide sulfate (**3**, 0.025 mmol), 30% K_2_CO_3_ solution (12.5 mL), and di-*tert*-butyl dicarbonate (0.1 mol) were dissolved in CH_2_Cl_2_ (25 mL) and stirred for 24 h at room temperature. The crude product was washed with water, and was purified by silica gel column chromatography using petroleum ether/ethyl acetate (4:1, v/v) as eluent to give compound **4** (Yield 98%) as an off- white solid.

#### General procedure for the synthesis of intermediates *5* and* 10*

A solution of **4** (7.5 mmo1) in CH_2_Cl_2_ (20 mL) was added in batches to a alkyl diamine (1, 3-propylene diamine, or 1, 4-butyric diamine, or 1, 5-pentylenediamine) (0.03 mol). The reaction mixture was stirred at 50 °C for 4 h. The crude product was washed with water to give series compounds **5**. Series compounds **10** was prepared following the method described for the preparation of series compounds **5**, employing 3-amino-propanol or 4-amino-1-butanol instead of alkyl diamine.

#### General procedure for the synthesis of intermediates 6a–6c, 8a–8u, and 11a–11e

Intermediates **2** (0.04 mol, 1.0 eq) and **5** (1.0 eq) were dissolved in DCM (20 mL) or DMF (20 mL) solution. To this solution, DCC (1.2 eq) and DMAP (0.1 eq) were added. The resulting reaction mixture was stirred for 24 h at room temperature. The reaction progress was detected by TLC. After completion of the reaction, a large amount of by-product DCU would be precipitated in the solution and filtered. Excess solvent of filtrate was removed by evaporation under reduced pressure to obtain the corresponding amide products **8a**–**8u**. The method described above was used to synthesize amide products **11a**–**11e**, employing series compounds **10** instead of series compounds **5**. Similarly, amide products **6a**–**6c** were also synthesized using series compounds** 5** with 2-picolinic acid, nicotinic acid, isonicotinic acid, respectively.

#### Procedure for the synthesis of *7a*–*7c*

A mixture of compounds **6a**–**6c** (0.41 mmol, 1.0 eq) and zinc bromide (2.1 mmol, 5.0 eq) was stirred in DCM at room temperature for 10 h. The reaction progress was detected by TLC. After the completion of the reaction, the organic phase was washed with water, and further purified over Sephadex LH-20 eluted with MeOH to obtain target compounds **7a**–**7c**.

*N*-(4-guanidinobutyl)-2-pyridinecarboxamide (**7a**). Yield 72%, white solid. Mp: 171–173 °C; ^1^H NMR (D_2_O, 400 MHz) *δ* 8.55 (m, 1H, ArH), 7.95 (m, 2H, ArH), 7.56 (m, 1H, ArH), 3.41 (t, *J* = 6.4 Hz, 2H), 3.17 (t, *J* = 6.6 Hz, 2H), 1.62 (m, 4H); ^13^C NMR (100 MHz, D_2_O) *δ* 167.2, 156.6, 149.0, 148.7, 138.4, 127.1, 122.3, 40.6, 38.9, 25.6, 25.2; HR-ESI-MS (positive mode) *m/z*: 236.1515 [M + H]^+^ (calculated for C_11_H_18_N_5_O, 236.1511); Purity (HPLC): 95.1%.

*N*-(4-guanidinobutyl)-3-pyridinecarboxamide (**7b**). Yield 85%, white solid. Mp: 190–192 °C; ^1^H NMR (D_2_O, 400 MHz) *δ* 8.76 (d, *J* = 1.7 Hz, 1H, ArH), 8.60 (dd, *J* = 1.7, 5.0 Hz, 1H, ArH), 8.10 (m, 1H, ArH), 7.51 (dd, *J* = 7.7, 5.0 Hz, 1H, ArH), 3.34 (t, *J* = 6.1 Hz, 2H), 3.14 (t, *J* = 6.4 Hz, 2H), 1.59 (m, 4H); ^13^C NMR (100 MHz, D_2_O) *δ* 168.0, 156.6, 150.8, 146.7, 136.3, 130.2, 124.3, 40.6, 39.3, 25.5, 25.2; HR-ESI-MS (positive mode) *m/z*: 236.1517 [M + H]^+^ (calculated for C_11_H_18_N_5_O, 236.1511); Purity (HPLC): 97.7%.

*N*-(4-guanidinobutyl)-4-pyridinecarboxamide (**7c**). Yield 71%, white solid. Mp: 108–110 °C; ^1^H NMR (D_2_O, 400 MHz) *δ* 8.62 (d, *J* = 6.2 Hz, 2H, ArH), 7.64 (d, *J* = 6.2 Hz, 2H, ArH), 3.38 (t, *J* = 6.3 Hz, 2H), 3.17 (t, *J* = 6.5 Hz, 2H), 1.62 (m, 4H); ^13^C NMR (100 MHz, D_2_O) *δ* 168.6, 156.6, 149.4 (2C), 142.2, 121.5 (2C), 40.6, 39.3, 25.4, 25.2; HR-ESI-MS (positive mode) *m/z*: 236.1524 [M+H]^+^ (calculated for C_11_H_18_N_5_O, 236.1511); Purity (HPLC): 99.7%.

#### Procedure for the synthesis of 9a–9u

Compounds **8a**–**8u** (1.16 mmol) and trifluoroacetic acid (1.16 mmol) were dissolved in Dichloromethane (10 mL). The resulting reaction mixture was heated at 50 °C for 24 h. After completion of the reaction, allowed it to room temperature and filtered. The crude product was recrystallized from MeOH to yield the final compounds **9a**–**9u**.

*N*-(3-guanidinobutyl)-2-hydroxybenzamide (**9a**). Yield 75%, white solid. Mp: 118–120 °C; ^1^H NMR (D_2_O, 400 MHz) *δ* 7.66 (d, *J* = 7.9 Hz, 1H, ArH), 7.43 (t, *J* = 7.6 Hz, 1H, ArH), 6.97 (m, 2H, ArH), 3.45 (t, *J* = 6.6 Hz, 2H), 3.24 (t, *J* = 6.6 Hz, 2H), 1.87 (qui, *J* = 6.6 Hz, 2H); ^13^C NMR (100 MHz, D_2_O) *δ* 170.8, 158.0, 157.6, 134.9, 129.1, 120.9, 117.9, 117.4, 39.6, 37.5, 28.5; HR-ESI-MS (positive mode) *m/z:* 237.1378 [M + H]^+^ (calculated for C_11_H_17_N_4_O_2_, 237.1352); Purity (HPLC): 98.6%.

*N*-(3-guanidinobutyl)-3-hydroxybenzamide (**9b**). Yield 82%, white solid. Mp: 130–132 °C; ^1^H NMR (D_2_O, 400 MHz) *δ* 7.35 (t, *J* = 7.8 Hz, 1H, ArH), 7.25 (d, *J* = 7.8 Hz, 1H, ArH), 7.17 (s, 1H, ArH), 7.05 (d, *J* = 7.8 Hz, 1H, ArH), 3.43 (t, *J* = 6.7 Hz, 2H), 3.24 (t, *J* = 6.7 Hz, 2H), 1.87 (qui, *J* = 6.7 Hz, 2H); ^13^C NMR (100 MHz, D_2_O) *δ* 171.9, 158.0, 157.0, 136.5, 131.5, 120.24, 120.20, 115.1, 39.9, 38.2, 28.8; HR-ESI-MS (positive mode) *m/z:* 237.1378 [M + H]^+^ (calculated for C_11_H_17_N_4_O_2_, 237.1352); Purity (HPLC): 99.2%.

*N*-(3-guanidinobutyl)-4-hydroxybenzamide (**9c**). Yield 80%, white oil. ^1^H NMR (D_2_O, 400 MHz) *δ* 7.63 (d, *J* = 8.6 Hz, 2H, ArH), 6.90 (d, *J* = 8.6 Hz, 2H, ArH), 3.39 (t, *J* = 6.8 Hz, 2H), 3.21 (t, *J* = 6.8 Hz, 2H), 1.84 (m, 2H); ^13^C NMR (100 MHz, D_2_O) *δ* 170.4, 159.1, 156.7, 129.1 (2C), 125.3, 115.3 (2C), 38.6, 36.8, 27.5; HR-ESI-MS (positive mode) *m/z:* 237.1374 [M + H]^+^ (calculated for C_11_H_17_N_4_O_2_, 237.1352); Purity (HPLC): 97.8%.

*N*-(4-guanidinobutyl)-3-methoxy-4-hydroxybenzamide (**9d**). Yield 77%, white oil. ^1^H NMR (D_2_O, 400 MHz) *δ* 7.20 (m, 2H, ArH), 6.86 (d, *J* = 8.2 Hz, 1H, ArH), 3.81 (s, 3H), 3.36 (t, *J* = 6.7 Hz, 2H), 3.19 (t, *J* = 6.7 Hz, 2H), 1.82 (qui, *J* = 6.7 Hz, 2H); ^13^C NMR (100 MHz, D_2_O) *δ* 170.8, 158.0, 149.8, 148.3, 126.6, 122.1, 116.1, 111.9, 56.9, 40.1, 38.3, 29.1; HR-ESI-MS (positive mode) *m/z:* 267.1474 [M + H]^+^ (calculated for C_12_H_19_N_4_O_3_, 267.1457); Purity (HPLC): 99.4%.

*N*-(3-guanidinobutyl)-2,4-dihydroxybenzamide (**9e**). Yield 83%, white solid. Mp: 178–180 °C; ^1^H NMR (D_2_O, 400 MHz) *δ* 7.58 (d, *J* = 8.7 Hz, 1H, ArH), 6.43 (dd, *J* = 2.4, 8.7 Hz, 1H, ArH), 6.37 (d, *J* = 2.4 Hz, ArH), 3.41 (t, *J* = 6.7 Hz, 2H) 3.22 (t, *J* = 6.6 Hz, 2H), 1.85 (m, 2H); ^13^C NMR (100 MHz, D_2_O) *δ* 169.6, 160.6, 160.0, 156.5, 129.3, 107.8, 107.6, 102.6, 38.6, 36.3, 27.6; HR-ESI-MS (positive mode) *m/z:* 253.1297 [M + H]^+^ (calculated for C_11_H_17_N_4_O_3_, 253.1301); Purity (HPLC): 99.8%.

*N*-(3-guanidinobutyl)-3,4-dihydroxybenzamide (**9f**). Yield 74%, white oil. ^1^H NMR (D_2_O, 400 MHz) *δ* 7.10 (br.s, 1H, ArH), 7.03 (d, *J* = 7.0 Hz, 1H, ArH), 6.76 (d, *J* = 7.0 Hz, 1H, ArH), 3.22 (br.s, 2H), 3.05 (br.s, 2H), 1.69 (br.s, 2H); ^13^C NMR (100 MHz, D_2_O) *δ* 169.4, 156.4, 147.7, 143.6, 125.2, 120.1, 115.3, 114.5, 38.6, 36.8, 27.6; HR-ESI-MS (positive mode) *m/z:* 253.1331 [M + H]^+^ (calculated for C_11_H_17_N_4_O_3_, 253.1301); Purity (HPLC): 97.8%.

*N*-(3-guanidinobutyl)-3,5-dihydroxybenzamide (**9g**). Yield 72%, white solid. Mp: 68–70 °C; ^1^H NMR (D_2_O, 400 MHz) *δ* 6.68 (d, *J* = 2.2 Hz, 2H, ArH), 6.48 (t, *J* = 2.2 Hz, 1H, ArH), 3.35 (t, *J* = 6.8 Hz, 2H), 3.18 (t, *J* = 6.7 Hz, 2H), 1.81 (m, 2H); ^13^C NMR (100 MHz, D_2_O) *δ* 170.2, 157.0 (2C), 156.7, 136.2, 106.2 (2C), 105.8, 38.6, 36.9, 27.4; HR-ESI-MS (positive mode) *m/z:* 253.1348 [M + H]^+^ (calculated for C_11_H_17_N_4_O_3_, 253.1301); Purity (HPLC): 99.3%.

*N*-(3-Guanidinobutyl)-3,4,5-trihydroxybenzamide (**9h**). Yield 73%, white solid. Mp: 77–79 °C; ^1^H NMR (D_2_O, 400 MHz) *δ* 6.74 (s, 2H, ArH), 3.23 (t, *J* = 6.8 Hz, 2H), 3.08 (t, *J* = 6.8 Hz, 2H), 1.71 (qui, *J* = 6.8 Hz, 2H); ^13^C NMR (100 MHz, D_2_O) *δ* 169.6, 156.6, 144.6 (2C), 136.2, 124.8, 107.3 (2C), 38.7, 36.9, 27.6; HR-ESI-MS (positive mode) *m/z:* 269.1272 [M + H]^+^ (calculated for C_11_H_17_N_4_O_4_, 269.1250); Purity (HPLC): 92.8%.

*N*-(4-guanidinobutyl)-2-hydroxybenzamide (**9i**). Yield 80%, white oil. ^1^H NMR (400 MHz, CD_3_OD) *δ* 7.60 (d, *J* = 7.4 Hz, 1H, ArH), 7.20 (t, *J* = 8.2 Hz, 1H, ArH), 6.73 (m, 2H, ArH), 3.27 (t, *J* = 6.2 Hz, 2H), 3.06 (t, *J* = 6.3 Hz, 2H), 1.51 (m, 4H); ^13^C NMR (100 MHz, CD_3_OD) *δ* 171.2, 161.3, 158.8, 134.8, 129.0, 120.2, 118.6, 117.2, 42.2, 39.9, 27.8, 27.4; HR-ESI-MS (positive mode) *m/z:* 251.1523 [M + H]^+^ (calculated for C_12_H_19_N_4_O_2_, 251.1508); Purity (HPLC): 96.1%.

*N*-(4-guanidinobutyl)-3-hydroxybenzamide (**9j**). Yield 83%, white solid. Mp: 84–86 °C; ^1^H NMR (400 MHz, CD_3_OD) *δ* 7.17 (m, 3H, ArH), 6.87 (br.s, 1H, ArH), 3.32 (br.s, 2H), 3.15 (br.s, 2H), 1.58 (br.s, 4H); ^13^C NMR (100 MHz, CD_3_OD) *δ* 170.6, 158.9, 158.7, 137.1, 130.7, 119.6, 119.1, 115.3, 42.1, 40.2, 27.8, 27.3; HR-ESI-MS (positive mode) *m/z:* 251.1530 [M + H]^+^ (calculated for C_12_H_19_N_4_O_2_, 251.1508); Purity (HPLC): 96.0%.

*N*-(4-guanidinobutyl)-4-hydroxy-3-methoxybenzamide (**9k**). Yield 85%, white solid. Mp: 105–107 °C; ^1^H NMR (400 MHz, D_2_O) *δ* 7.28 (d, *J* = 2.0 Hz, 1H, ArH), 7.24 (dd, *J* = 2.0, 8.2 Hz, 1H, ArH), 6.89 (d, *J* = 8.2 Hz, 1H, ArH), 3.83 (s, 3H), 3.32 (d, *J* = 6.2 Hz, 2H), 3.15 (d, *J* = 6.4 Hz, 2H), 1.59 (br.s, 4H); ^13^C NMR (100 MHz, D_2_O) *δ* 169.9, 156.6, 148.4, 147.1, 125.7, 120.8, 115.0, 110.9, 55.7, 40.6, 39.2, 25.6, 25.2; HR-ESI-MS (positive mode) *m/z:* 281.1634 [M + H]^+^ (calculated for C_13_H_21_N_4_O_3_, 281.1614); Purity (HPLC): 95.4%.

*N*-(4-guanidinobutyl)-2,4-dihydroxybenzamide (**9l**). Yield 76%, white solid. Mp: 74–76 °C; ^1^H NMR (400 MHz, D_2_O) *δ* 7.45 (d, *J* = 8.8 Hz, 1H, ArH), 6.36 (dd, *J* = 2.4, 8.8 Hz, 1H, ArH), 6.27 (d, *J* = 2.4 Hz, 1H, ArH), 3.24 (br.s, 2H) 3.09 (d, *J* = 5.7 Hz, 2H), 1.53 (br.s, 4H); ^13^C NMR (100 MHz, D_2_O) *δ* 169.6, 160.7, 159.8, 156.6, 129.6, 108.5, 107.8, 102.8, 40.7, 38.7, 25.6, 25.2; HR-ESI-MS (positive mode) *m/z:* 267.1454 [M + H]^+^ (calculated for C_12_H_19_N_4_O_3_, 267.1457); Purity (HPLC): 95.1%.

*N*-(4-guanidinobutyl)-3,4-dihydroxybenzamide (**9m**). Yield 73%, white solid. Mp: 157–159 °C; ^1^H NMR (400 MHz, CD_3_OD) *δ* 7.21 (d, *J* = 2.0 Hz, 1H, ArH), 7.12 (dd, *J* = 8.3, 2,0 Hz, 1H, ArH), 6.73 (d, *J* = 8.3 Hz, 1H, ArH), 3.24 (br.s, 2H) 3.10 (br.s, 2H), 1.51 (m, 4H); ^13^C NMR (100 MHz, CD_3_OD) *δ* 170.5, 158.7, 150.1, 146.2, 127.1, 120.8, 116.1, 115.9, 42.1, 40.2, 27.8, 27.2; HR-ESI-MS (positive mode) *m/z:* 267.1450 [M + H]^+^ (calculated for C_12_H_19_N_4_O_3_, 267.1457); Purity (HPLC): 95.2%.

*N*-(4-guanidinobutyl)-3,5-dihydroxybenzamide (**9n**). Yield 83%, white solid. Mp: 81–83 °C; ^1^H NMR (400 MHz, D_2_O) *δ* 6.68 (d, *J* = 2.2 Hz, 2H, ArH), 6.49 (t, *J* = 2.2 Hz, 1H, ArH), 3.30 (t, *J* = 6.2 Hz, 2H), 3.14 (t, *J* = 6.3 Hz, 2H), 1.57 (br.s, 4H); ^13^C NMR (100 MHz, D_2_O) *δ* 170.1, 157.0 (2C), 156.6, 136.4, 106.1 (2C), 105.8, 40.6, 39.2, 25.5, 25.2; HR-ESI-MS (positive mode) *m/z:* 267.1451 [M + H]^+^ (calculated for C_12_H_19_N_4_O_3_, 267.1457); Purity (HPLC): 98.9%.

*N*-(4-Guanidinobutyl)-3,4,5-trihydroxybenzamide (**9o**). Yield 71%, white solid. Mp: 102–104 °C; ^1^H NMR (400 MHz, D_2_O) *δ* 6.77 (s, 2H, ArH), 3.18 (br.s, 2H), 3.02 (br.s, 2H), 1.46 (br.s, 4H); ^13^C NMR (100 MHz, D_2_O) *δ* 169.5, 156.5, 144.6 (2C), 136.1, 124.9, 107.2 (2C), 40.6, 39.2, 25.6, 25.2; HR-ESI-MS (positive mode) *m/z:* 283.1425 [M + H]^+^ (calculated for C_12_H_19_N_4_O_4_, 283.1406); Purity (HPLC): 96.0%.

*N*-(5-guanidinopentyl)-2-hydroxybenzamide (**9p**). Yield 82%, white oil. ^1^H NMR (400 MHz, D_2_O) *δ* 7.79 (d, *J* = 7.4 Hz, 1H, ArH), 7.38 (t, 1H, *J* = 7.4 Hz, ArH), 7.11 (m, 2H, ArH), 3.51 (t, *J* = 6.5 Hz, 2H), 3.12 (t, *J* = 7.4 Hz, 2H), 1.81 (m, 4H), 1.57 (m, 2H); ^13^C NMR (100 MHz, D_2_O) *δ* 169.7, 156.9 (2C), 133.8, 128.1, 119.9, 116.9, 116.7, 39.2, 39.0, 27.8, 26.3, 22.9; HR-ESI-MS (positive mode) *m/z:* 265.1678 [M + H]^+^ (calculated for C_13_H_21_N_4_O_2_, 265.1665); Purity (HPLC): 97.6%.

*N*-(5-guanidinopentyl)-3-hydroxybenzamide (**9q**). Yield 86%, white oil. ^1^H NMR (400 MHz, CD_3_OD) *δ* 7.26 (m, 3H, ArH), 6.95 (d, *J* = 7.0 Hz, 1H, ArH), 3.39 (t, *J* = 7.0 Hz, 2H), 3.20 (t, *J* = 7.0 Hz, 2H), 1.66 (m, 4H), 1.46 (m, 2H); ^13^C NMR (100 MHz, CD_3_OD) *δ* 170.5, 158.9, 158.7, 137.3, 130.1, 119.5, 119.0, 115.3, 42.4, 40.6, 30.2, 29.6, 25.0; HR-ESI-MS (positive mode) *m/z:* 265.1668 [M + H]^+^ (calculated for C_13_H_21_N_4_O_2_, 265.1665); Purity (HPLC): 99.9%.

*N*-(5-guanidinopentyl)-4-hydroxybenzamide (**9r**). Yield 79%, white oil. ^1^H NMR (400 MHz, CD_3_OD) *δ* 7.72 (d, *J* = 8.8 Hz, 2H, ArH), 6.84 (t, *J* = 8.8 Hz, 2H, ArH), 3.39 (t, *J* = 7.0 Hz, 1H), 2.94 (t, *J* = 7.6 Hz, 2H), 1.69 (m, 4H), 1.46 (m, 2H); ^13^C NMR (100 MHz, CD_3_OD) *δ* 170.2, 162.1, 158.8, 130.2 (2C), 126.5, 116.1 (2C), 40.6, 40.4, 30.1, 28.2, 24.8; HR-ESI-MS (positive mode) *m/z:* 265.1672 [M + H]^+^ (calculated for C_13_H_21_N_4_O_2_, 265.1665); Purity (HPLC): 96.7%.

*N*-(5-guanidinopentyl)-4-hydroxy-3-methoxybenzamide (**9s**). Yield 85%, white oil. ^1^H NMR (400 MHz, CD_3_OD) *δ* 7.45 (d, *J* = 2.0 Hz, 1H, ArH), 7.37 (dd, *J* = 2.0*,* 8.2 Hz, 1H, ArH), 6.85 (d, *J* = 8.2 Hz, 1H, ArH), 3.91 (s, 3H), 3.39 (t, *J* = 7.0 Hz, 2H), 3.19 (t, *J* = 7.1 Hz, 2H), 1.65 (m, 4H), 1.45 (m, 2H); ^13^C NMR (100 MHz, CD_3_OD) *δ* 170.0, 158.7, 151.2, 148.8, 126.9, 122.0, 115.9, 111.9, 56.5, 42.4, 40.7, 30.3, 29.6, 25.1; HR-ESI-MS (positive mode) *m/z:* 295.1788 [M + H]^+^ (calculated for C_14_H_23_N_4_O_3_, 295.1770); Purity (HPLC): 95.4%.

*N*-(5-guanidinopentyl)-3,5-dihydroxybenzamide (**9t**). Yield 78%, white solid. Mp: 88–90 °C; ^1^H NMR (400 MHz, CD_3_OD) *δ* 6.72 (d, *J* = 2.2 Hz, 2H, ArH), 6.44 (t, *J* = 2.2 Hz, 1H, ArH), 3.36 (t, *J* = 7.1 Hz, 2H), 3.19 (t, *J* = 7.1 Hz, 2H), 1.65 (m, 4H), 1.44 (m, 2H); ^13^C NMR (100 MHz, CD_3_OD) *δ* 170.7, 159.9(2C), 158.7, 138.0, 106.7(2C), 106.5, 42.4, 40.6, 30.1, 29.5, 25.0; HR-ESI-MS (positive mode) *m/z:* 281.1626 [M + H]^+^ (calculated for C_13_H_21_N_4_O_3_,281.1614); Purity (HPLC): 99.8%.

*N*-(5-guanidinobutyl)-3,4,5-trihydroxybenzamide (**9u**). Yield 72%, white solid. Mp: 93–95 °C; ^1^H NMR (400 MHz, D_2_O) *δ* 6.80 (s, 2H, ArH), 3.22 (t, *J* = 6.9 Hz, 2H), 3.05 (t, *J* = 6.9 Hz, 2H), 1.49 (m, 4H), 1.28 (m, 2H); ^13^C NMR (100 MHz, D_2_O) *δ* 169.7, 156.5, 144.7 (2C), 136.1, 125.2, 107.3 (2C), 40.8, 39.5, 27.8, 27.4, 23.0; HR-ESI-MS (positive mode) *m/z:* 297.1602 [M+H]^+^ (calculated for C_13_H_21_N_4_O_4_, 297.1563); Purity (HPLC): 96.6%.

#### General procedure for the synthesis of intermediates 12a–12e

To a solution of compounds **11a**–**11e** in MeOH (10 mL), 12% NaOH (10 mL) solution was added. The resulting mixture was stirred at room temperature for 10 min. The reaction progress was detected by TLC. After concentration, the crude product was obtained and purified by silica gel column chromatography using ethyl acetate as eluent to give compounds **12a**–**12e**.

#### Procedure for the synthesis of 13a–13e

Compounds **13a**–**13e** were synthesized by using the same way for **7a**–**7c**. Nevertheless, compounds **12a**–**2e** was used instead of compounds **6a**–**6c**.

3-Guanidinopropyl 3-hydroxybenzoate (**13a**). Yield 77%, white solid. Mp: 140–142 °C; ^1^H NMR (400 MHz, CD_3_OD) *δ* 7.52 (dt, *J* = 7.9, 1.4 Hz, 1H, ArH), 7.45 (dd, *J* = 2.4, 1.4 Hz, 1H, ArH), 7.31 (t, *J* = 7.9 Hz, 1H, ArH), 7.05 (ddd, *J* = 7.9, 2.4, 1.4 Hz, 1H, ArH), 4.40 (t, *J* = 6.2 Hz, 2H), 3.39 (t, *J* = 6.9 Hz, 2H), 2.08 (m, 2H); ^13^C NMR (100 MHz, CD_3_OD) *δ* 168.0, 158.9, 158.8, 132.5, 130.7, 121.6, 121.4, 117.1, 63.1, 39.5, 29.2; HR-ESI-MS (positive mode) *m/z:* 238.1196 [M + H]^+^ (calculated for C_11_H_16_N_3_O_3_, 238.1192); Purity (HPLC): 99.8%.

3-Guanidinopropyl 4-hydroxybenzoate (**13b**). Yield 73%, white oil. ^1^H NMR (400 MHz, CD_3_OD) *δ* 7.90 (d, *J* = 8.7 Hz, 2H), 6.85 (d, *J* = 8.7 Hz, 2H), 4.36 (t, *J* = 5.9 Hz, 2H), 3.40 (t, *J* = 6.8 Hz, 2H), 2.08 (m, 2H); ^13^C NMR (100 MHz, CD_3_OD) *δ* 168.0, 163.0, 158.1, 132.6 (2C), 121.7, 116.0 (2C), 62.7, 39.4, 28.8; HR-ESI-MS (positive mode) *m/z:* 238.1189 [M + H]^+^ (calculated for C_11_H_15_N_3_O_3_, 238.1192); Purity (HPLC): 99.8%.

3-Guanidinopropyl 4-hydroxy-3-methoxybenzoate (**13c**). Yield 88%, white oil. ^1^H NMR (400 MHz, CD_3_OD) *δ* 7.58 (dd, *J* = 8.3, 1.9 Hz, 1H, ArH), 7.53 (d, *J* = 1.9 Hz, 1H, ArH), 6.86 (d, *J* = 8.3 Hz, 1H, ArH), 4.37 (t, *J* = 6.1 Hz, 2H), 3.91 (s, 3H), 3.40 (t, *J* = 6.9 Hz, 2H), 2.09 (m, 2H); ^13^C NMR (100 MHz, CD_3_OD) *δ* 168.1, 158.1, 154.4, 148.4, 125.0 (2C), 115.8, 113.1, 62.8, 56.5, 39.4, 28.9; HR-ESI-MS (positive mode) *m/z:* 268.1302 [M + H]^+^ (calculated for C_12_H_18_N_3_O_4_, 268.1297); Purity (HPLC): 99.8%.

3-Guanidinopropyl 3,5-dihydroxybenzoate (**13d**). Yield 79%, white solid. Mp: 70–72 °C; ^1^H NMR (400 MHz, CD_3_OD) *δ* 6.95 (d, *J* = 2.2 Hz, 2H, ArH), 6.50 (t, *J* = 2.2 Hz, 1H, ArH), 4.37 (t, *J* = 6.2 Hz, 2H), 3.38 (t, *J* = 6.9 Hz, 2H), 2.07 (m, 2H); ^13^C NMR (100 MHz, CD_3_OD) *δ* 168.0, 159.7 (2C), 158.5, 132.9, 108.8 (2C), 108.3, 63.0, 39.4, 29.1; HR-ESI-MS (positive mode) *m/z:* 254.1147 [M + H]^+^ (calculated for C_11_H_16_N_3_O_4_, 254.1141); Purity (HPLC): 98.3%.

4-Guanidinobutyl 4-hydroxybenzoate (**13e**). Yield 78%, white solid. Mp: 79–81 °C; ^1^H NMR (400 MHz, CD_3_OD) *δ* 7.88 (d, *J* = 8.8 Hz, 2H, ArH), 6.85 (d, *J* = 8.8 Hz, 2H, ArH), 4.31 (t, *J* = 6.1 Hz, 2H), 3.29 (t, *J* = 7.0 Hz, 2H), 1.85 (m, 2H), 1.76 (m, 2H); ^13^C NMR (100 MHz, CD_3_OD) *δ* 168.2, 163.1, 158.1, 132.6 (2C), 121.9, 116.0 (2C), 65.1, 42.0, 26.8, 26.3; HR-ESI-MS (positive mode) *m/z:* 252.1362 [M + H]^+^ (calculated for C_12_H_18_N_3_O_3_, 252.1348); Purity (HPLC): 99.7%.

### Molecular docking

Molecular docking was carried out using Discovery Studio 2017 R2 (Accelrys, San Diego, USA). Docking studies were conducted with DS CDOCKER program. The cryoelectron microscopy (cryo-EM) structure of NHE-1 from Homo sapiens (PBD ID: 7DSX) was obtained from the Protein Data Bank. All water molecules and allied ligand were removed followed by protein preparation protocol with the CHARMm force field. The simulated annealing parameters were set as follows: heating steps and cooling steps were set to 2000 and 5000, respectively, while heating and cooling temperatures were set to 700 and 300, respectively. Other parameters were kept as default. Ten top-ranked conformations for each docked compound were retained and visually inspected for binding pattern analysis using Discovery Studio Visualizer.

### Biological activity

#### Cell culture and cell viability assay

The H9c2 cardiomyocyte (rat myocardial cell line) and HEK 293 cell (Human Embryonic Kidney 293 cells) were obtained from ATCC (American type culture collection) and grown in Dulbecco’s modifed Eagle’s medium (DMEM) (Wuhan Pricella Biotechnology Co., Ltd.) with 10% fetal bovine serum (FBS, Wuhan Pricella Biotechnology Co., Ltd.) and 1% penicillin–streptomycin (Beijing Solarbio Science & Technology Co.,Ltd.) at 37 ℃ in a humidified incubator consisting of 5% CO_2_ and 95% air. After reaching about 70–80% confluence, the H9c2 cardiomyocyte was digested by trypsinization (0.25% Trypsin–EDTA solution, Beijing Solarbio Science&Technology Co.,Ltd) every three days. After seeded into the 96-well culture plates for 24 h with 3000 cells/well, the cells were divided into control group, Dox treated group, Dex treated group, buthutin A treated group, and compounds-treated group. The cells in Dox-treated group were treated with 1 μM Dox for 24 h, while the Dex-treated group, buthutin A treated group, and compounds-treated groups were treated with 20 μM Dex and 1 μM Dox, 1 μM buthutin A and 1 μM Dox, and 1 μM target compound and 1 μM Dox for 24 h, respectively. At the same time, control group cells were incubated continuously with the normal medium. After treatment, the MTT solution (Beijing Solarbio Science&Technology Co., Ltd.) with the final concentration of 0.5 mg/ml was added into each well and incubated for 4 h at 37 ℃. After formazan product in each well was dissolved, the absorbance of each well was measured at 570 nm by using a microplate reader (BioTek Instruments, Inc.) [21]. The protection ratio of each compound was calculated as (A_compound_–A_blank_)/(A_control_–A_blank_) × 100%.

#### Measurement of intracellular pH (pHi)

The pHi of cells was measured by the pH-sensitive fluorescent probe [2′,7′-bis-(2-carboxyethyl)-5-(and-6)-carboxyfluorescein, acetoxymethyl ester (BCECF-AM, Beyotime)] [29]. After seeded into the 96-well plate for 24 h with 10,000 cells/well, H9c2 cardiomyocyte was incubated with Krebs Solution for 30 min, and then the diluted BCECF-AM with the final concentration of 1 μM was added into each well for another 30 min. The BCECF-AM was washed up with Krebs Solution and incubated for 30 min, the fluorescence value was measured with Spectra Max iD3 (Molecular Devices) at 480 nm and 440 nm for excitation and 530 nm for emission, then fluorescence intensity ratio (FIR) was calculated as FIR_480 nm_/FIR_440 nm_ × 100%. The pHi standard curve was made as follows: five wells with almost the same values of FIR were selected, and added the high-potassium solution containing 4 mg/L nigericin sodium salt (Beijing psaitong Biotechnology Co., Ltd) with different pH gradients (6.5, 6.8, 7.1, 7.4 and 7.7), and incubated for 8 min; then the pHi standard curve was derived based on the pH value and corresponding FIR value. Then, the H9c2 cardiomyocyte was divided into control group, model group, cariporide treated group (1 μM), buthutin A treated group (1 μM), and compounds treated group (1 μM). Except the control group, the cells of other groups were incubated with 25 mM NH_4_Cl diluted with Krebs Solution for 3 min, and then the cells were washed with sodium-free Krebs Solution to clean NH_4_Cl. Target compound diluted with sodium-free Krebs Solution was added to the cells and cultured for 10 min. Then the target compound was washed with sodium-containing Krebs Solution, and the FIR value of each well was detected. The pHi values of each group were calculated according to the standard pHi curve. The activity of NHE-1 is expressed as the rate of pHi change per minute (dpHi/min) from the sodium-free to sodium-containing phase. In addition, the HEK293 cells were divided into control group, model group, cariporide (3 µM)-treated group, cariporide (3 µM) and buthutin A (1 µM)-treated group, cariporide (3 µM) and **9k** (1 µM)-treated group, cariporide (3 µM) and **9m** (1 µM)-treated group, and cariporide (3 µM) and **9o** (1 µM)-treated group. All these groups received the intracellular pH (pHi) measurement to estimate the NHEs activity.

### Statistical analysis

The data of this research were expressed as mean ± SEM, which were analyzed with SPSS 19.0 and GraphPad Prism 7.0. All tests were carried out at least in quintuplicate. The statistical comparisons in the different group were performed using one-way ANOVA followed by Tukey's post hoc test. Differences were considered to have statistical significance at *p* < 0.05.

## Supplementary Information

Supplementary material 1.

## Data Availability

All data generated or analyzed during this study are included in this published article and its Supporting Information files.
