# Supplementary material for: Design, synthesis and biological evaluation of buthutin derivatives as cardioprotective agents
Source: Nat Prod Bioprospect. 2025 Feb 5;15(1):14. doi: 10.1007/s13659-025-00497-9 (PMC11799461; doi:10.1007/s13659-025-00497-9)
Supplement: Supplementary file 1 — Supplementary material 1. [file 13659_2025_497_MOESM1_ESM.pdf]

Table of Contents

Compound **7a** 1
^{1}H-NMR 1
^{13}C-NMR 1
HR- ESI -MS $[M+H]^+$ 2
HPLC analysis 2
Compound **7b** 3
^{1}H-NMR 3
^{13}C-NMR 3
HR- ESI -MS $[M+H]^+$ 4
HPLC analysis 4
Compound **7c** 5
^{1}H-NMR 5
^{13}C-NMR 5
HR- ESI -MS $[M+H]^+$ 6
HPLC analysis 6
Compound **9a** 7
^{1}H-NMR 7
^{13}C-NMR 7
HR- ESI -MS $[M+H]^+$ 8
HPLC analysis 8
Compound **9b** 9
^{1}H-NMR 9
^{13}C-NMR 9
HR- ESI -MS $[M+H]^+$ 10
HPLC analysis 10
Compound **9c** 11
^{1}H-NMR 11
^{13}C-NMR 11

HR- ESI -MS $[M+H]^+$ 12
HPLC analysis 12
Compound **9d** 13
^{1}H-NMR 13
^{13}C-NMR 13
HR- ESI -MS $[M+H]^+$ 14
HPLC analysis 14
Compound **9e** 15
^{1}H-NMR 15
^{13}C-NMR 15
HR- ESI -MS $[M+H]^+$ 16
HPLC analysis 16
Compound **9f** 17
^{1}H-NMR 17
^{13}C-NMR 17
HR- ESI -MS $[M+H]^+$ 18
HPLC analysis 18
Compound **9g** 19
^{1}H-NMR 19
^{13}C-NMR 19
HR- ESI -MS $[M+H]^+$ 20
HPLC analysis 20
Compound **9h** 21
^{1}H-NMR 21
^{13}C-NMR 21
HR- ESI -MS $[M+H]^+$ 22
HPLC analysis 22
Compound **9i** 23
^{1}H-NMR 23
^{13}C-NMR 23
HR- ESI -MS $[M+H]^+$ 24

HPLC analysis 24
Compound **9j** 25
^{1}H-NMR 25
^{13}C-NMR 25
HR- ESI -MS $[M+H]^+$ 26
HPLC analysis 26
Compound **9k** 27
^{1}H-NMR 27
^{13}C-NMR 27
HR- ESI -MS $[M+H]^+$ 28
HPLC analysis 28
Compound **9l** 29
^{1}H-NMR 29
^{13}C-NMR 29
HR- ESI -MS $[M+H]^+$ 30
HPLC analysis 30
Compound **9m** 31
^{1}H-NMR 31
^{13}C-NMR 31
HR- ESI -MS $[M+H]^+$ 32
HPLC analysis 32
Compound **9n** 33
^{1}H-NMR 33
^{13}C-NMR 33
HR- ESI -MS $[M+H]^+$ 34
HPLC analysis 34
Compound **9o** 35
^{1}H-NMR 35
^{13}C-NMR 35
HR- ESI -MS $[M+H]^+$ 36
HPLC analysis 36

Compound **9p** 37
^{1}H-NMR 37
^{13}C-NMR 37
HR- ESI -MS $[M+H]^+$ 38
HPLC analysis 38
Compound **9q** 39
^{1}H-NMR 39
^{13}C-NMR 39
HR- ESI -MS $[M+H]^+$ 40
HPLC analysis 40
Compound **9r** 41
^{1}H-NMR 41
^{13}C-NMR 41
HR- ESI -MS $[M+H]^+$ 42
HPLC analysis 42
Compound **9s** 43
^{1}H-NMR 43
^{13}C-NMR 43
HR- ESI -MS $[M+H]^+$ 44
HPLC analysis 44
Compound **9t** 45
^{1}H-NMR 45
^{13}C-NMR 45
HR- ESI -MS $[M+H]^+$ 46
HPLC analysis 46
Compound **9u** 47
^{1}H-NMR 47
^{13}C-NMR 47
HR- ESI -MS $[M+H]^+$ 48
HPLC analysis 48
Compound **13a** 49

^{1}H-NMR 49
^{13}C-NMR 49
HR- ESI -MS $[M+H]^+$ 50
HPLC analysis 50
Compound **13b** 51
^{1}H-NMR 51
^{13}C-NMR 51
HR- ESI -MS $[M+H]^+$ 52
HPLC analysis 52
Compound **13c** 53
^{1}H-NMR 53
^{13}C-NMR 53
HR- ESI -MS $[M+H]^+$ 54
HPLC analysis 54
Compound **13d** 55
^{1}H-NMR 55
^{13}C-NMR 55
HR- ESI -MS $[M+H]^+$ 56
HPLC analysis 56
Compound **13e** 57
^{1}H-NMR 57
^{13}C-NMR 57
HR- ESI -MS $[M+H]^+$ 58
HPLC analysis 58

Compound 7a

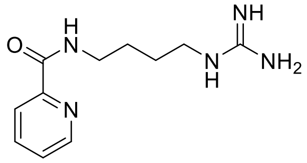

^{1}H-NMR

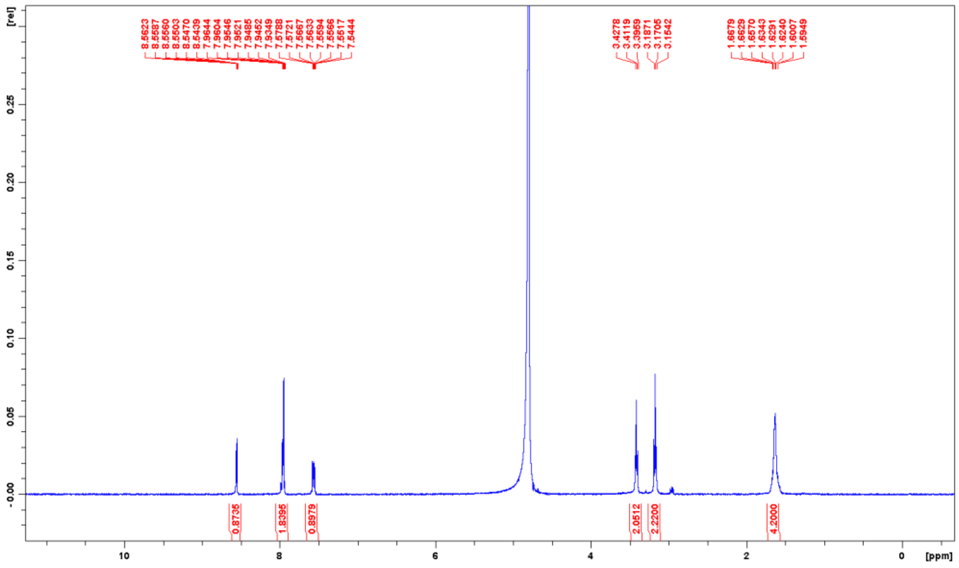

^{13}C-NMR

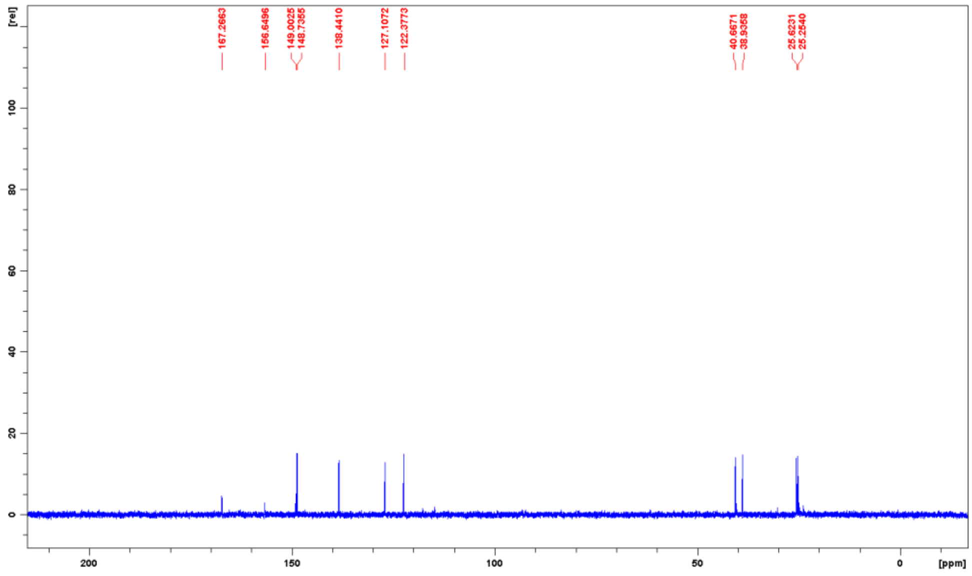

HR- ESI -MS $[M+H]^+$

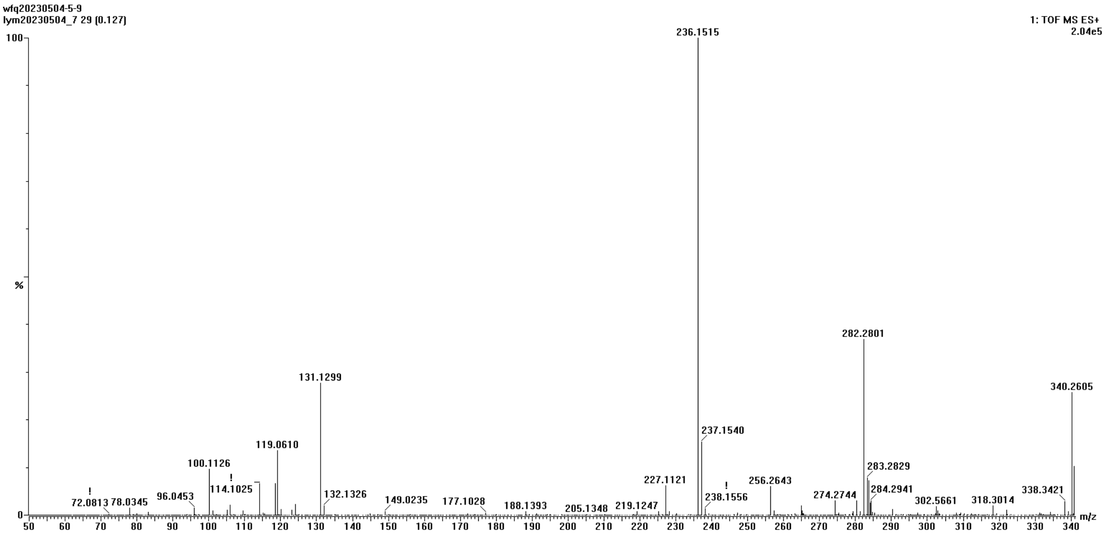

HPLC analysis

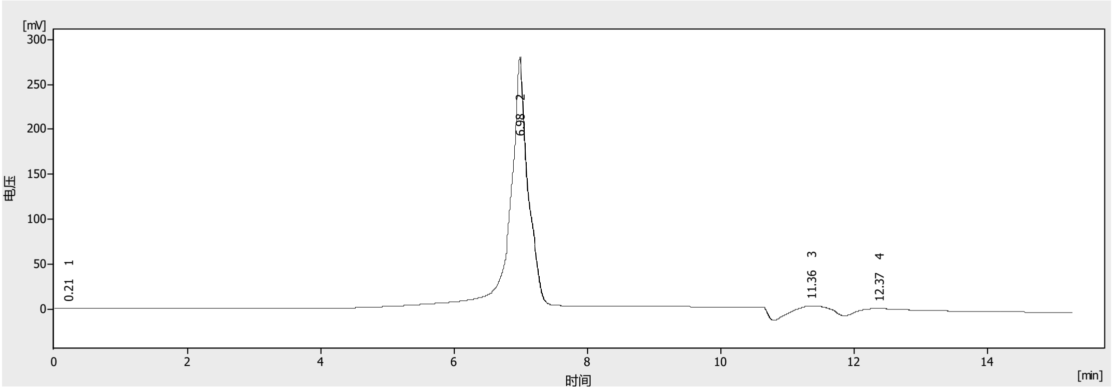

No	Name	Retention/min	Peak area/mV·s	Peak height/mV	Area/%
1	N.A.	0.207	7.370	0.600	0.1
2	N.A.	6.983	5395.507	278.815	95.1
3	N.A.	11.355	169.857	6.483	3.0
4	N.A.	12.367	103.456	2.894	1.8
5	Total		5676.190	288.792	100.0

Compound 7b

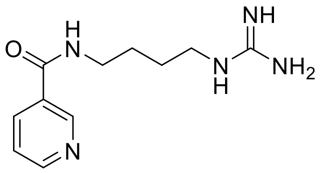

^{1}H-NMR

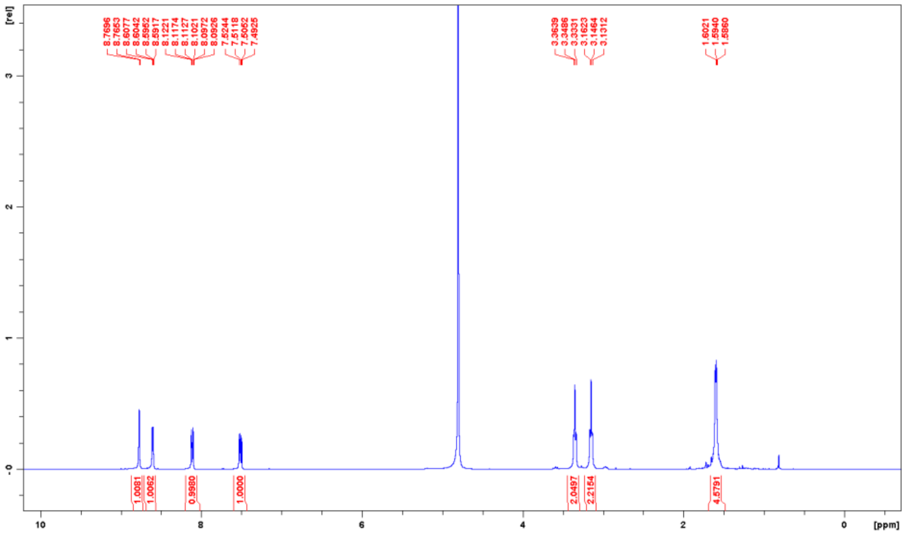

^{13}C-NMR

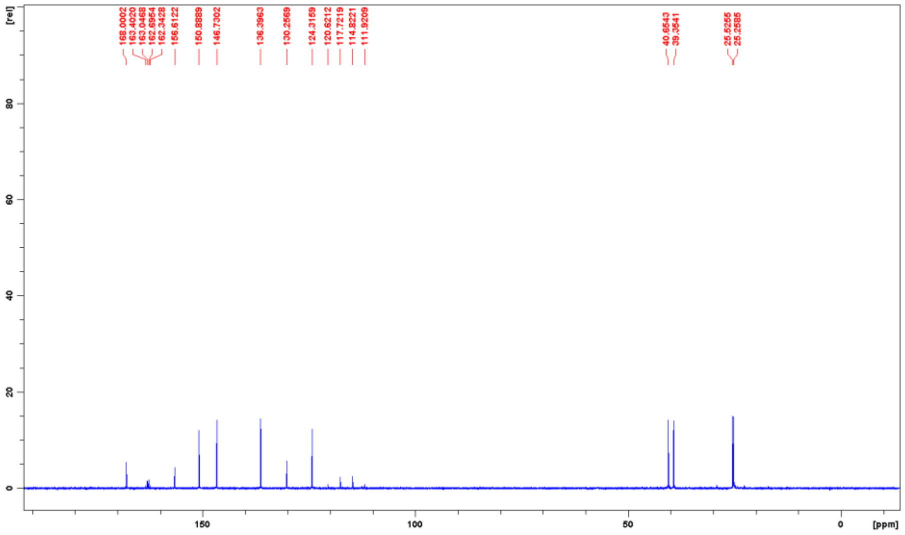

HR- ESI -MS $[M+H]^+$

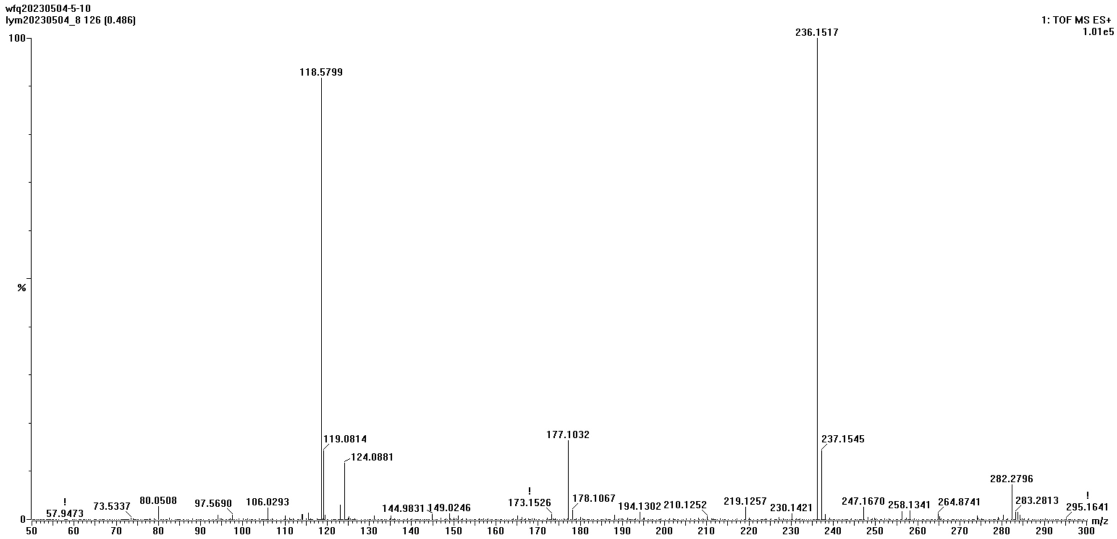

HPLC analysis

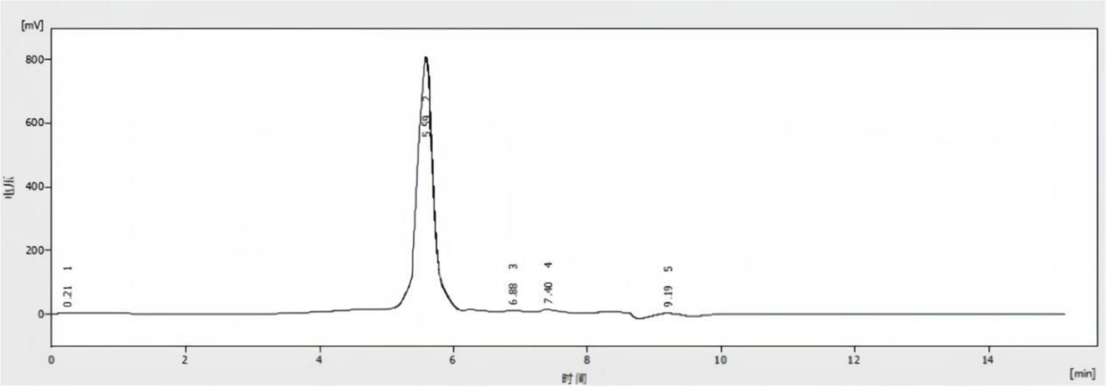

No	Name	Retention/min	Peak area/mV·s	Peak height/mV	Area/%
1	N.A.	5.587	14157.669	802.183	97.7
2	N.A.	6.885	59.417	3.394	0.4
3	N.A.	7.402	130.190	8.000	0.9
4	N.A.	9.193	150.190	7.418	1.0
5	Total		14497.466	820.995	100.0

Compound 7c

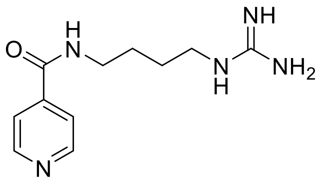

^{1}H-NMR

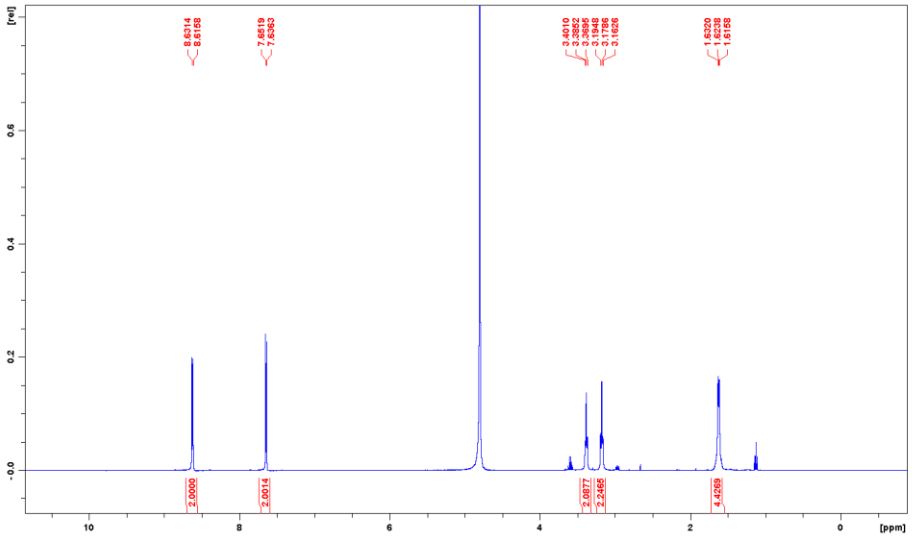

^{13}C-NMR

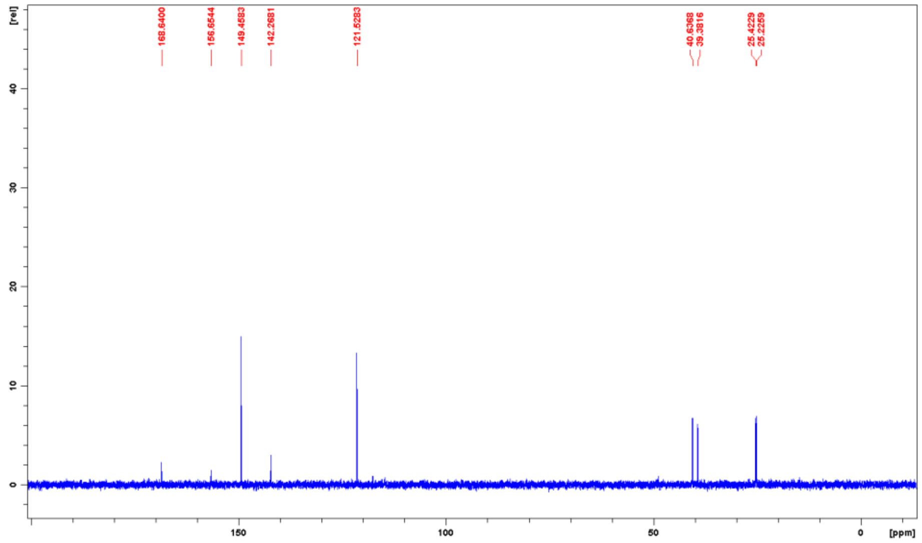

HR- ESI -MS $[M+H]^+$

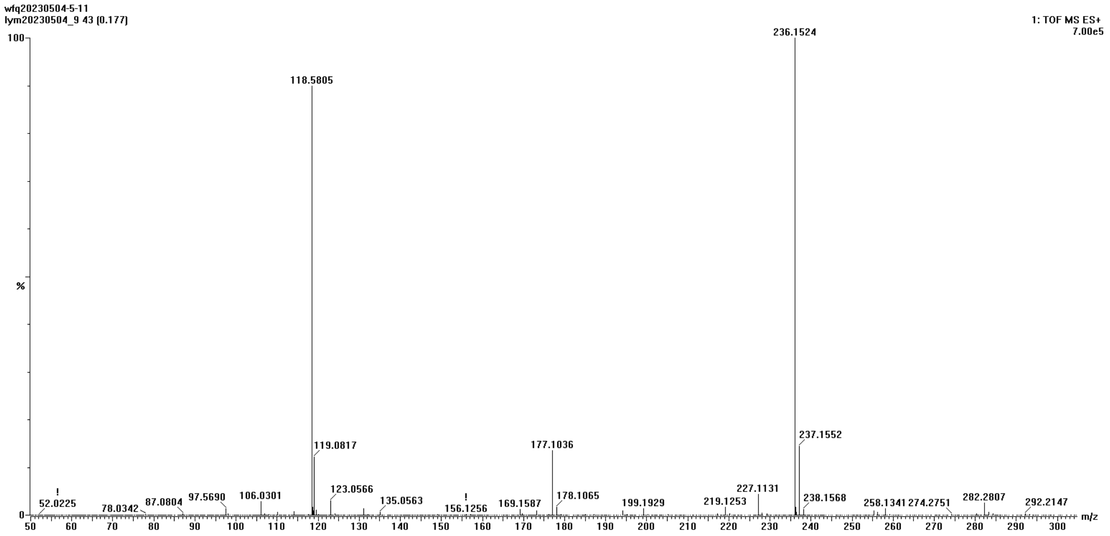

HPLC analysis

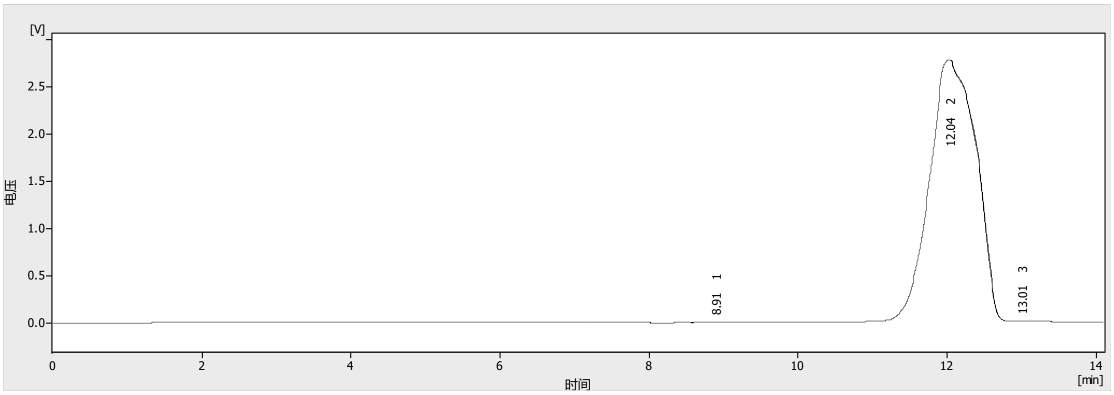

No	Name	Retention/min	Peak area/mV·s	Peak height/mV	Area/%
1	8.907	8.907	24.823	1.217	0.0
2	12.042	12.042	121027.368	2778.460	99.7
3	13.008	13.008	360.500	12.855	0.3
4	Total		121412.691	2792.532	100.0

Compound 9a

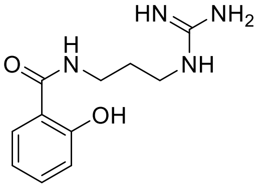

^{1}H-NMR

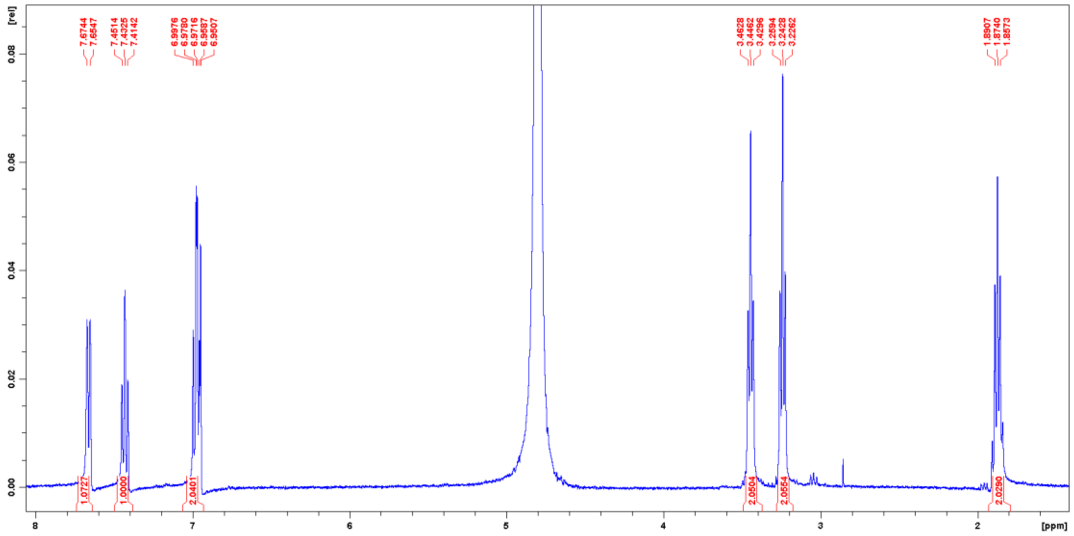

^{13}C-NMR

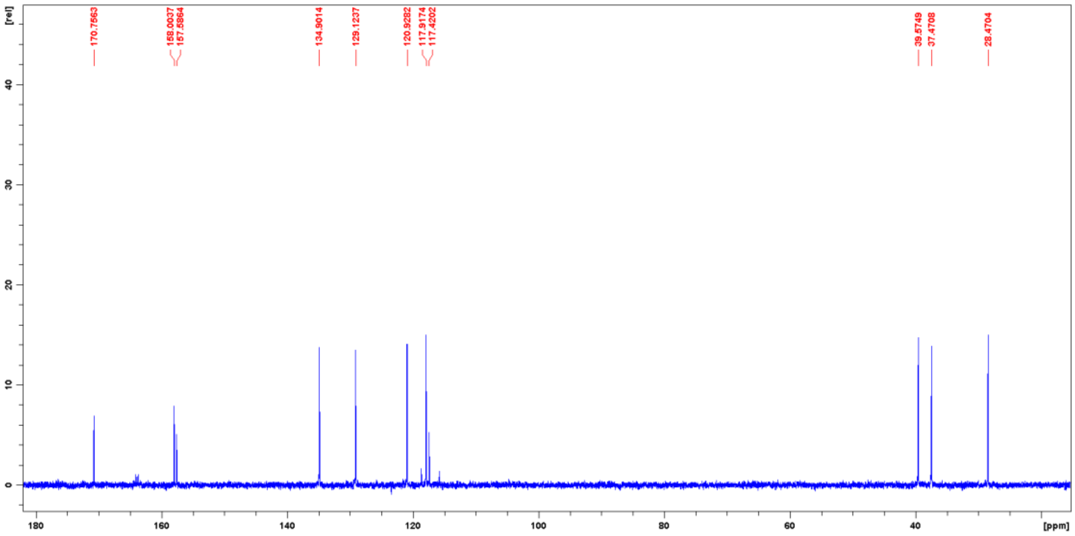

HR- ESI -MS $[M+H]^+$

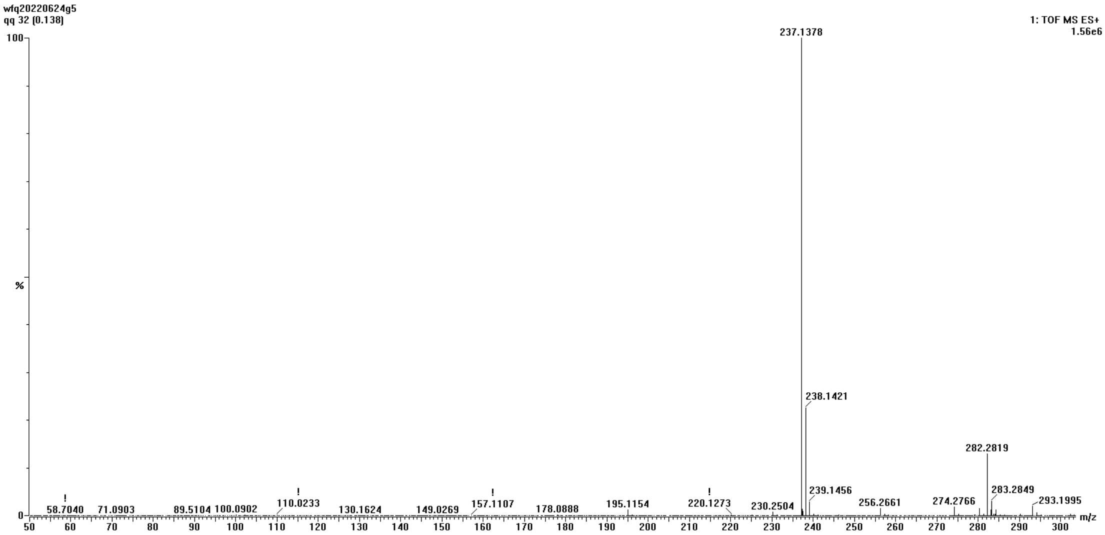

HPLC analysis

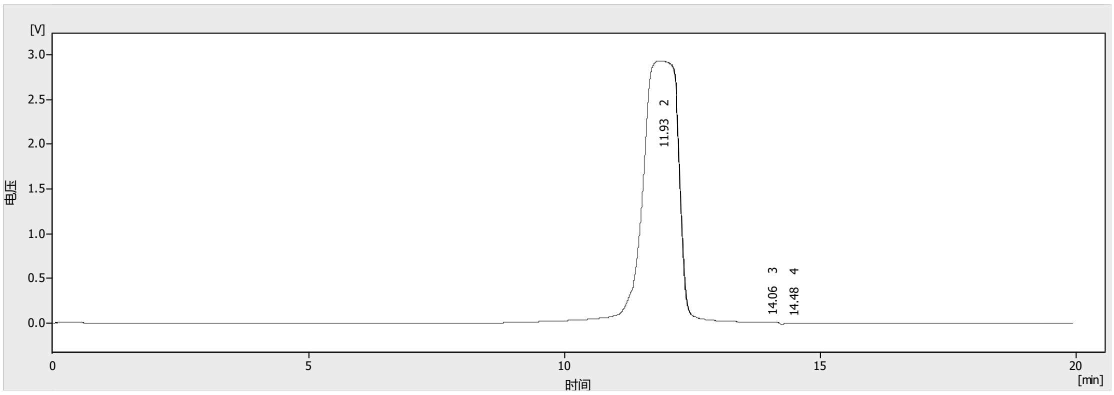

No	Name	Retention/min	Peak area/mV·s	Peak height/mV	Area/%
1	N.A.	0.125	28.427	2.007	0.0
2	N.A.	11.930	145854.188	2940.367	98.6
3	N.A.	14.058	644.645	31.667	0.4
4	N.A.	14.475	1370.675	18.416	0.9
5	Total		147897.935	2992.457	100.0

Compound 9b

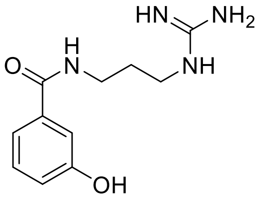

^{1}H-NMR

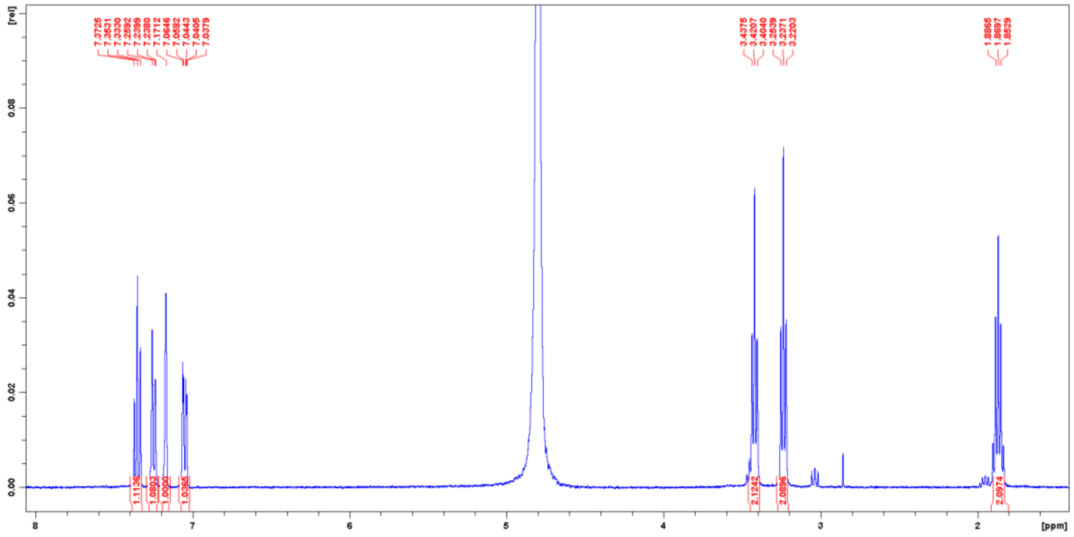

^{13}C-NMR

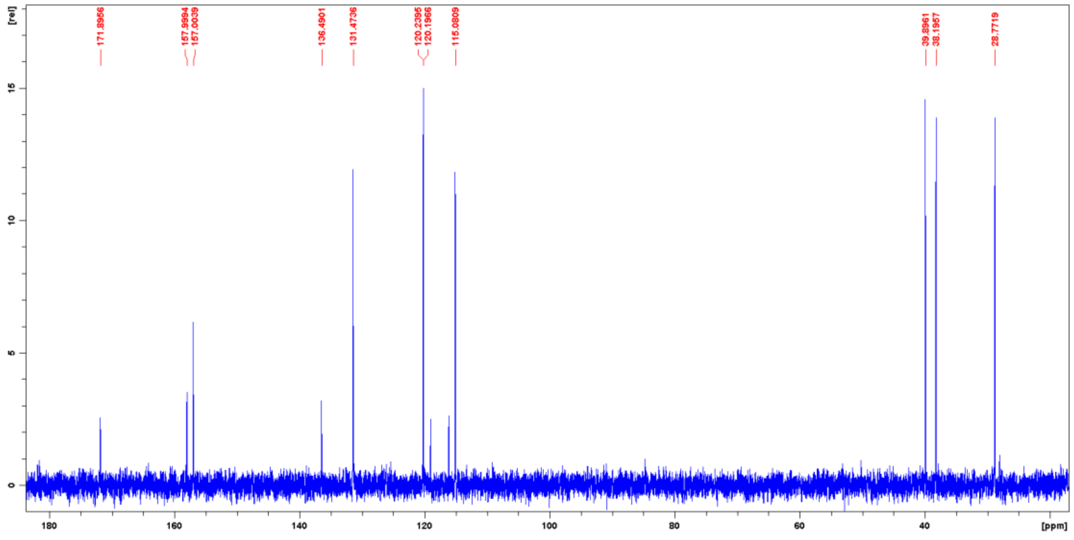

HR- ESI -MS $[M+H]^+$

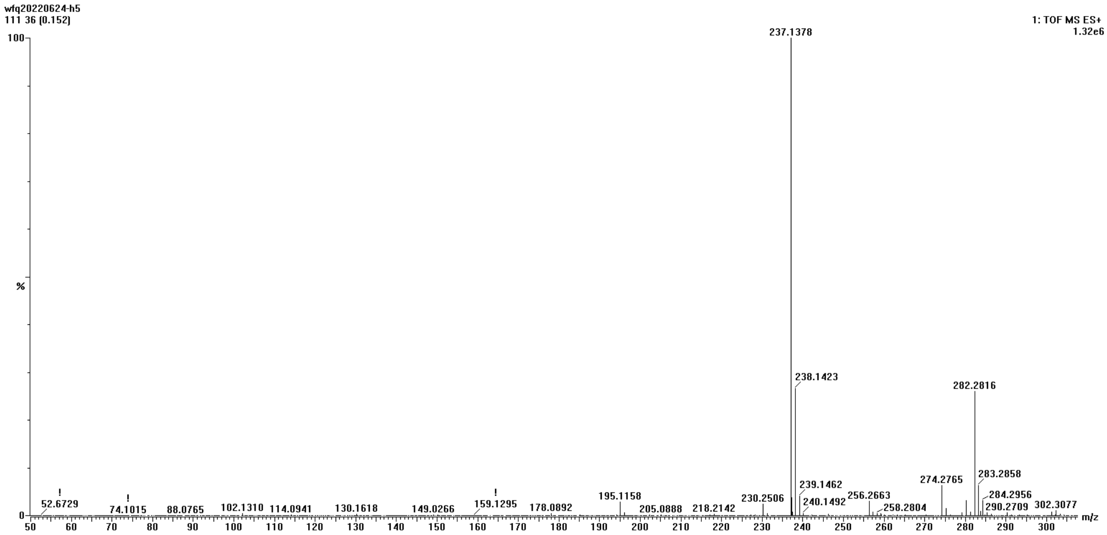

HPLC analysis

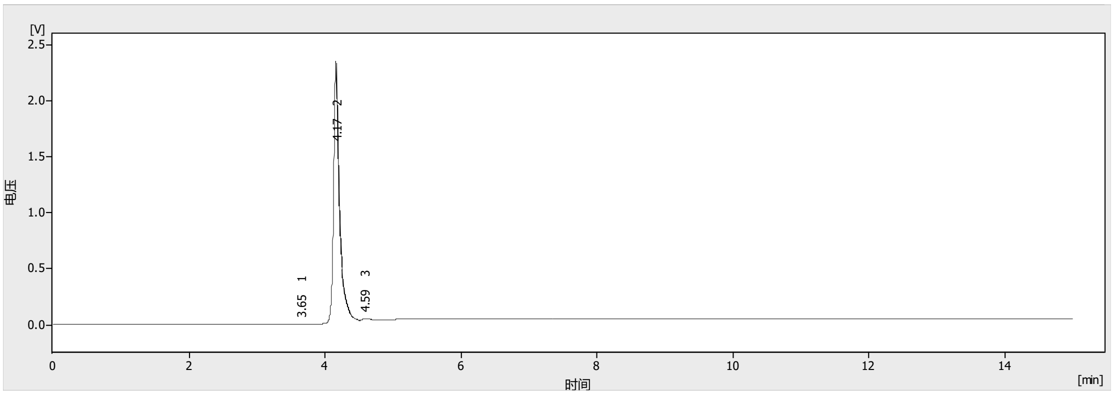

No	Name	Retention/min	Peak area/mV·s	Peak height/mV	Area/%
1	N.A.	4.167	13881.217	2341.664	99.2
2	N.A.	4.585	117.060	18.024	0.8
3	Total		13998.277	2359.688	100.0

Compound 9c

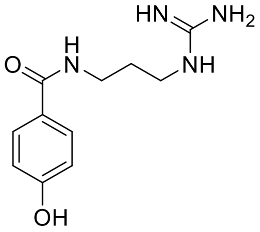

^{1}H-NMR

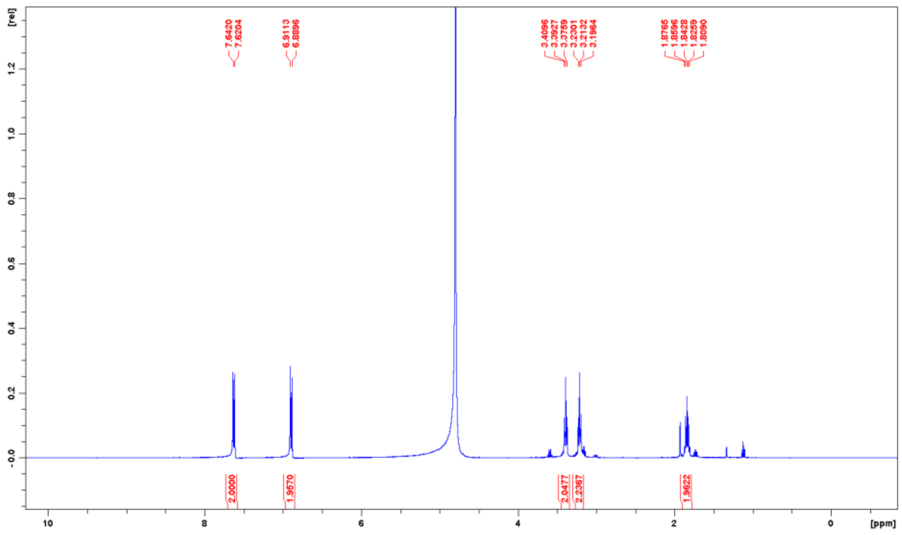

^{13}C-NMR

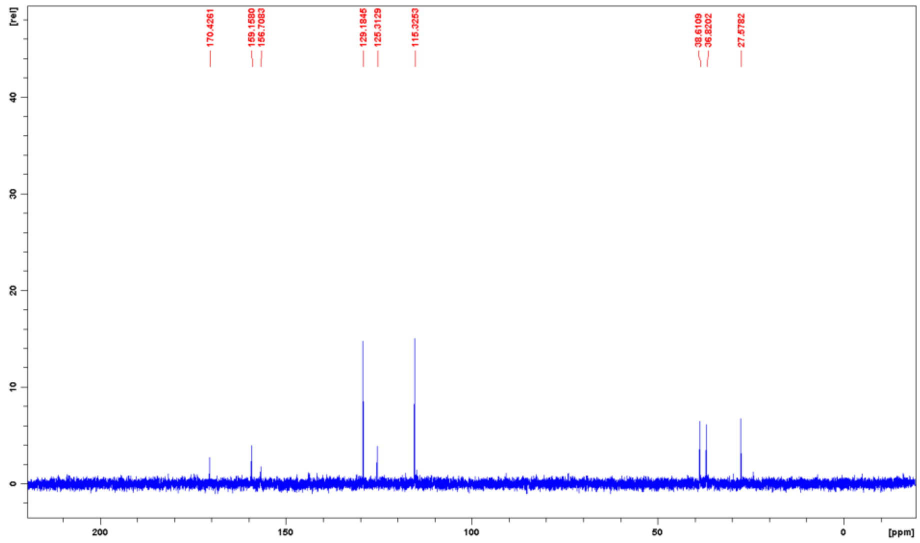

HR- ESI -MS $[M+H]^+$

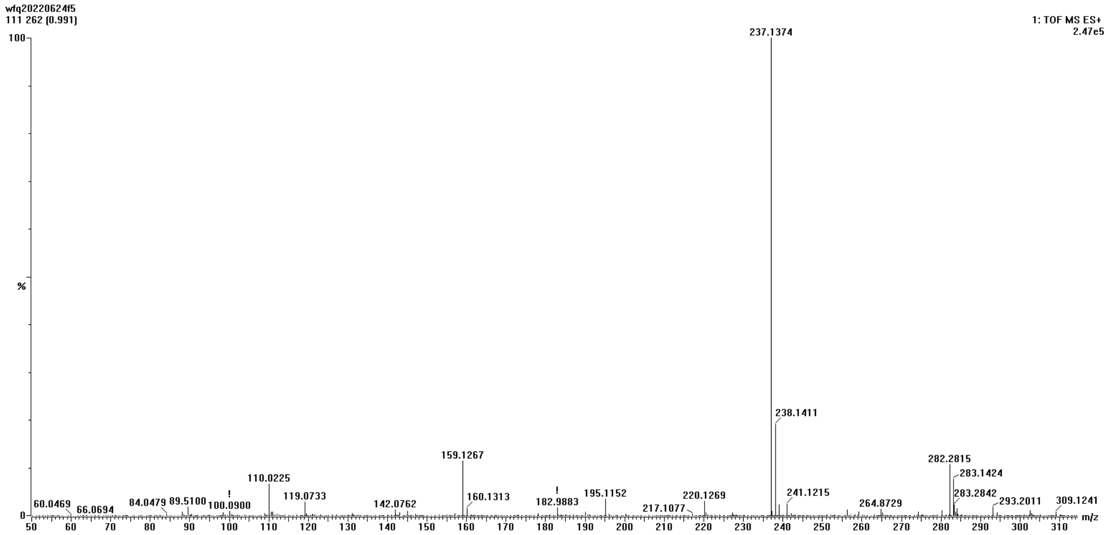

HPLC analysis

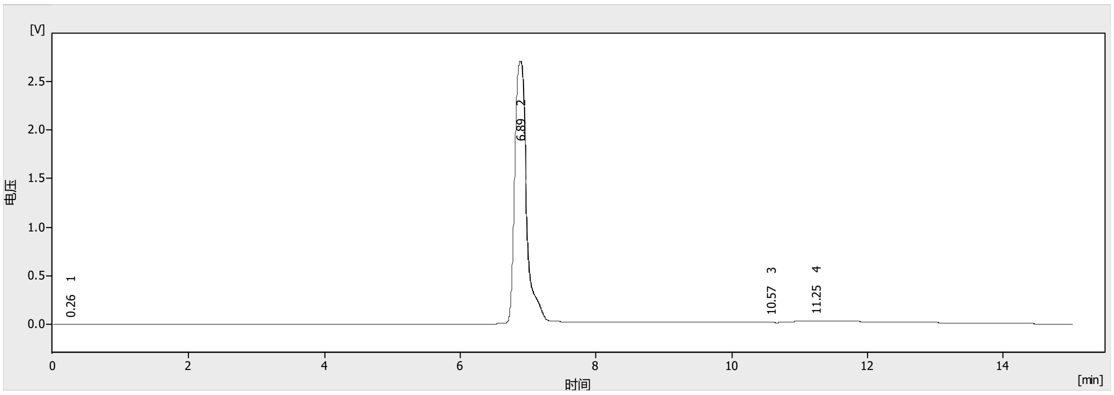

No	Name	Retention/min	Peak area/mV·s	Peak height/mV	Area/%
1	N.A.	0.255	2.777	0.209	0.0
2	N.A.	6.893	33674.676	2701.787	97.8
3	N.A.	10.573	19.125	2.817	0.1
4	N.A.	11.247	738.888	12.360	2.1
5	Total		34435.467	2717.174	100.0

Compound 9d

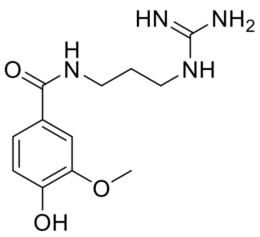

^{1}H-NMR

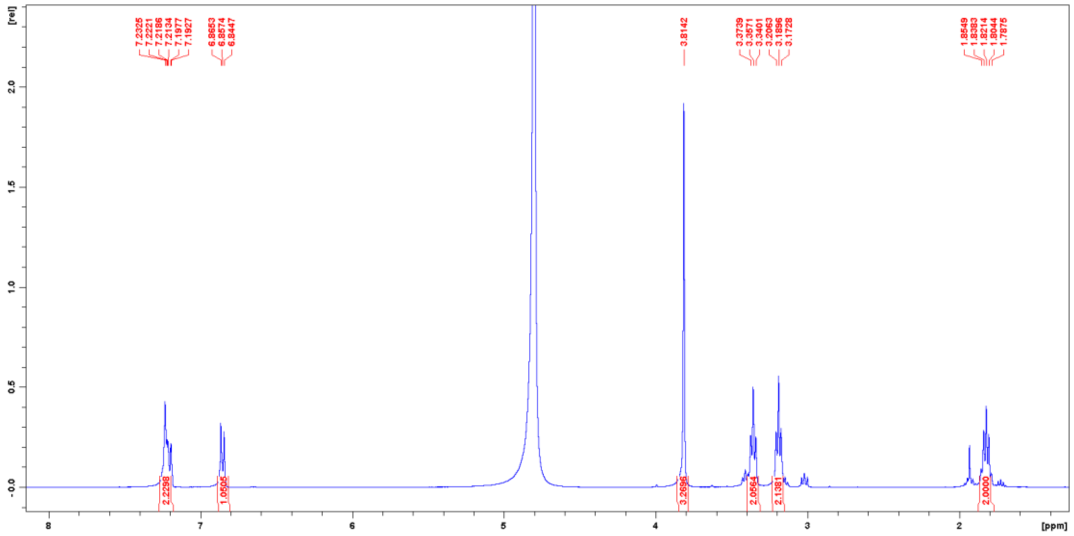

^{13}C-NMR

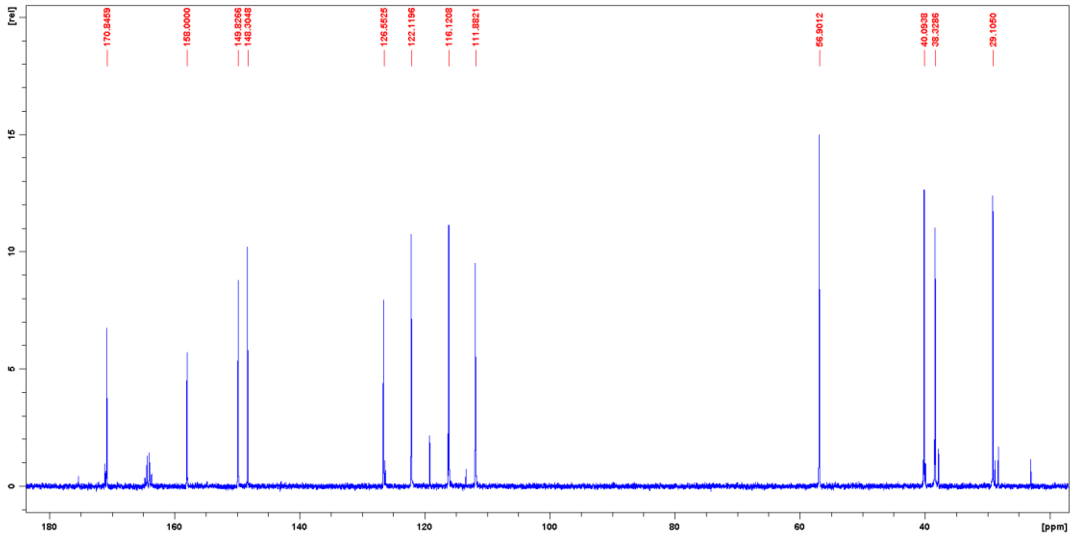

HR- ESI -MS $[M+H]^+$

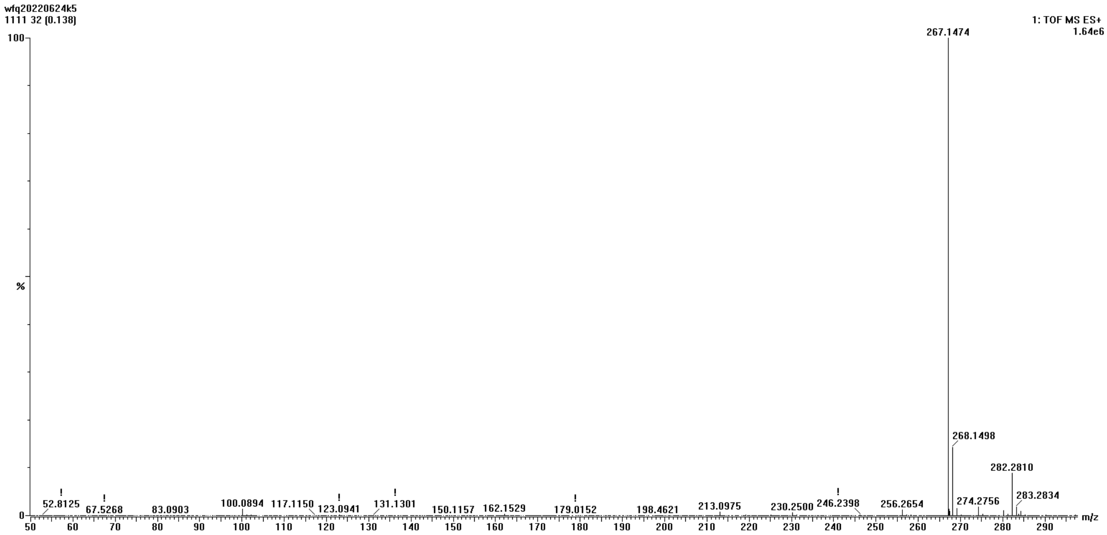

HPLC analysis

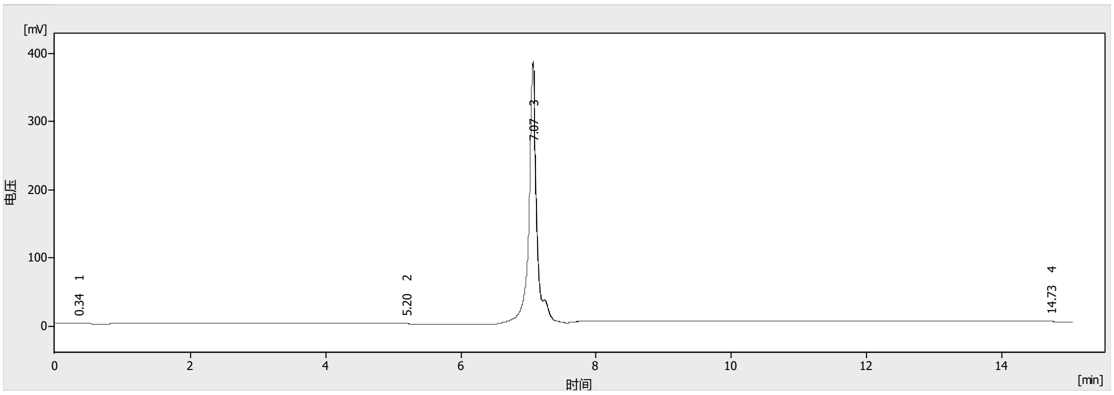

No	Name	Retention/min	Peak area/mV·s	Peak height/mV	Area/%
1	N.A.	0.343	3.982	0.637	0.1
2	N.A.	5.202	2.515	0.610	0.1
3	N.A.	7.068	3225.016	386.209	99.4
4	N.A.	14.727	11.659	1.271	0.4
5	Total		3243.171	388.727	100.0

Compound 9e

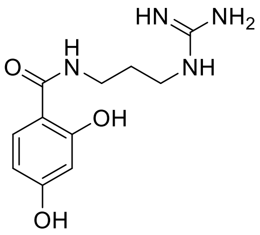

^{1}H-NMR

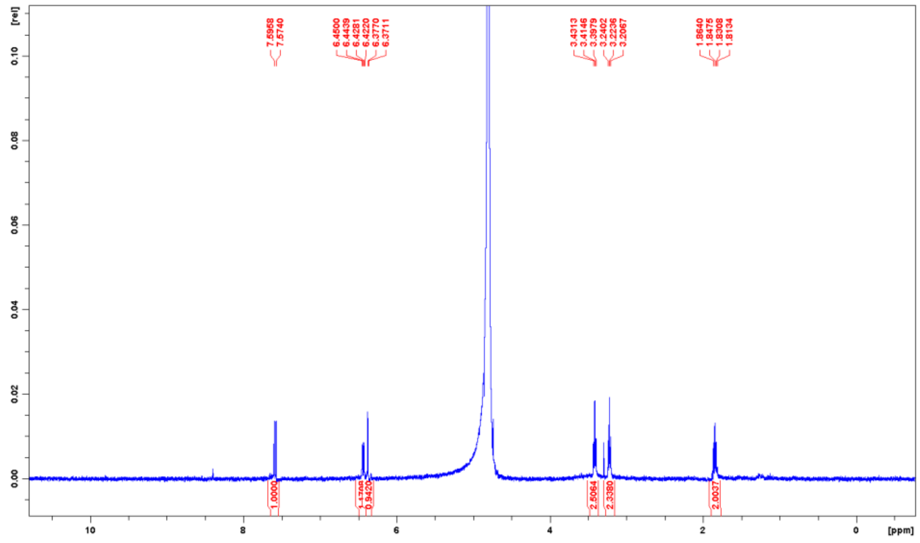

^{13}C-NMR

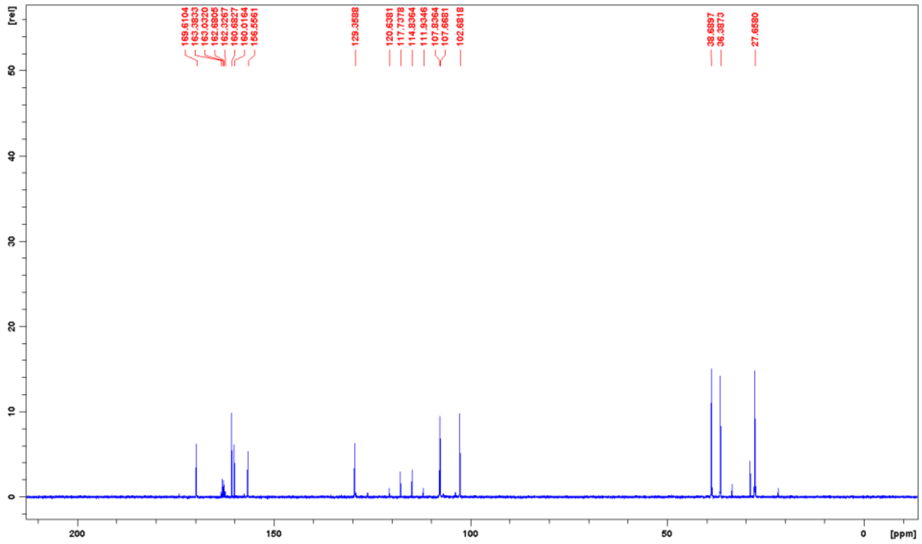

HR- ESI -MS $[M+H]^+$

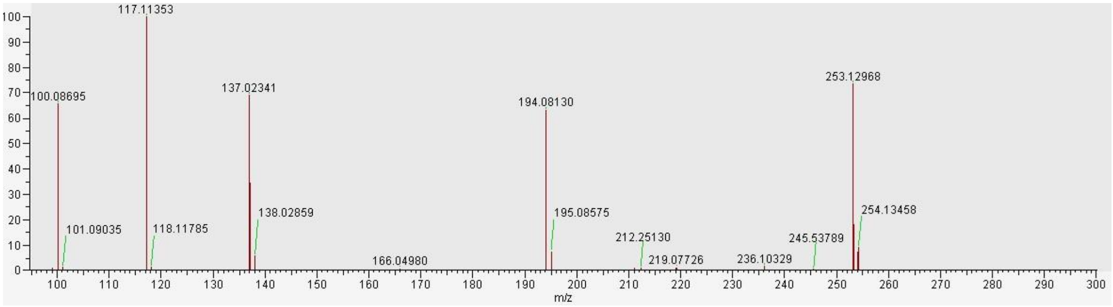

HPLC analysis

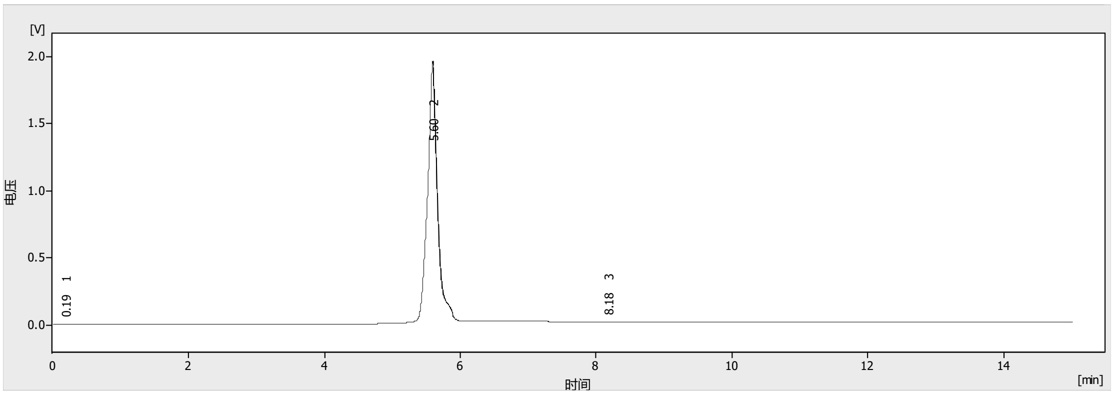

No	Name	Retention/min	Peak area/mV·s	Peak height/mV	Area/%
1	N.A.	5.597	20319.685	1946.750	99.8
2	N.A.	8.175	41.064	1.438	0.2
3	Total		20360.750	1948.188	100.0

Compound 9f

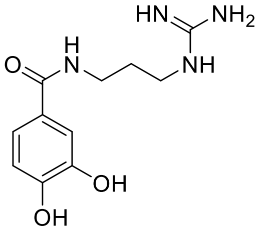

^{1}H-NMR

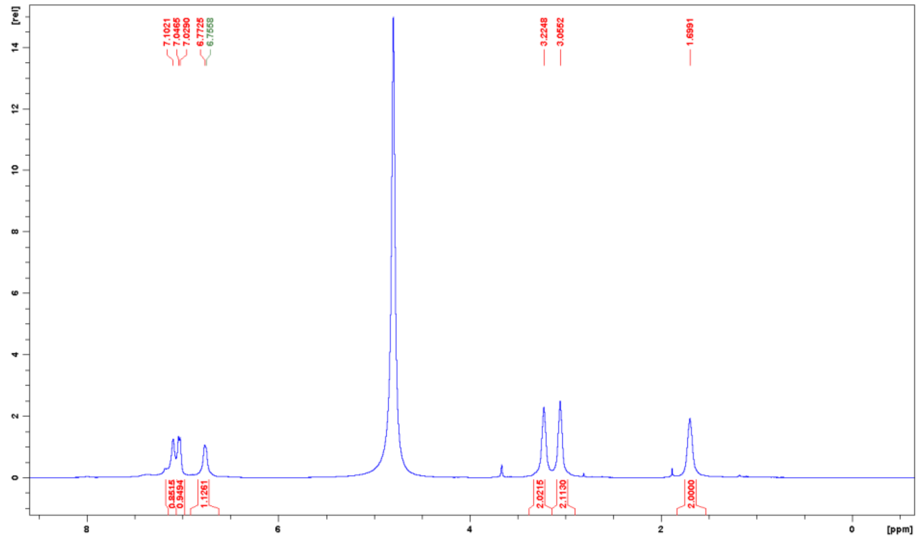

^{13}C-NMR

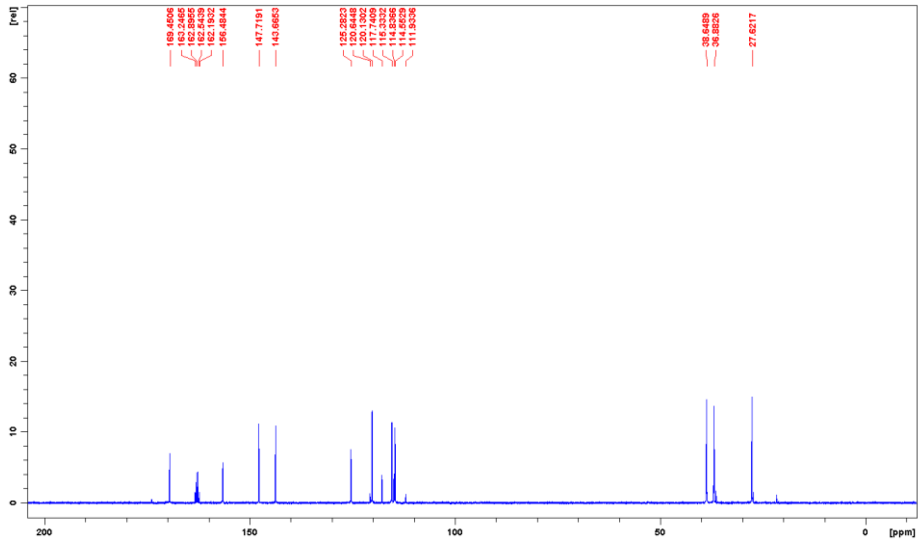

HR- ESI -MS $[M+H]^+$

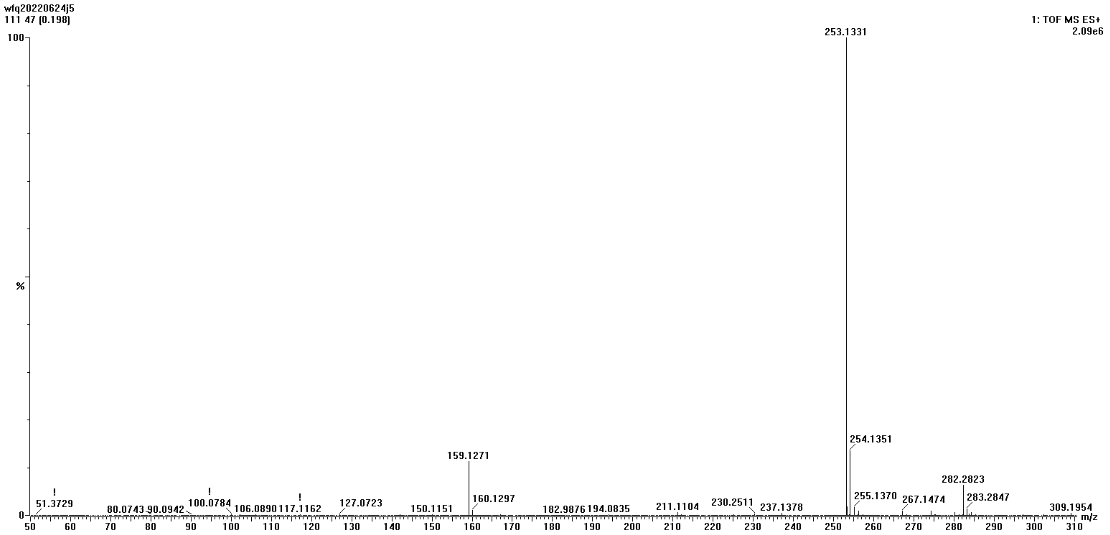

HPLC analysis

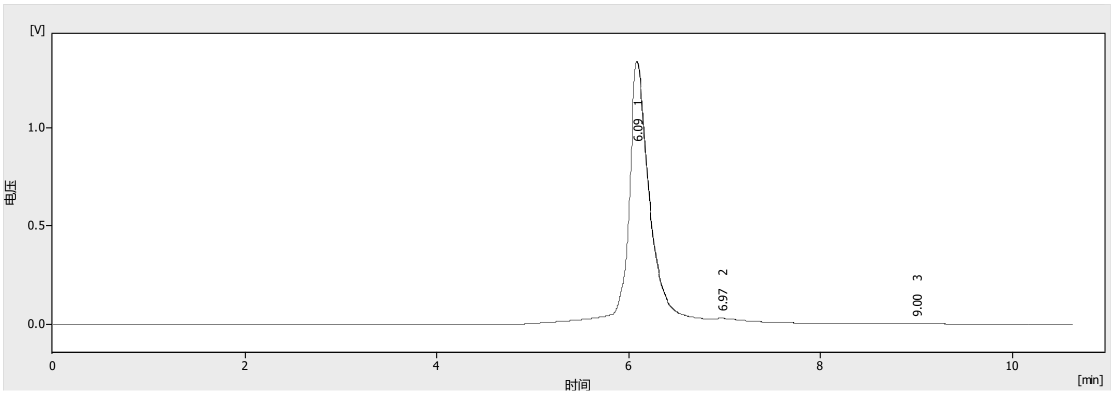

No	Name	Retention/min	Peak area/mV·s	Peak height/mV	Area/%
1	N.A.	6.088	20895.809	1342.371	97.8
2	N.A.	6.972	440.933	21.141	2.1
3	N.A.	9.002	20.631	0.963	0.1
4	Total		21357.374	1364.474	100.0

Compound 9g

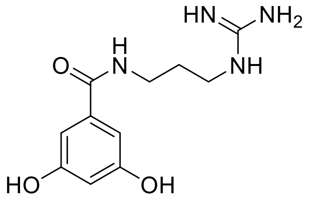

^{1}H-NMR

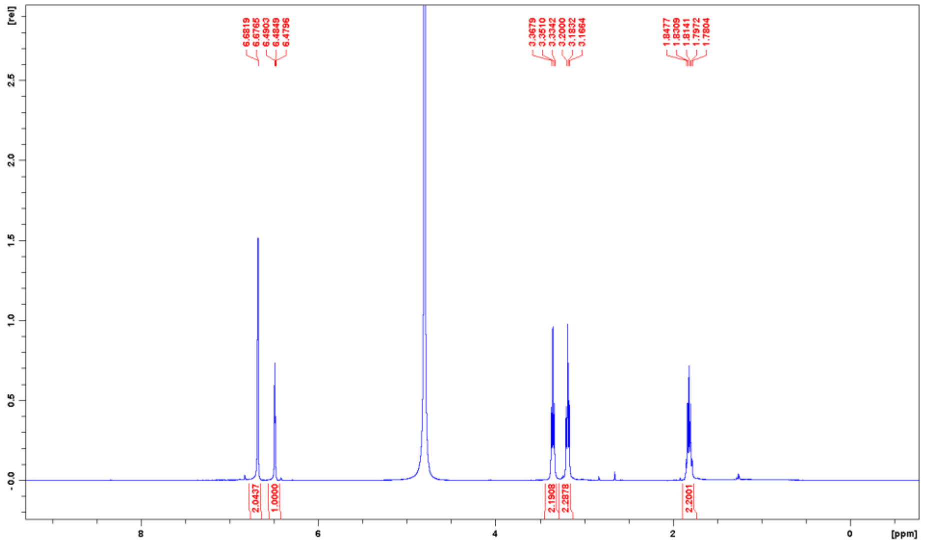

^{13}C-NMR

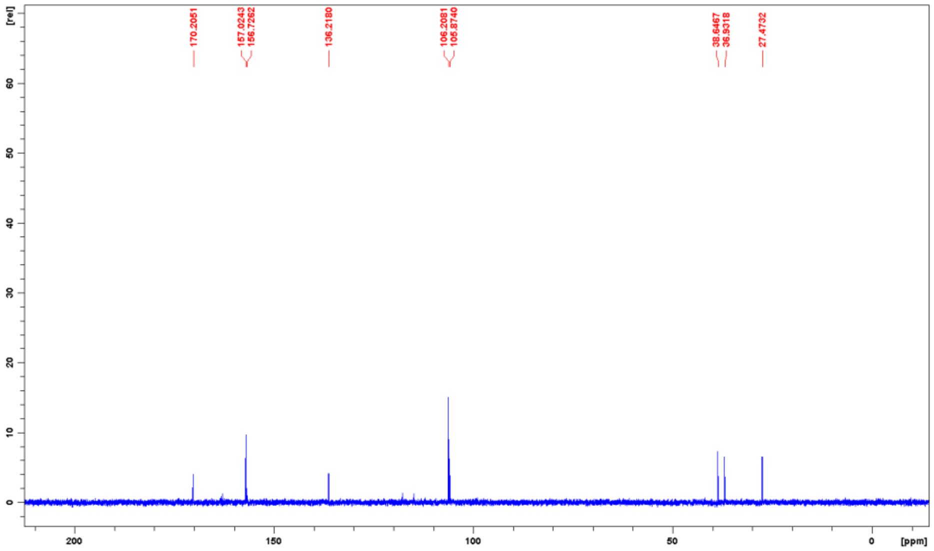

HR- ESI -MS $[M+H]^+$

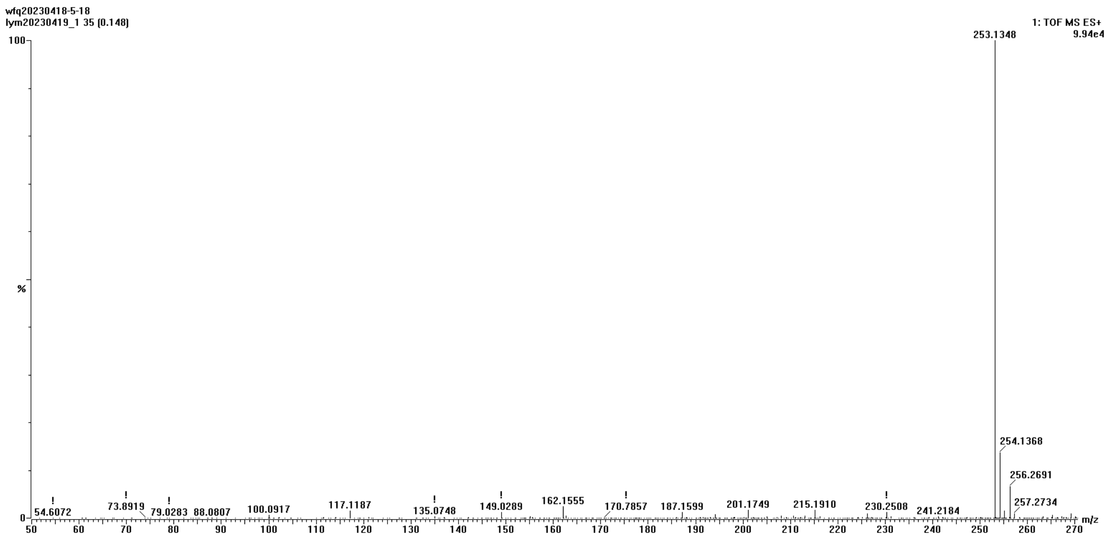

HPLC analysis

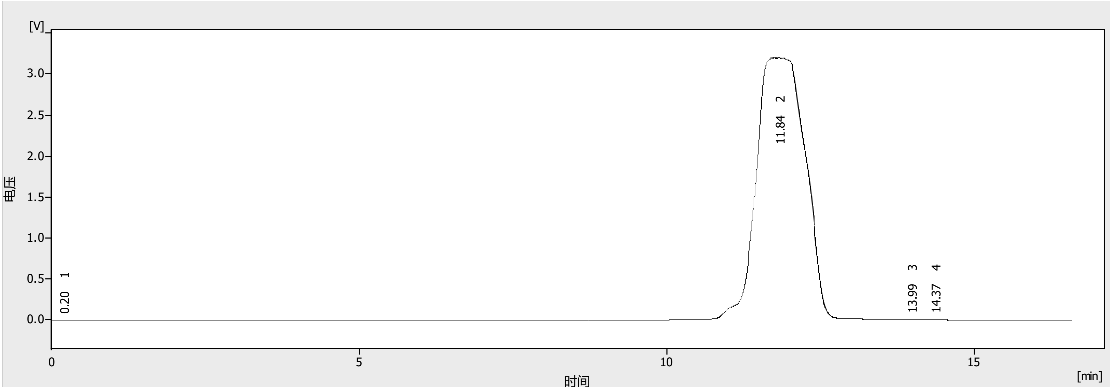

No	Name	Retention/min	Peak area/mV·s	Peak height/mV	Area/%
1	N.A.	0.197	11.007	0.661	0.0
2	N.A.	11.837	179402.674	3223.985	99.3
3	N.A.	13.988	451.642	18.853	0.3
4	N.A.	14.365	722.264	22.195	0.4
5	Total		180587.587	3265.693	100.0

Compound 9h

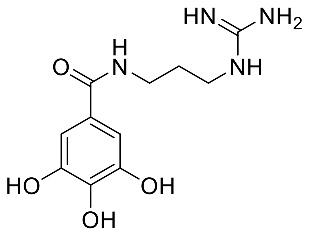

^{1}H-NMR

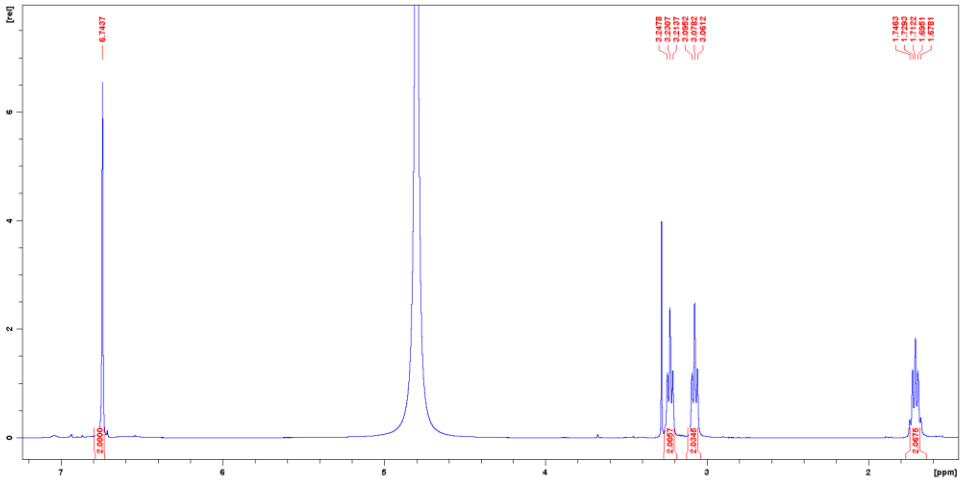

^{13}C-NMR

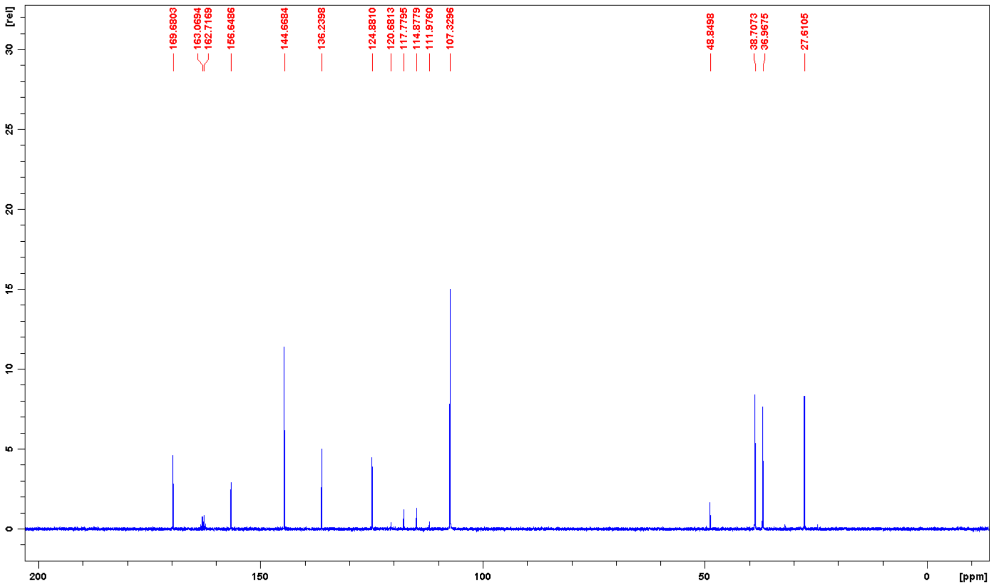

HR- ESI -MS $[M+H]^+$

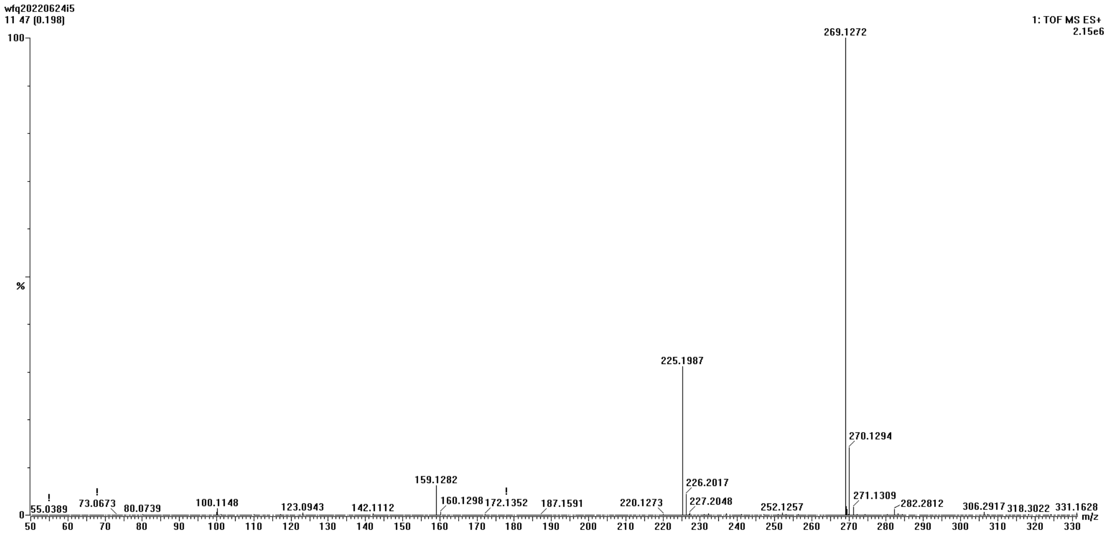

HPLC analysis

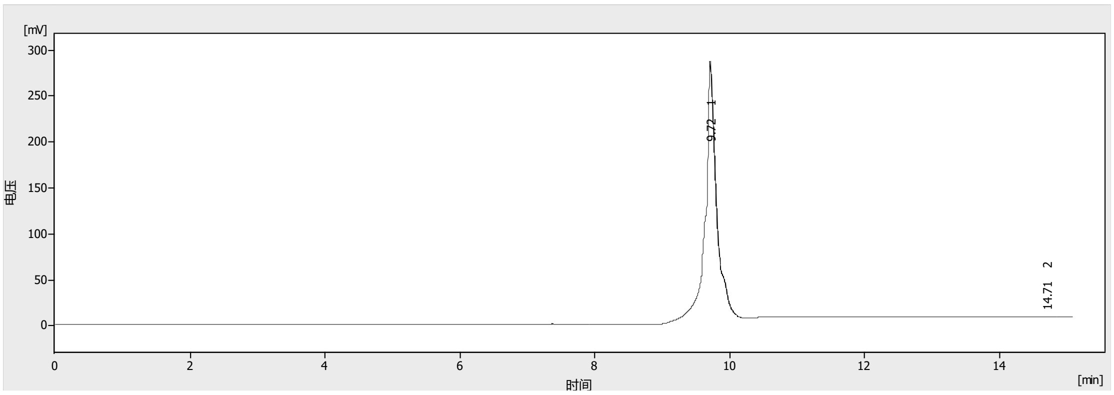

No	Name	Retention/min	Peak area/mV·s	Peak height/mV	Area/%
1	N.A.	9.720	4079.231	286.138	92.8
2	N.A.	14.712	316.621	0.536	7.2
3	Total		4395.853	286.674	100.0

Compound 9i

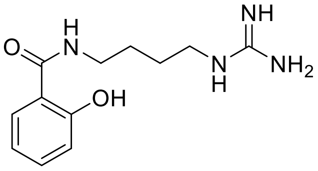

^{1}H-NMR

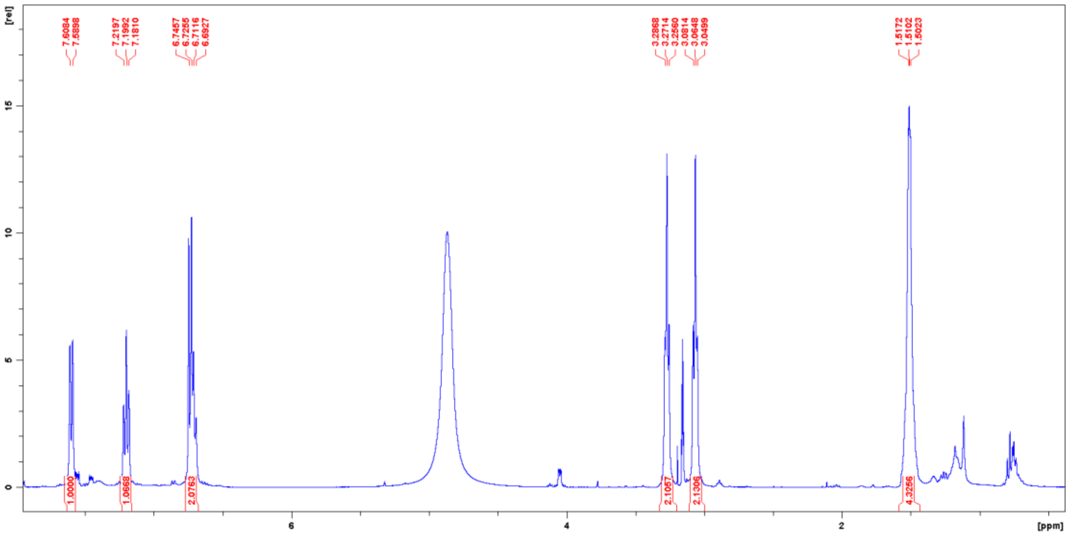

^{13}C-NMR

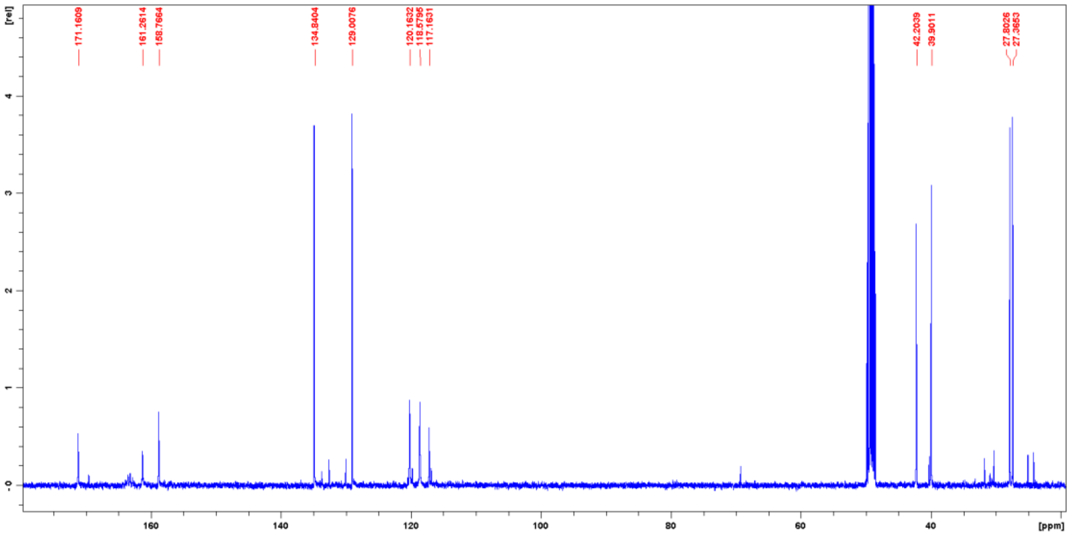

HR- ESI -MS $[M+H]^+$

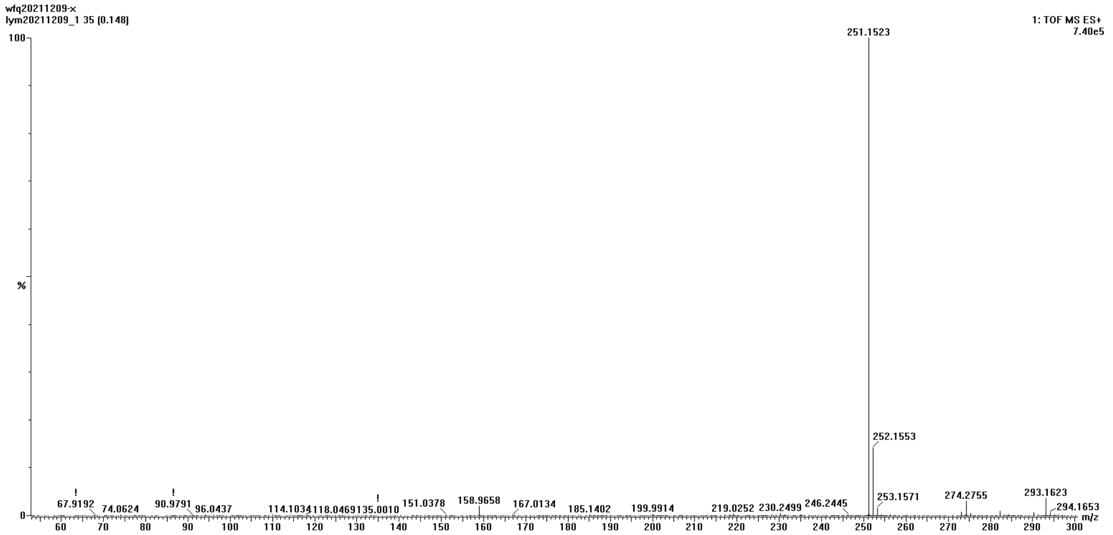

HPLC analysis

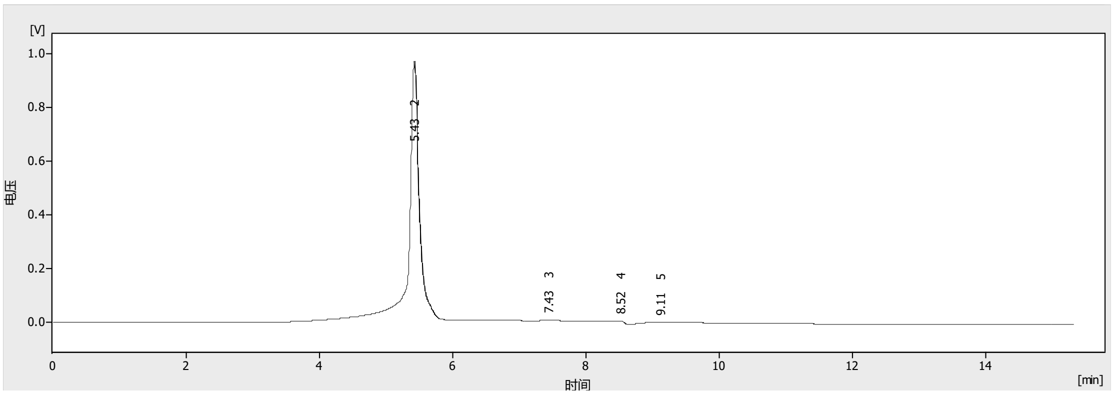

No	Name	Retention/min	Peak area/mV·s	Peak height/mV	Area/%
1	N.A.	6.980	13927.276	1040.842	96.1
2	N.A.	10.687	43.479	4.238	0.3
3	N.A.	11.130	171.493	5.435	1.2
4	N.A.	11.785	347.754	8.886	2.4
5	Total		14490.002	1059.400	100.0

Compound 9j

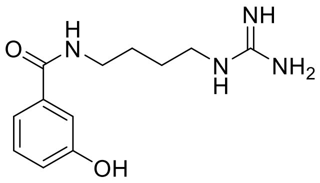

^{1}H-NMR

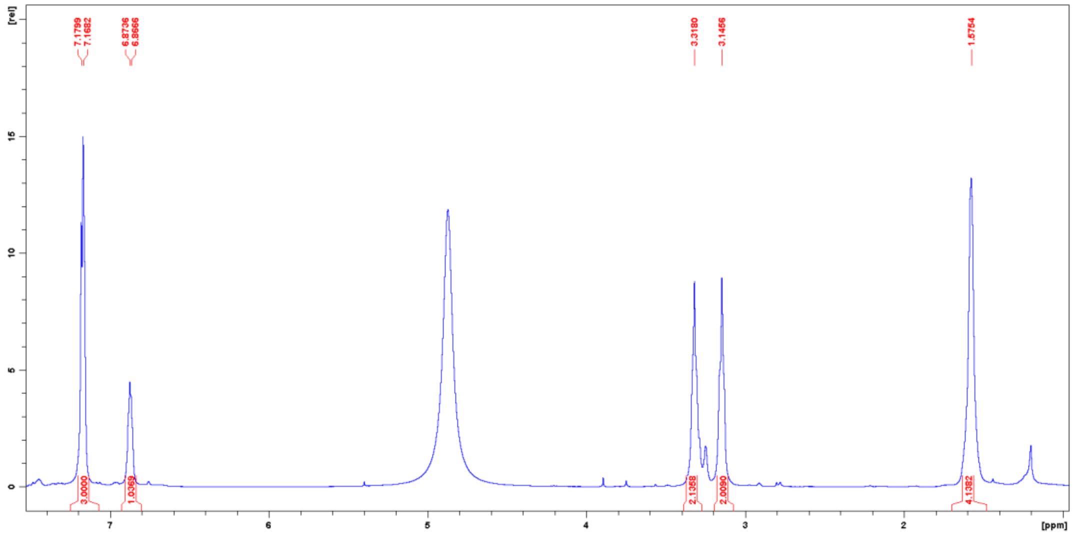

^{13}C-NMR

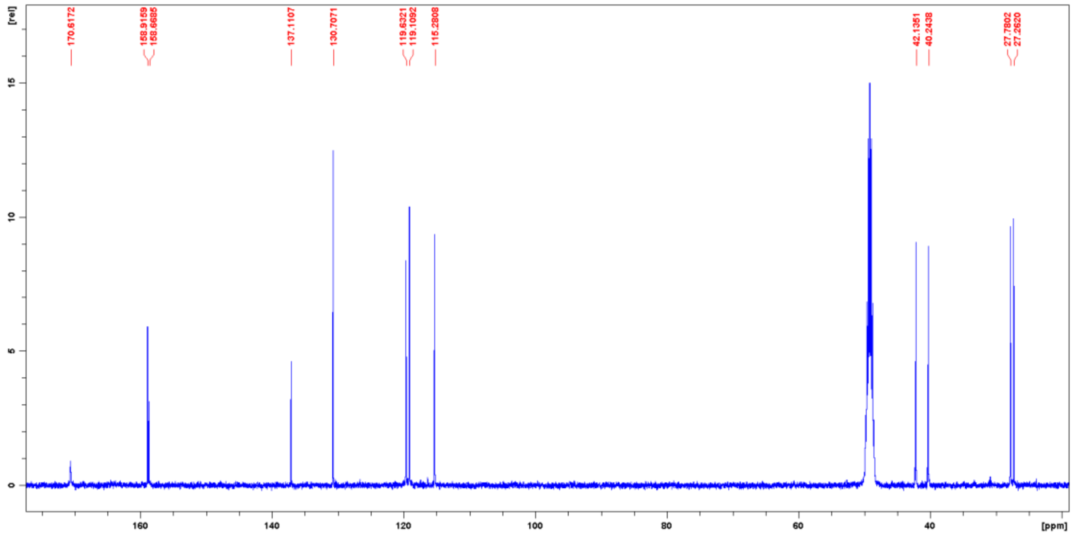

HR- ESI -MS $[M+H]^+$

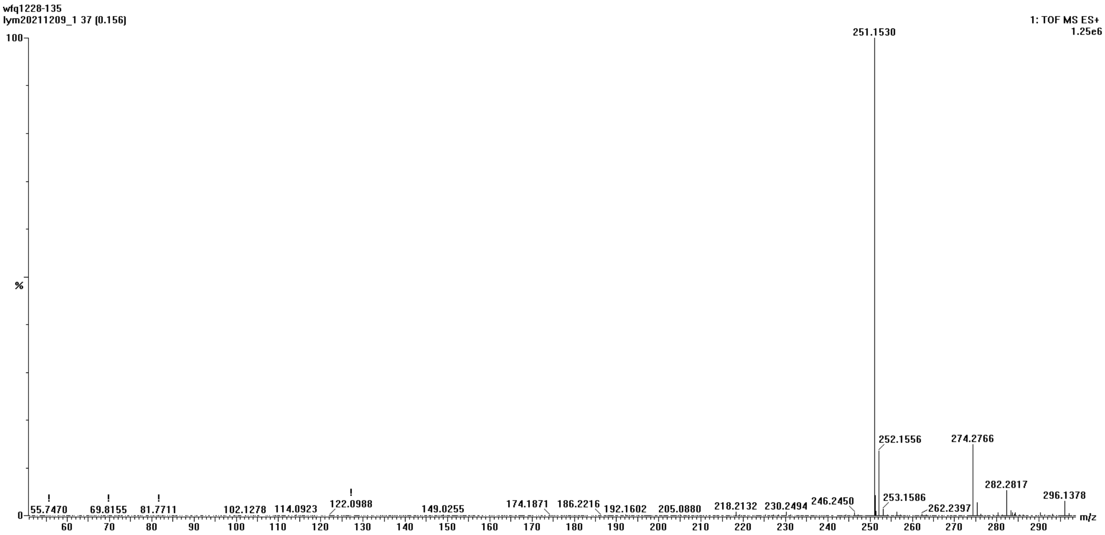

HPLC analysis

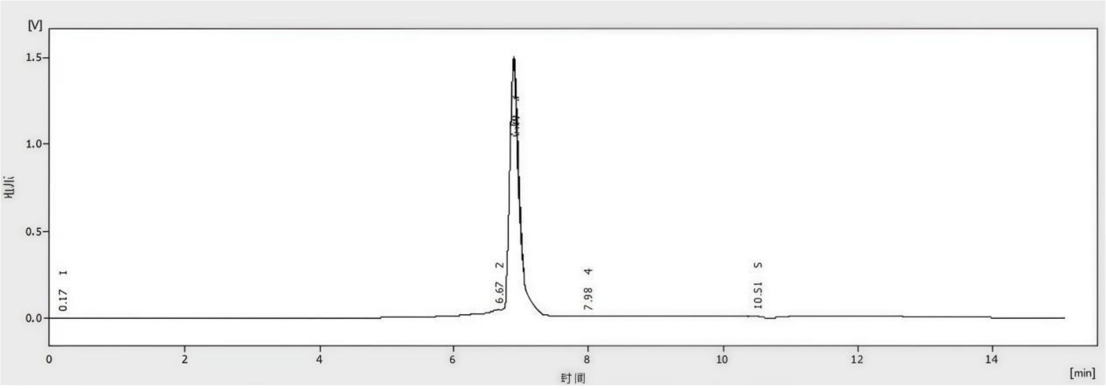

No	Name	Retention/min	Peak area/mV·s	Peak height/mV	Area/%
1	N.A.	0.172	4.335	0.444	0.0
2	N.A.	6.667	506.987	32.333	3.4
3	N.A.	6.892	14465.444	1493.751	96.0
4	N.A.	7.980	33.766	1.446	0.2
5	N.A.	10.512	57.581	5.828	0.4
6	Total		15068.114	1533.802	100.0

Compound 9k

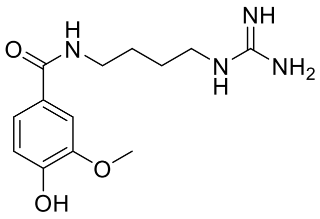

^{1}H-NMR

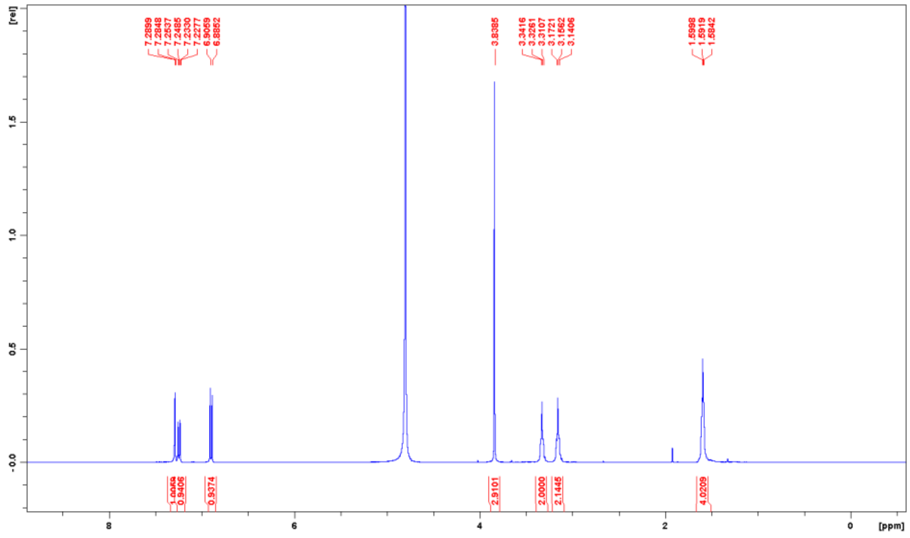

^{13}C-NMR

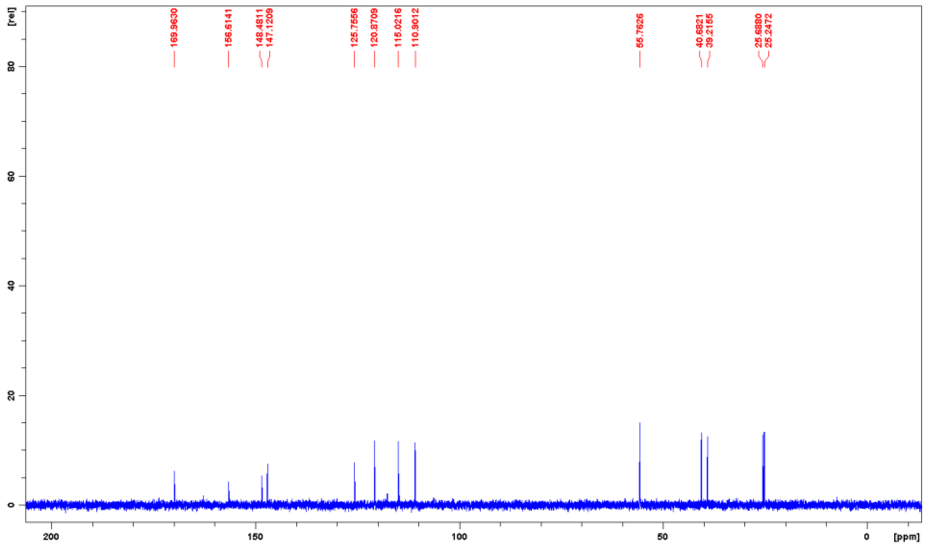

HR- ESI -MS $[M+H]^+$

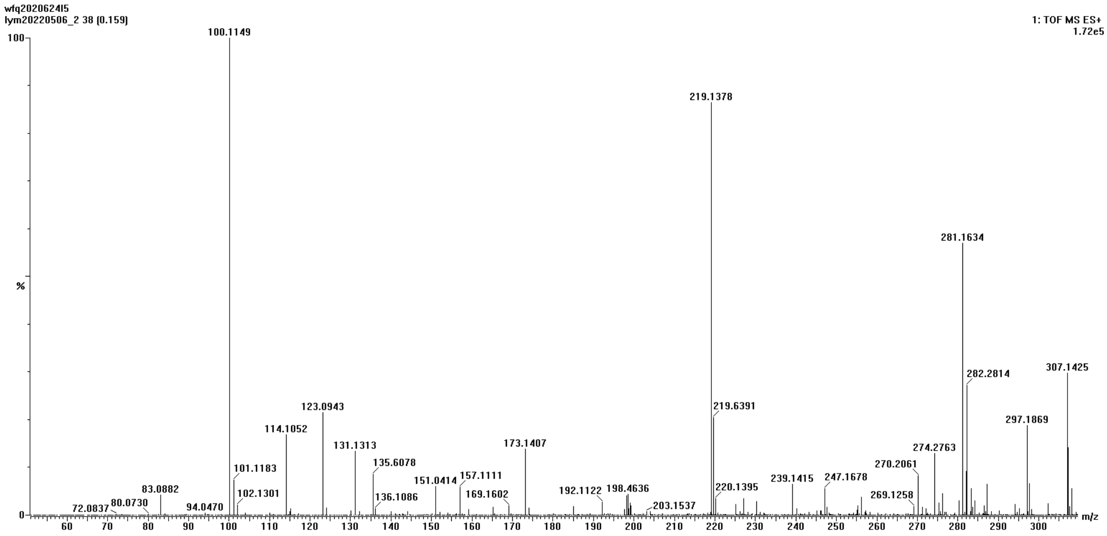

HPLC analysis

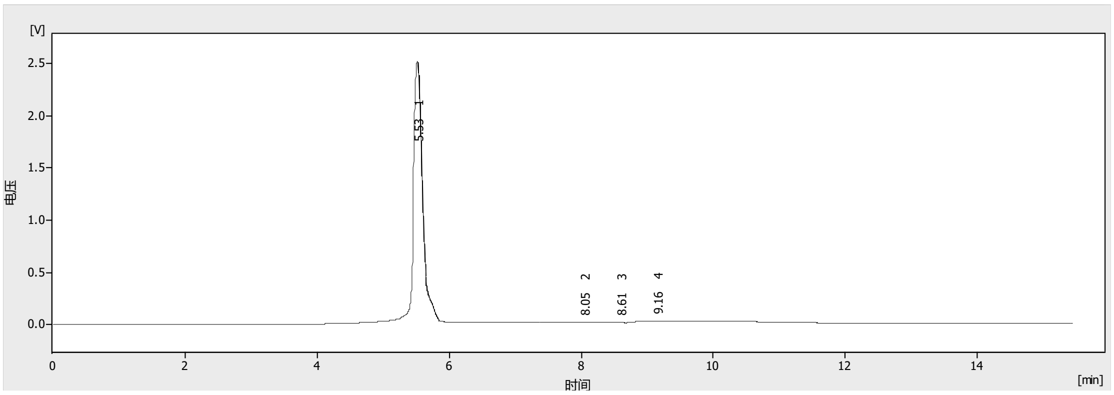

No	Name	Retention/min	Peak area/mV·s	Peak height/mV	Area/%
1	N.A.	5.528	25170.671	2513.449	95.4
2	N.A.	8.055	161.172	6.443	0.6
3	N.A.	8.605	32.708	3.826	0.1
4	N.A.	9.157	1010.068	16.301	3.8
5	Total		26374.620	2540.019	100.0

Compound 9l

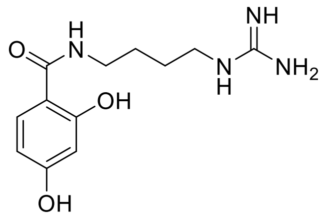

^{1}H-NMR

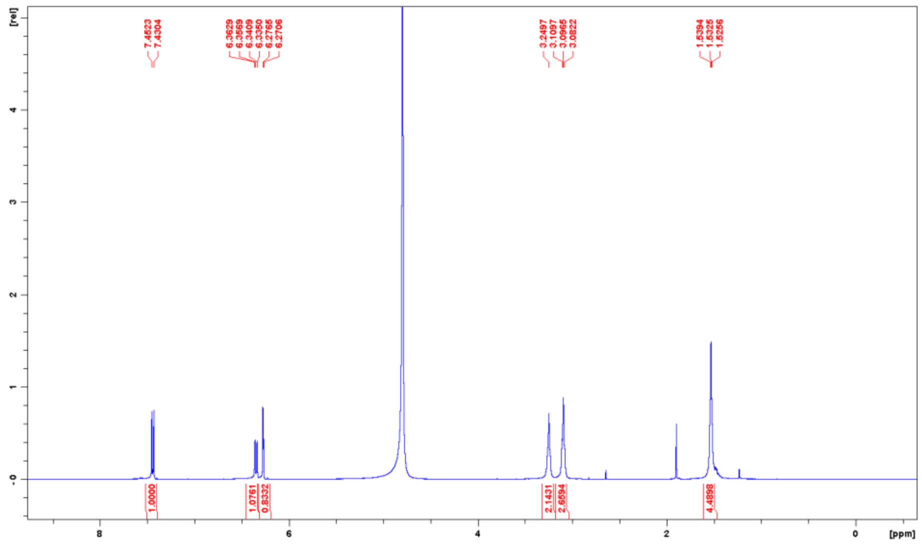

^{13}C-NMR

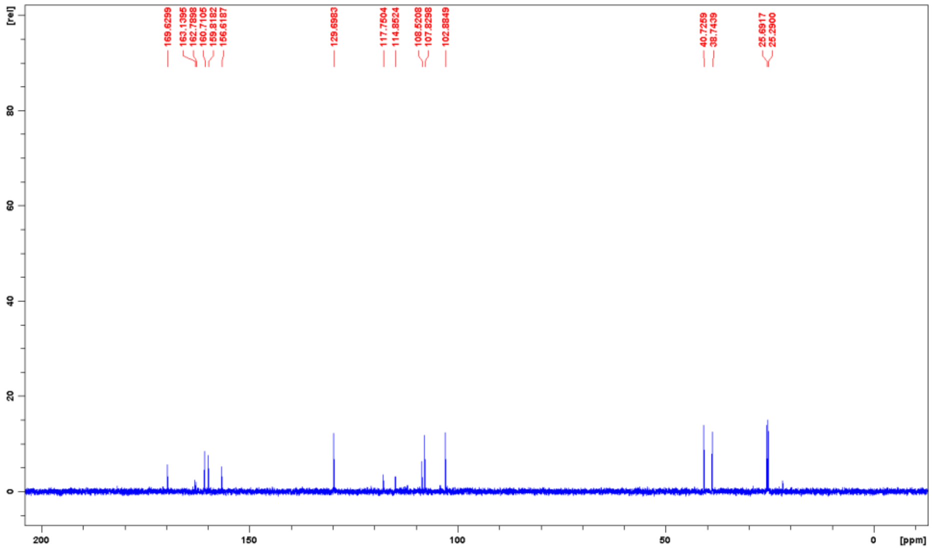

HR- ESI -MS $[M+H]^+$

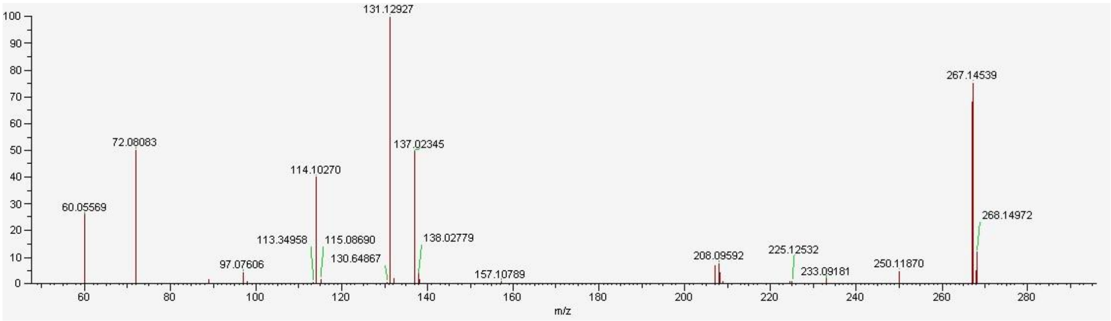

HPLC analysis

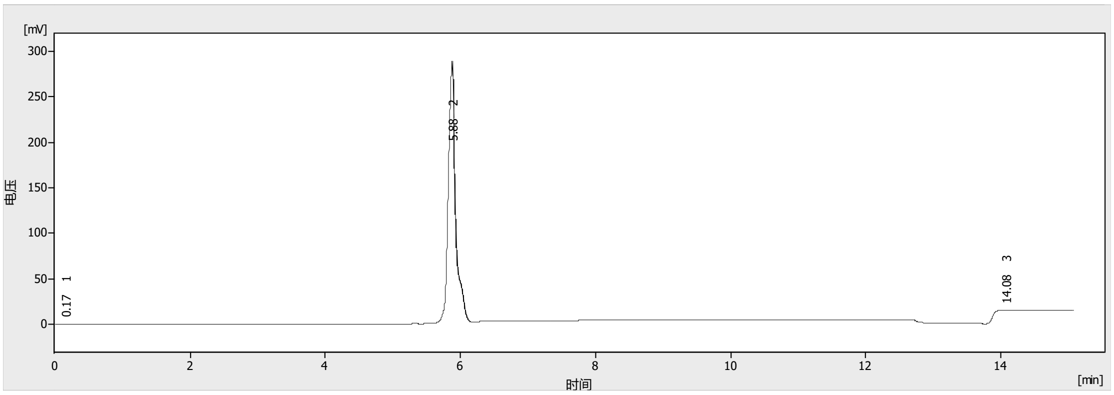

No	Name	Retention/min	Peak area/mV·s	Peak height/mV	Area/%
1	N.A.	0.167	3.922	0.397	0.2
2	N.A.	5.880	2228.877	288.247	95.1
3	N.A.	14.082	110.900	5.001	4.7
4	Total		2343.698	293.645	100.0

Compound 9m

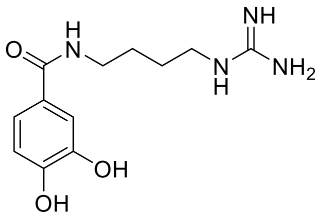

^{1}H-NMR

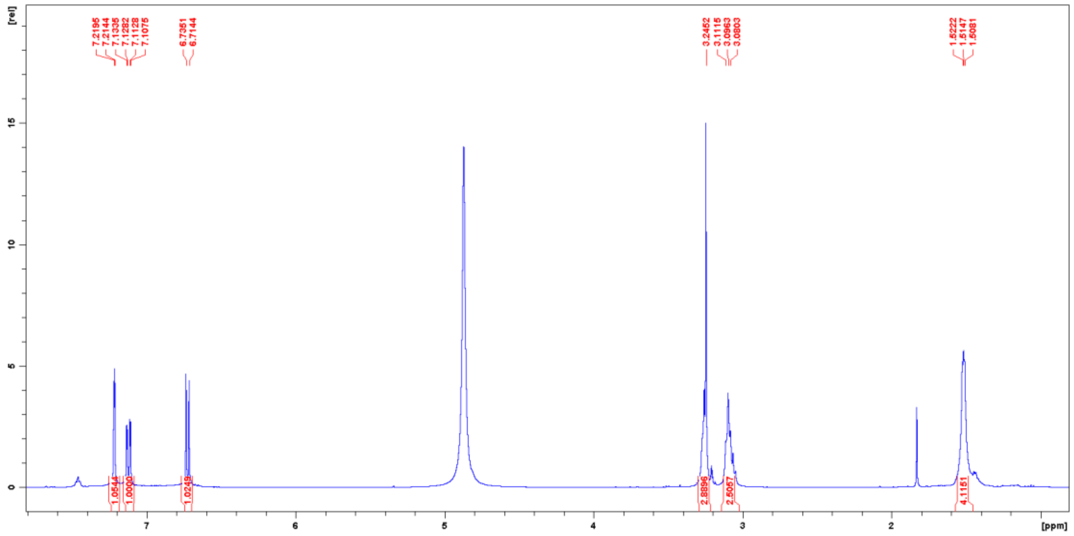

^{13}C-NMR

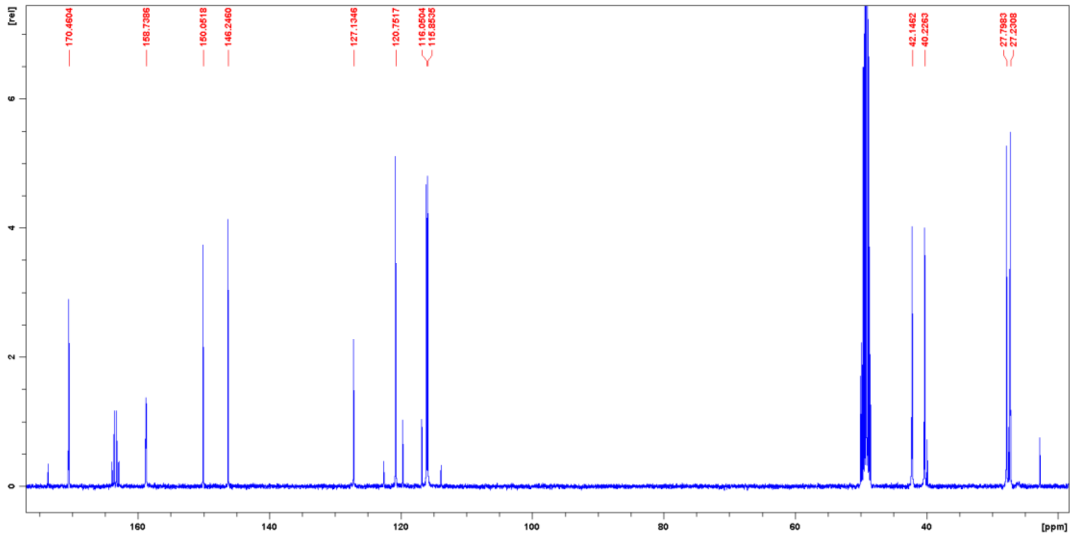

HR- ESI -MS $[M+H]^{+}$

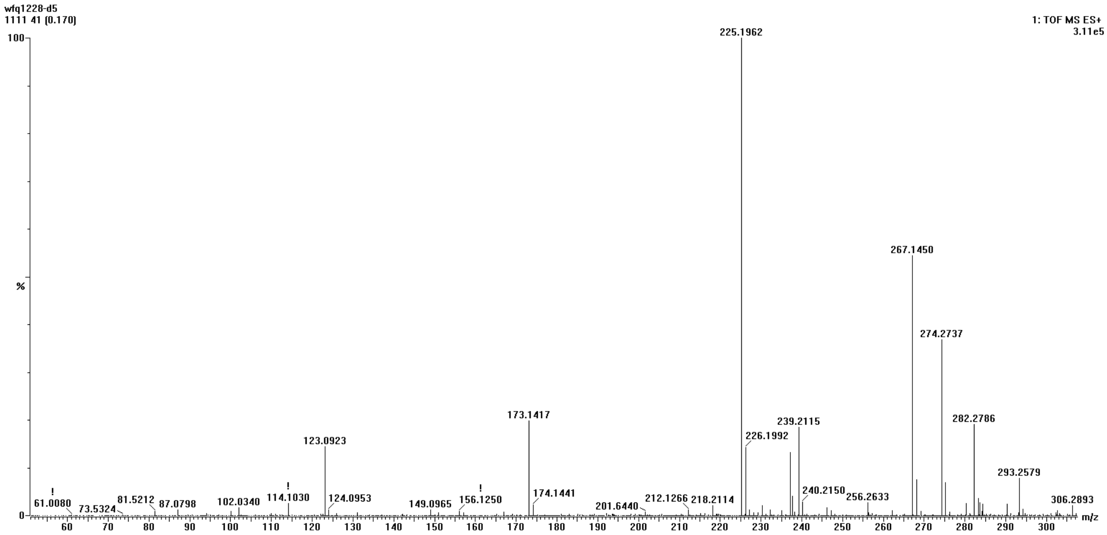

HPLC analysis

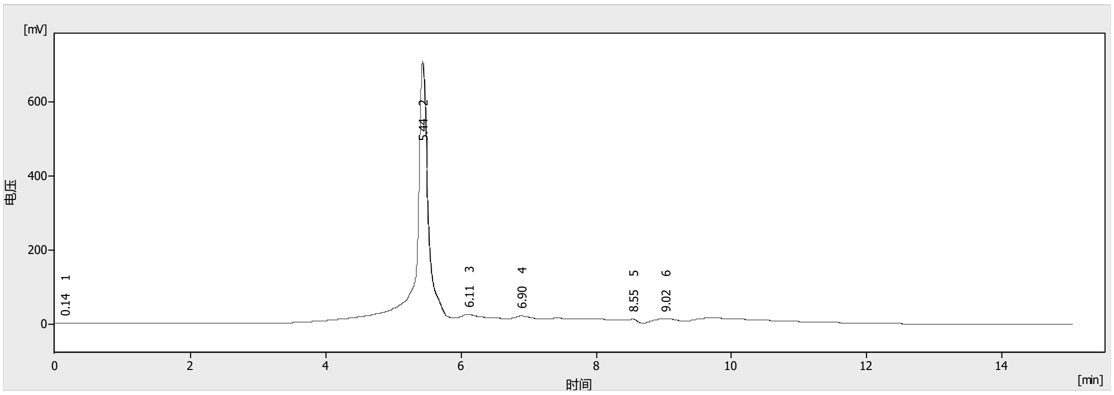

No	Name	Retention/min	Peak area/mV·s	Peak height/mV	Area/%
1	N.A.	0.143	10.106	0.885	0.1
2	N.A.	5.437	8192.263	697.269	95.2
3	N.A.	6.110	68.688	7.070	0.8
4	N.A.	6.898	112.559	7.892	1.3
5	N.A.	8.555	67.995	7.268	0.8
6	N.A.	9.023	155.348	7.832	1.8
7	Total		8606.958	728.216	100.0

Compound 9n

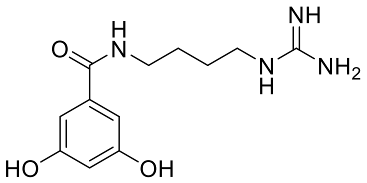

^{1}H-NMR

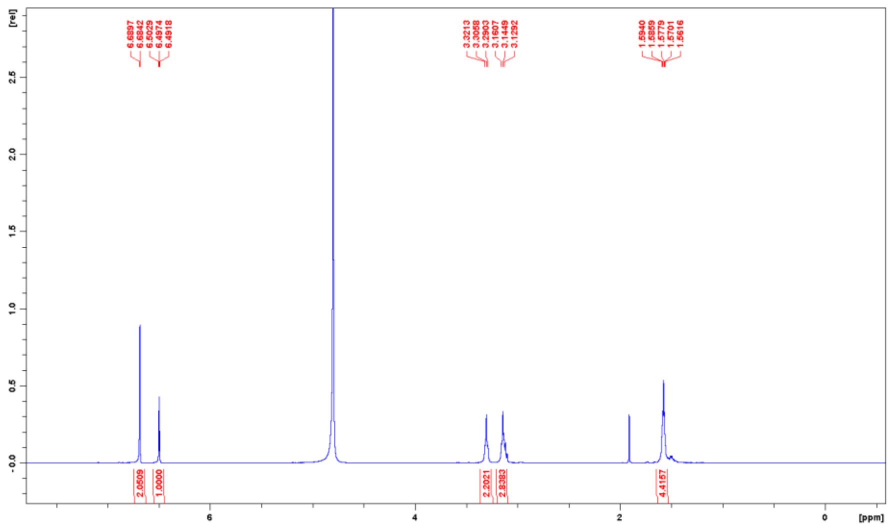

^{13}C-NMR

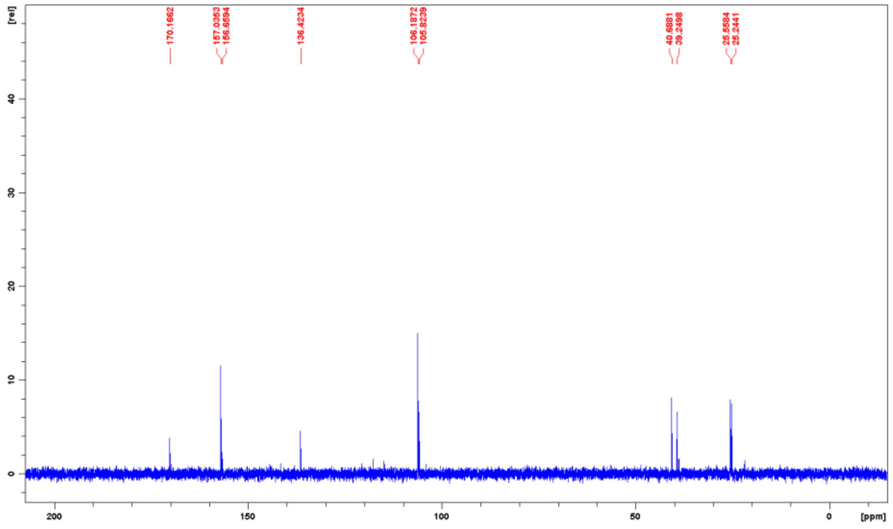

HR- ESI -MS [M+H]$^+$

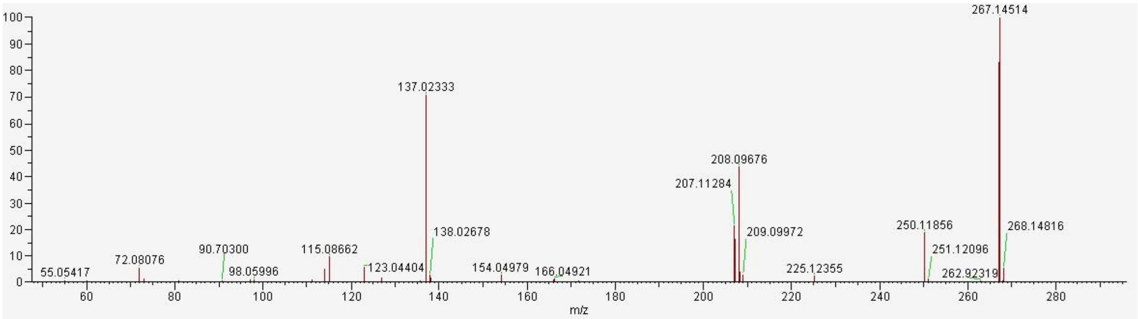

HPLC analysis

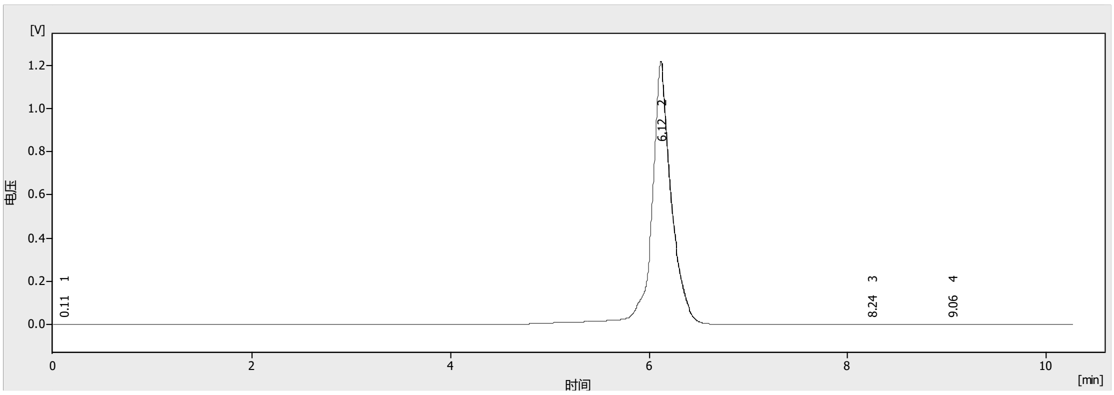

No	Name	Retention/min	Peak area/mV·s	Peak height/mV	Area/%
1	N.A.	0.105	8.448	1.395	0.0
2	N.A.	6.122	17056.786	1224.419	98.9
3	N.A.	8.245	137.598	3.614	0.8
4	N.A.	9.058	43.983	1.509	0.3
5	Total		17246.815	1230.937	100.0

Compound 9o

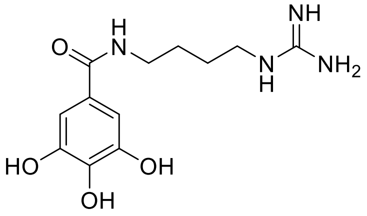

^{1}H-NMR

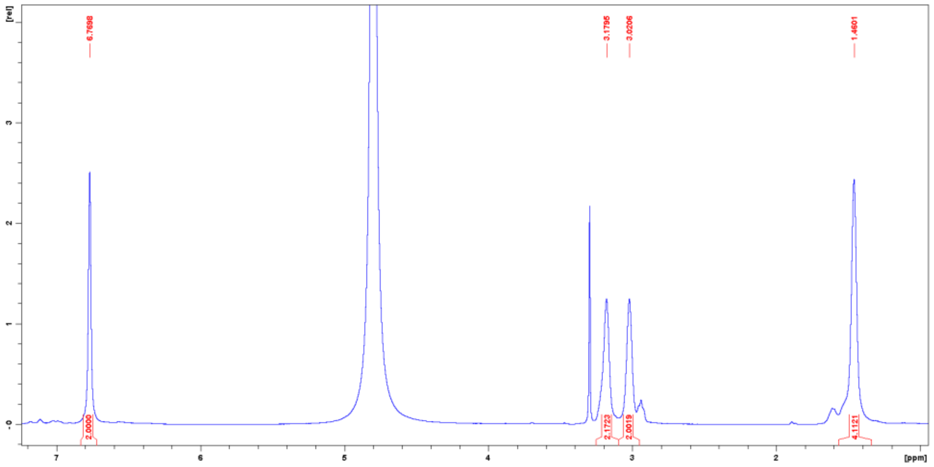

^{13}C-NMR

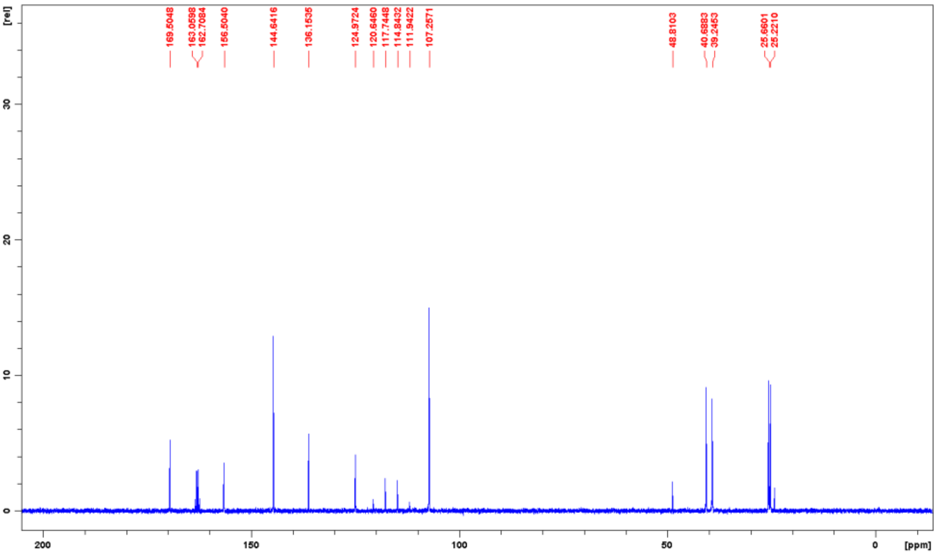

HR- ESI -MS $[M+H]^+$

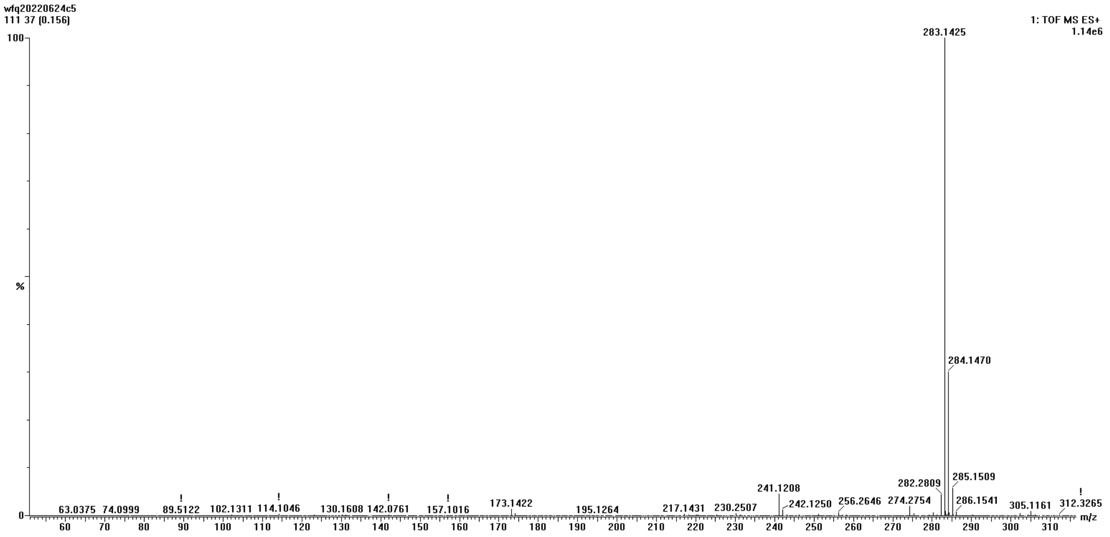

HPLC analysis

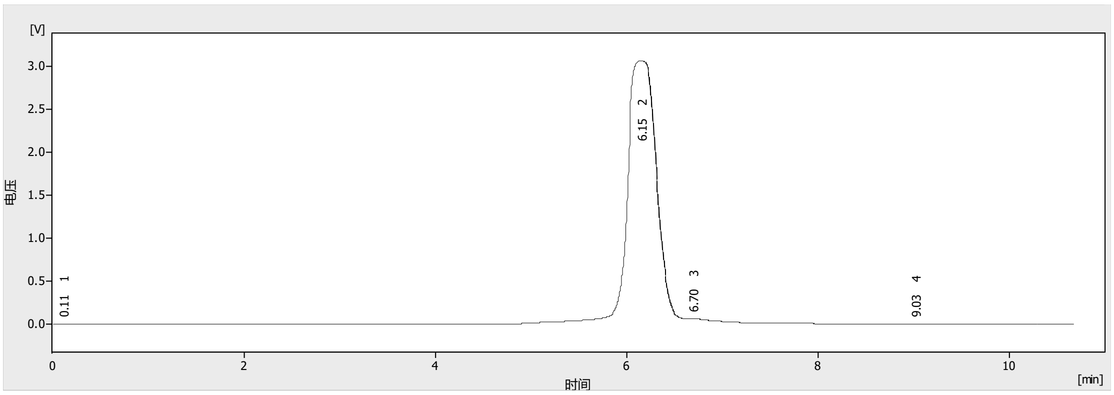

No	Name	Retention/min	Peak area/mV·s	Peak height/mV	Area/%
1	N.A.	0.110	6.968	1.245	0.0
2	N.A.	6.153	65461.867	3071.157	96.0
3	N.A.	6.697	2495.881	67.834	3.7
4	N.A.	9.028	242.157	4.892	0.4
5	Total		68206.873	3145.127	100.0

Compound 9p

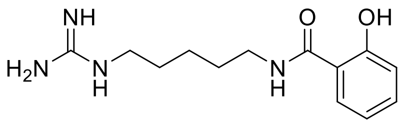

^{1}H-NMR

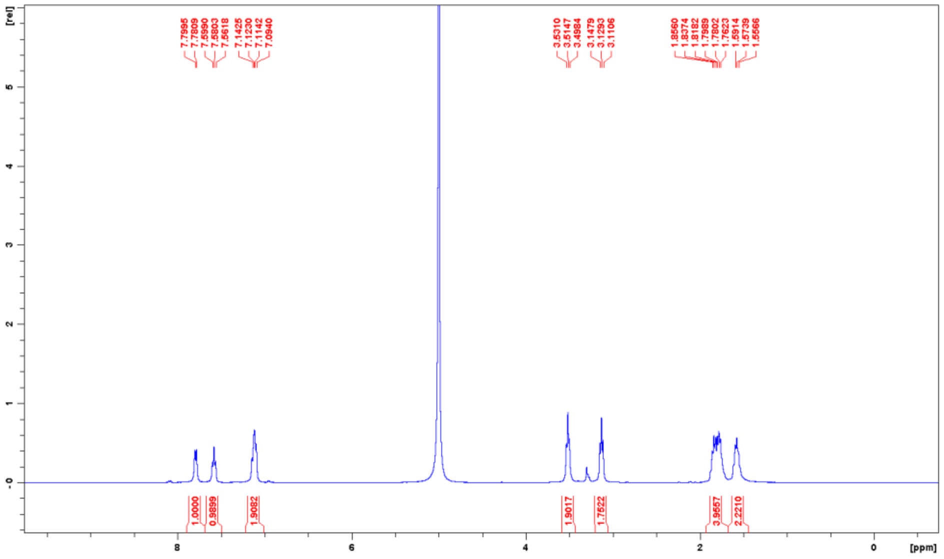

^{13}C-NMR

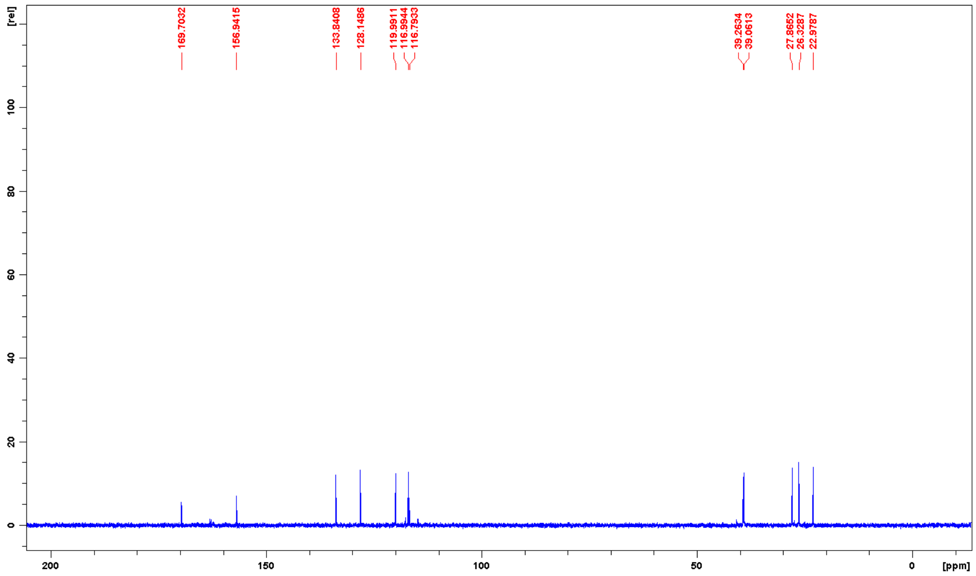

HR- ESI -MS $[M+H]^+$

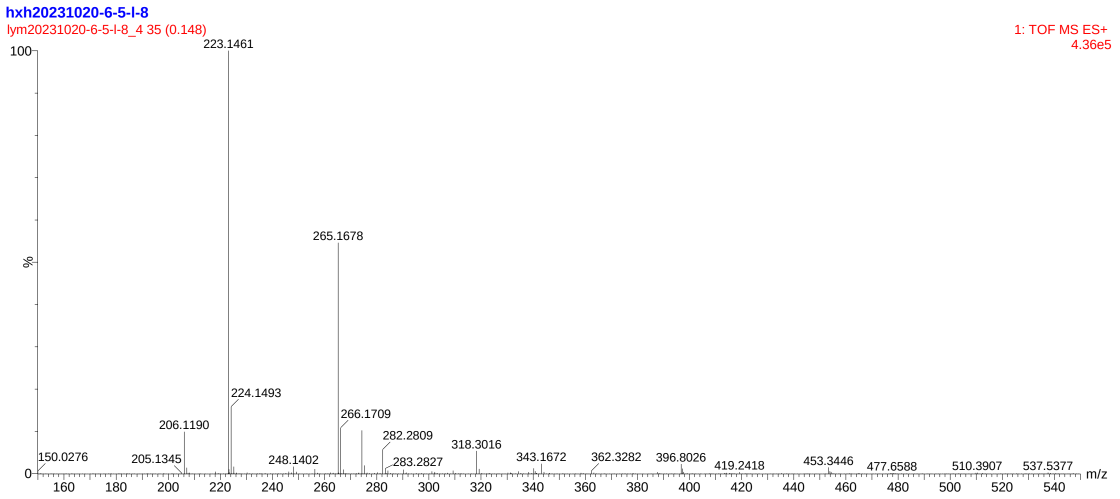

HPLC analysis

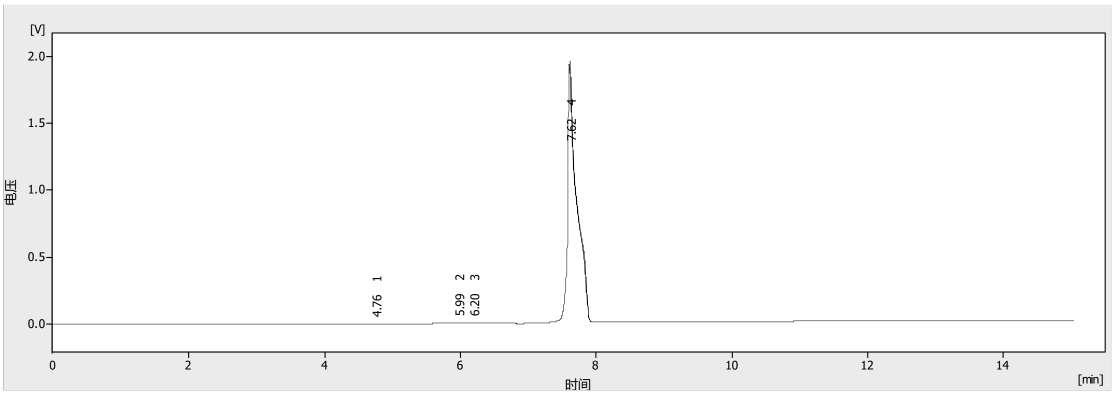

No	Name	Retention/min	Peak area/mV·s	Peak height/mV	Area/%
1	N.A.	4.760	18.742	2.256	0.1
2	N.A.	5.990	216.004	6.048	1.2
3	N.A.	6.203	215.234	6.806	1.2
4	N.A.	7.623	17999.548	1957.171	97.6
5	Total		18449.528	1972.281	100.0

Compound 9q

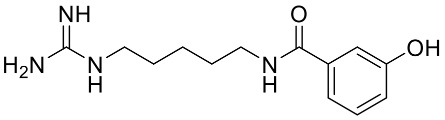

^{1}H-NMR

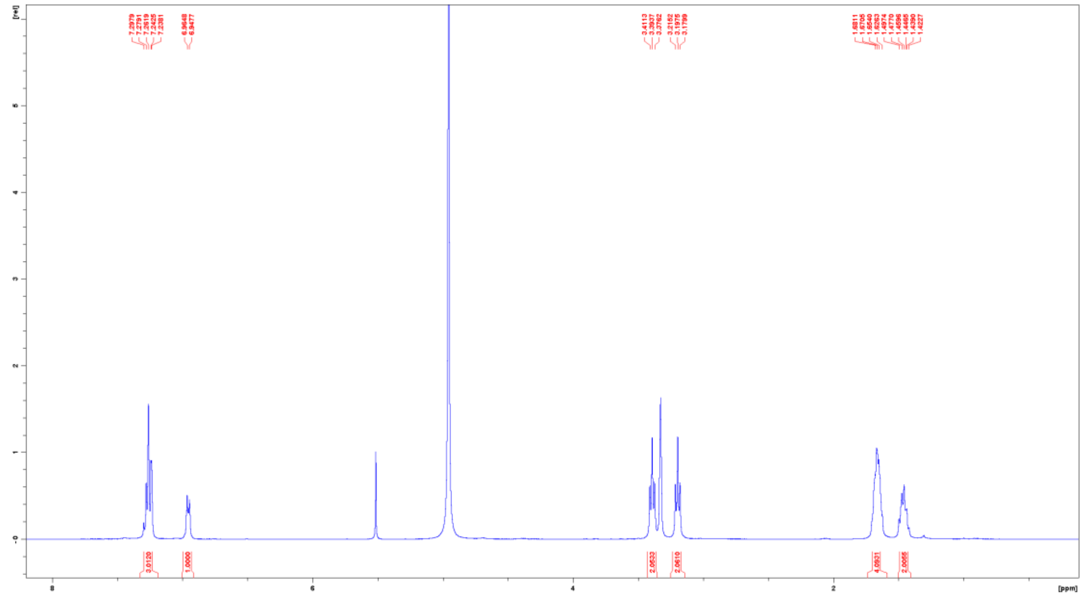

^{13}C-NMR

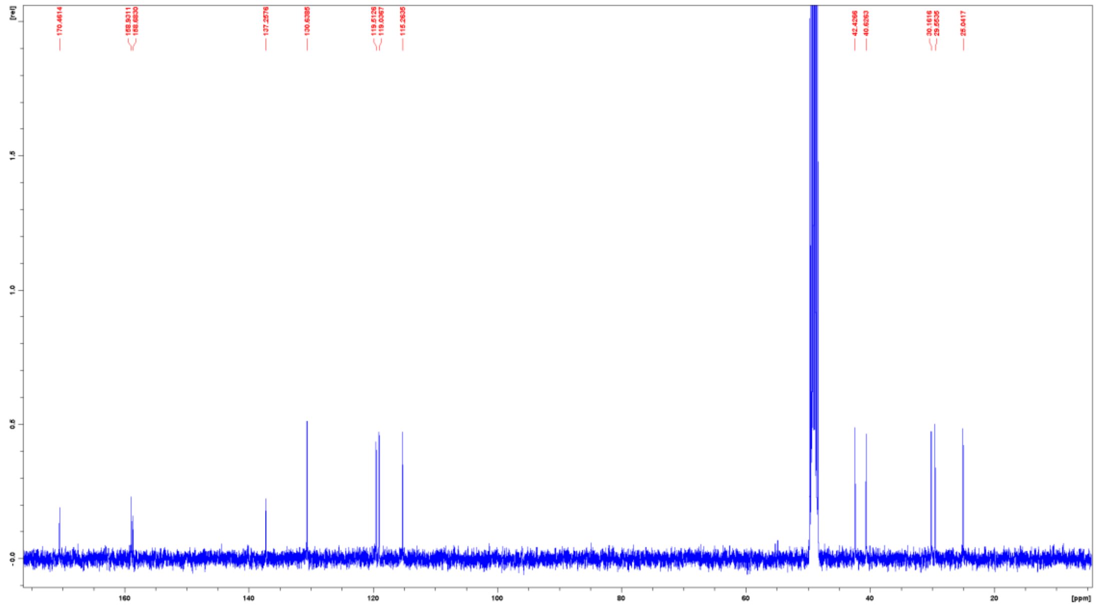

HR- ESI -MS [M+H]$^+$

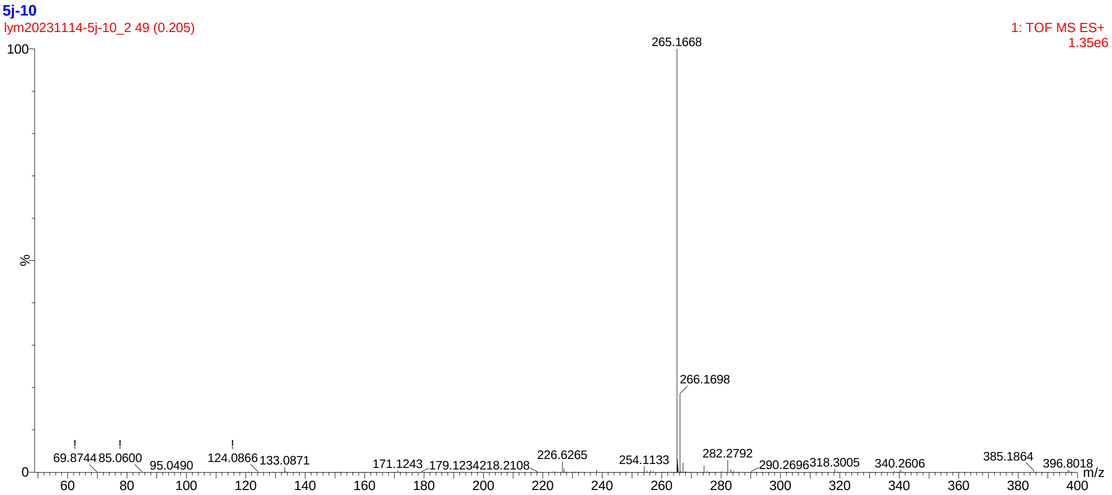

HPLC analysis

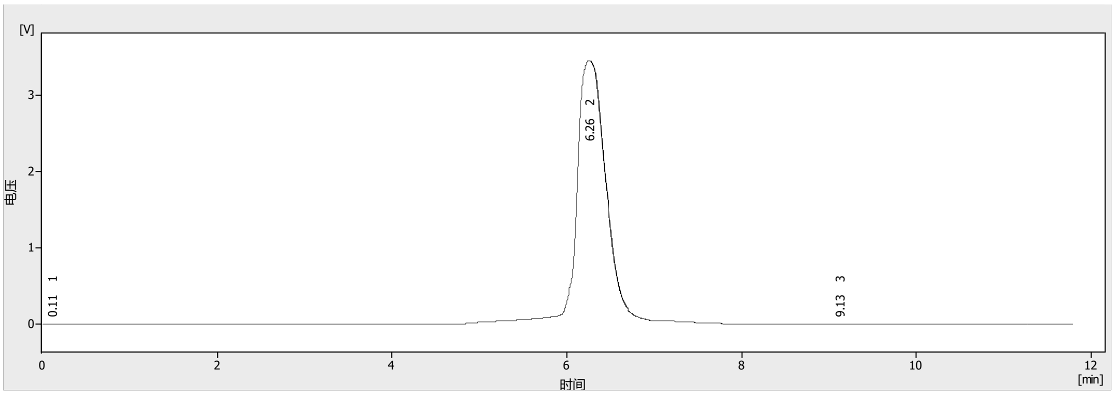

No	Name	Retention/min	Peak area/mV·s	Peak height/mV	Area/%
1	N.A.	0.108	6.480	1.103	0.0
2	N.A.	6.258	83221.075	3450.909	99.9
3	N.A.	9.127	79.285	2.479	0.1
4	Total		83306.839	3454.492	100.0

Compound 9r

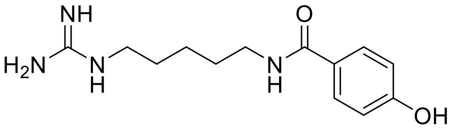

^{1}H-NMR

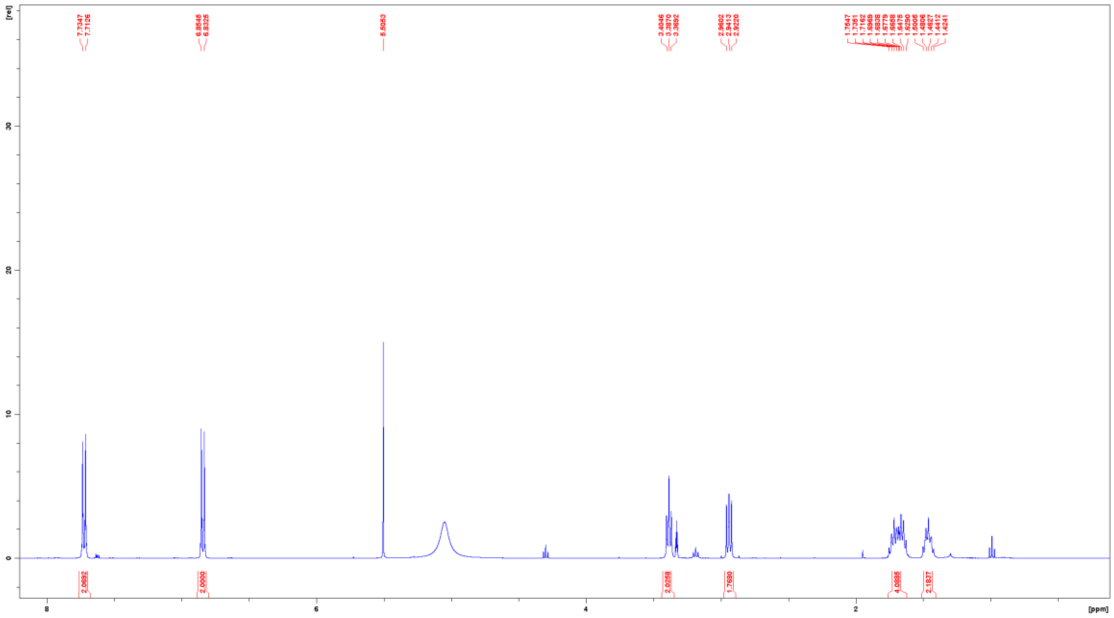

^{13}C-NMR

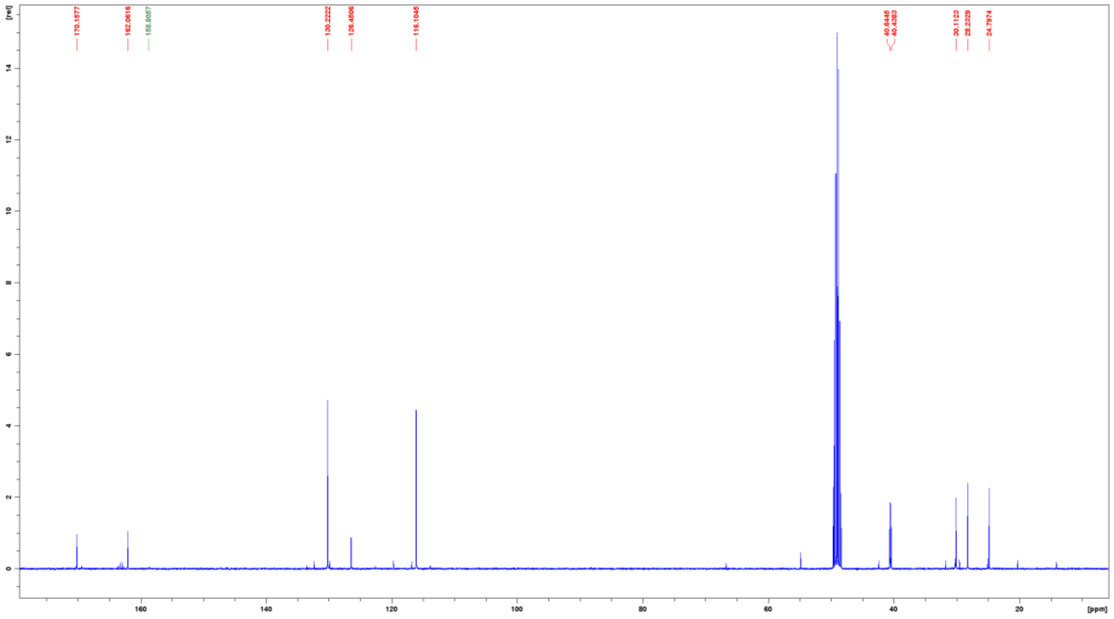

HR- ESI -MS $[M+H]^+$

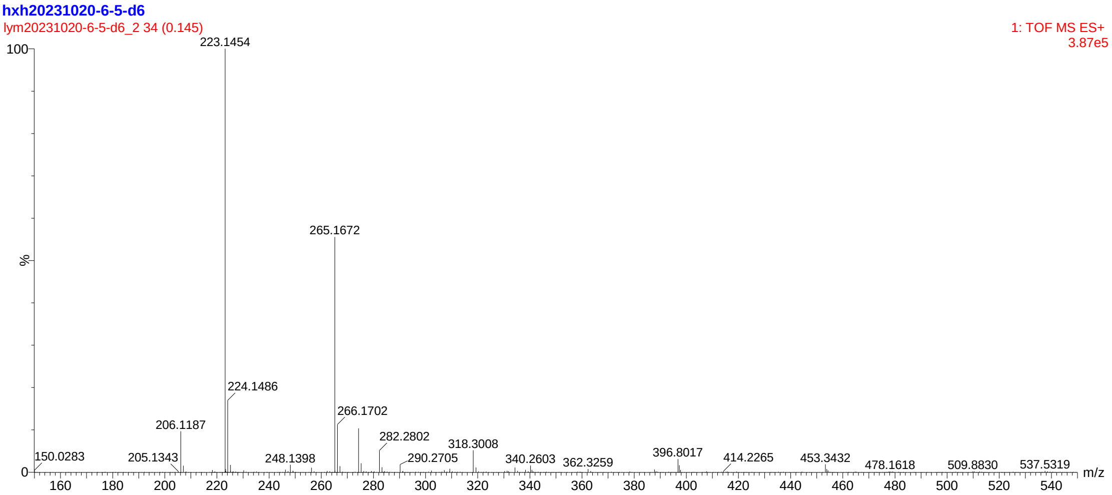

HPLC analysis

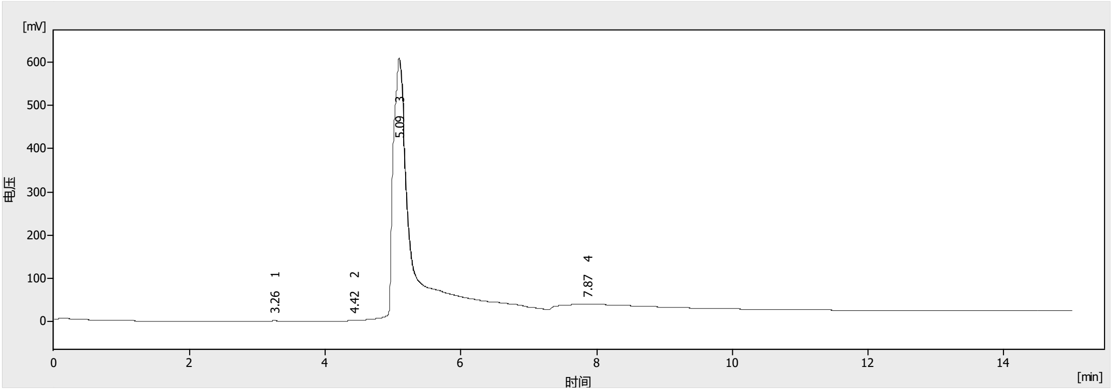

No	Name	Retention/min	Peak area/mV·s	Peak height/mV	Area/%
1	N.A.	3.257	45.318	1.611	0.4
2	N.A.	4.425	6.830	1.119	0.1
3	N.A.	5.090	11854.776	603.766	96.7
4	N.A.	7.867	350.241	6.968	2.9
5	Total		12257.165	613.464	100.0

Compound 9s

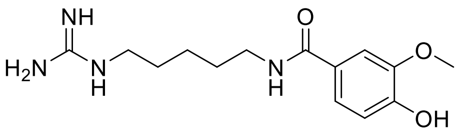

^{1}H-NMR

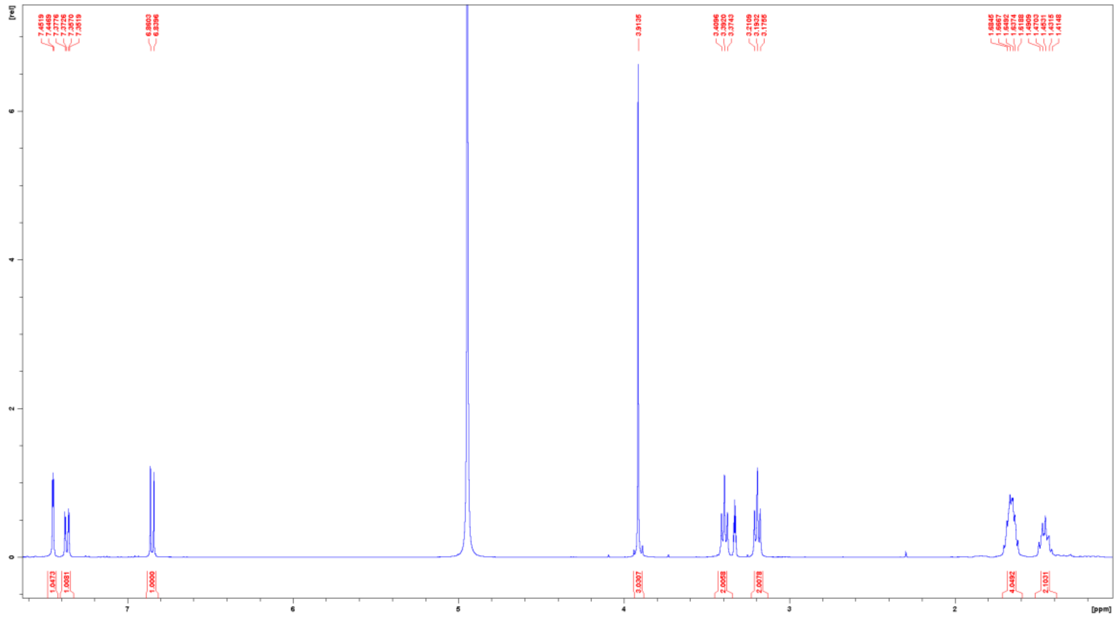

^{13}C-NMR

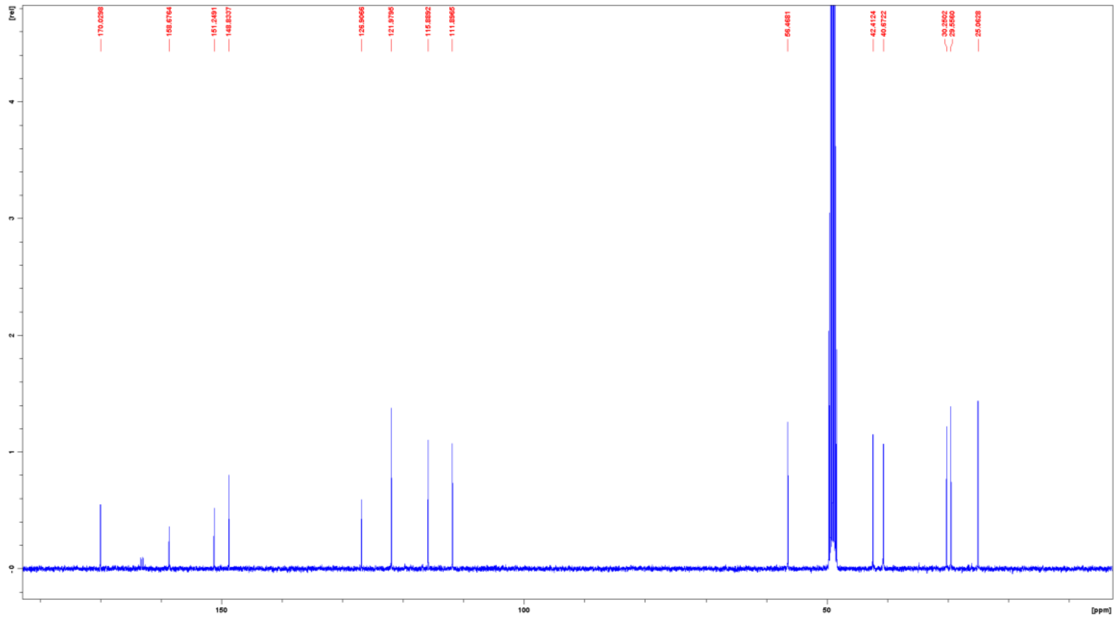

HR- ESI -MS $[M+H]^+$

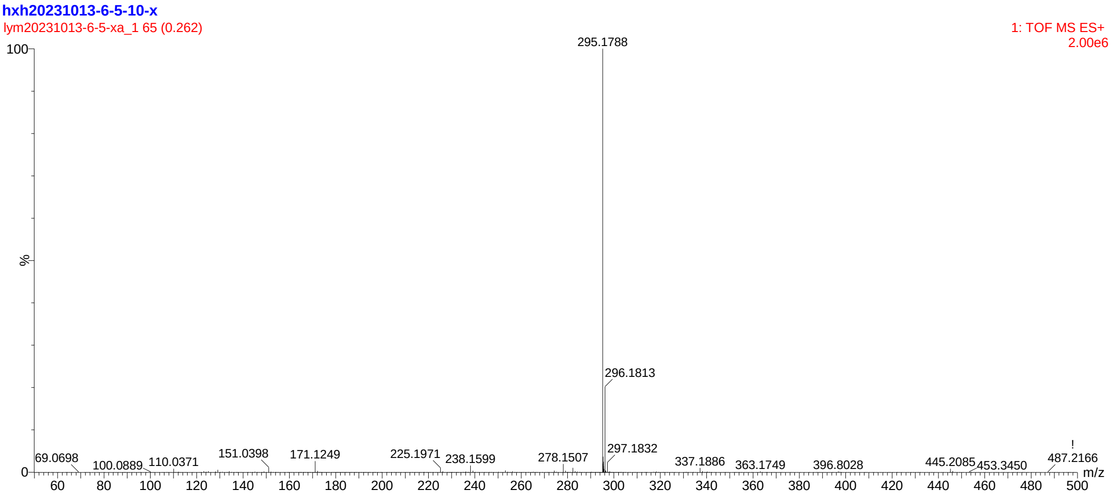

HPLC analysis

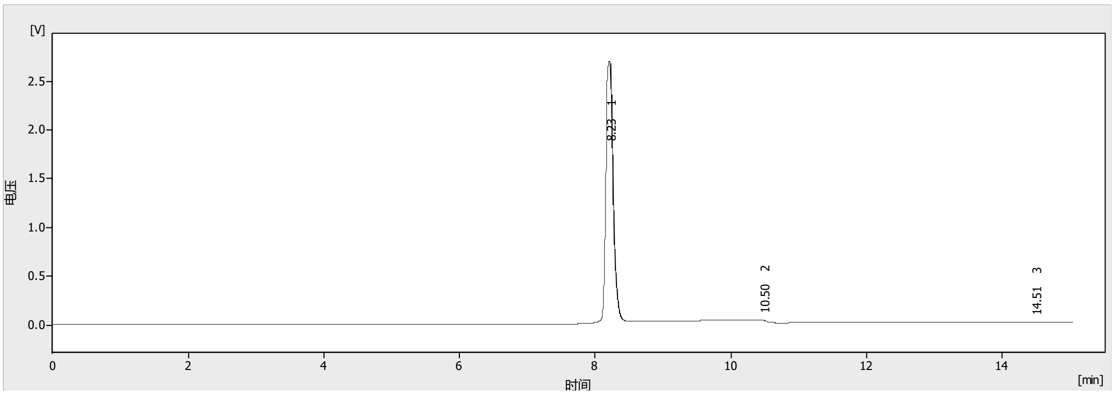

No	Name	Retention/min	Peak area/mV·s	Peak height/mV	Area/%
1	N.A.	8.227	20831.929	2680.884	95.4
2	N.A.	10.498	722.103	22.149	3.3
3	N.A.	14.513	288.838	0.303	1.3
4	Total		21842.870	2703.335	100.0

Compound 9t

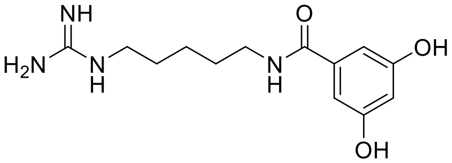

^{1}H-NMR

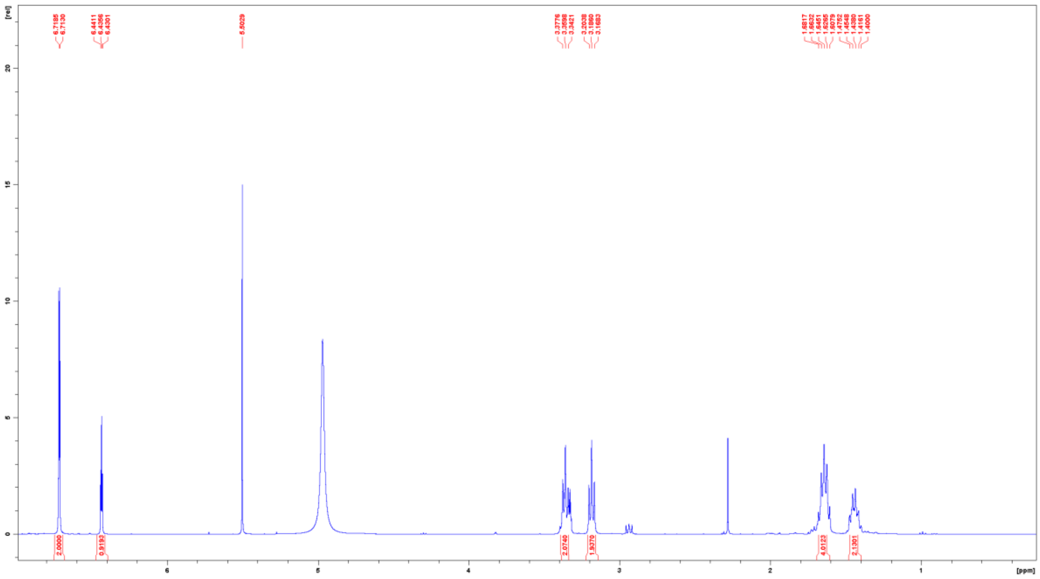

^{13}C-NMR

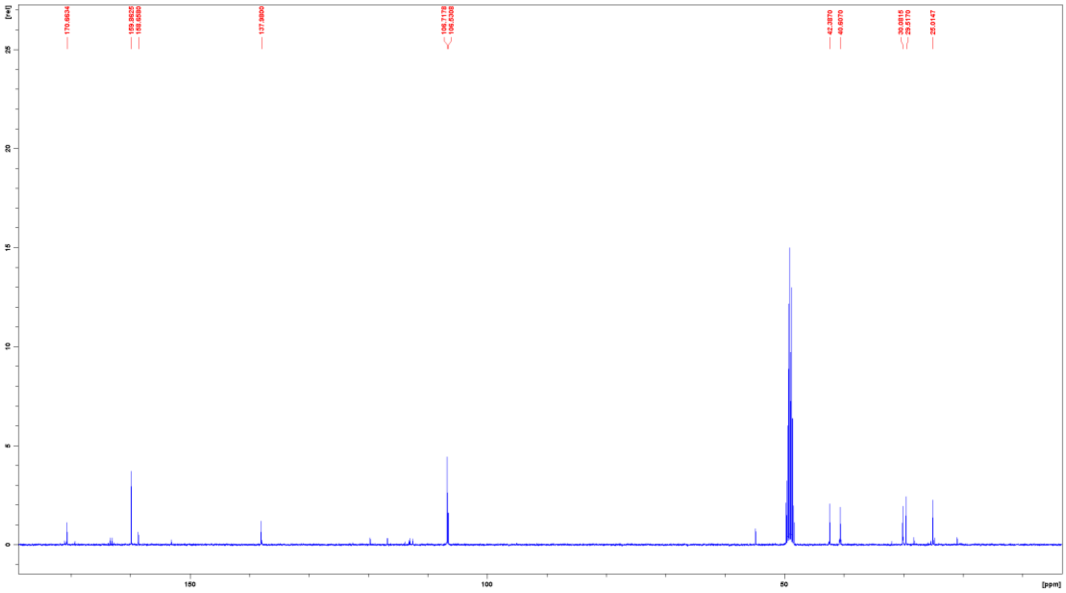

HR- ESI -MS $[M+H]^+$

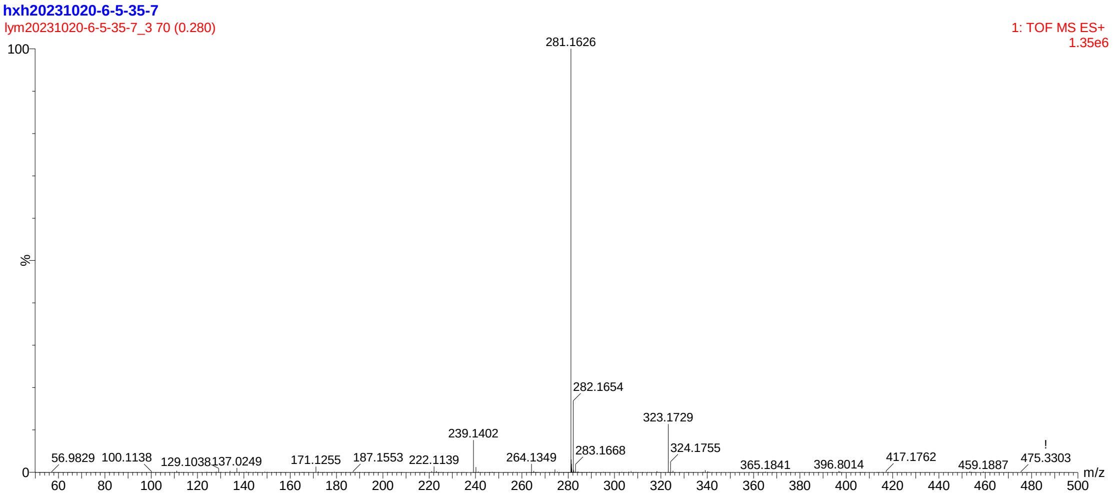

HPLC analysis

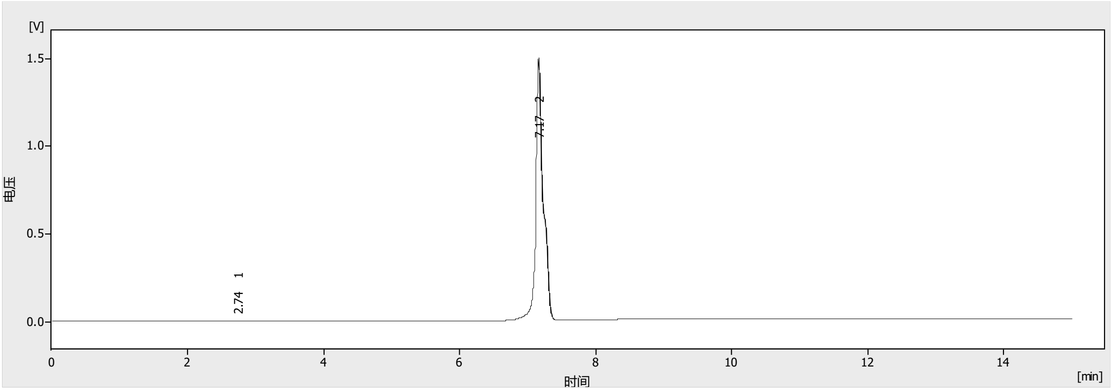

No	Name	Retention/min	Peak area/mV·s	Peak height/mV	Area/%
1	N.A.	2.742	25.523	0.847	0.2
2	N.A.	7.172	11428.093	1497.188	99.8
3	Total		11453.616	1498.035	100.0

Compound 9u

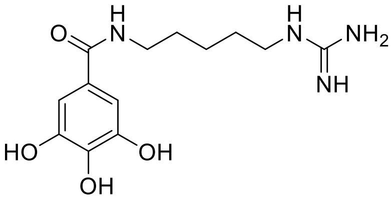

^{1}H-NMR

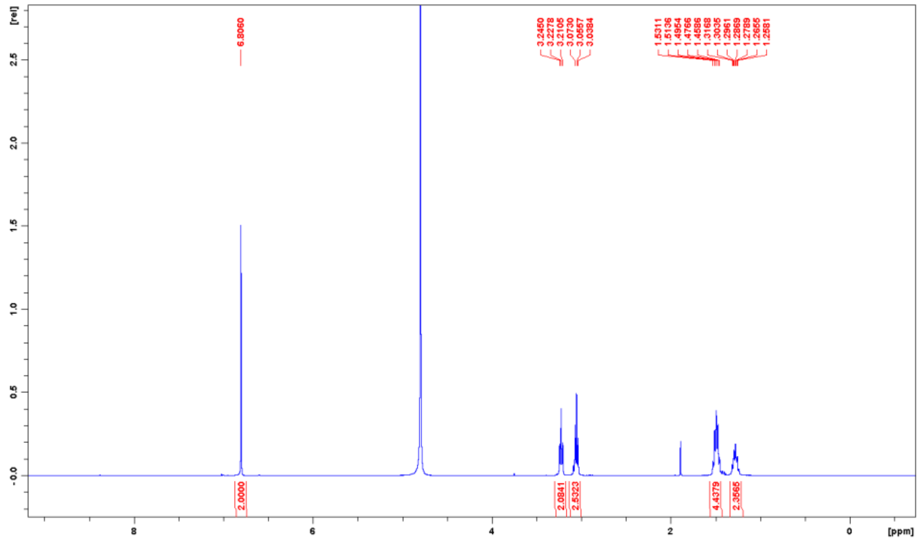

^{13}C-NMR

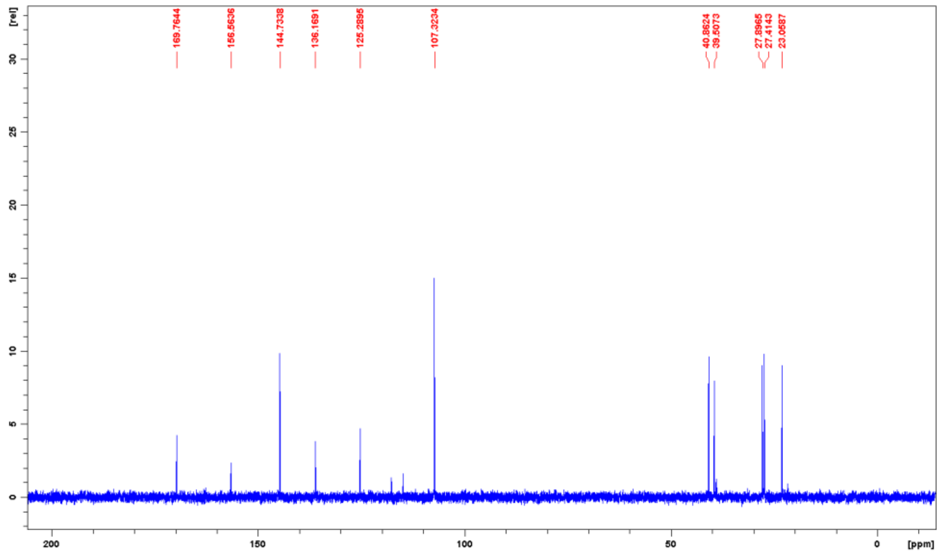

HR- ESI -MS $[M+H]^+$

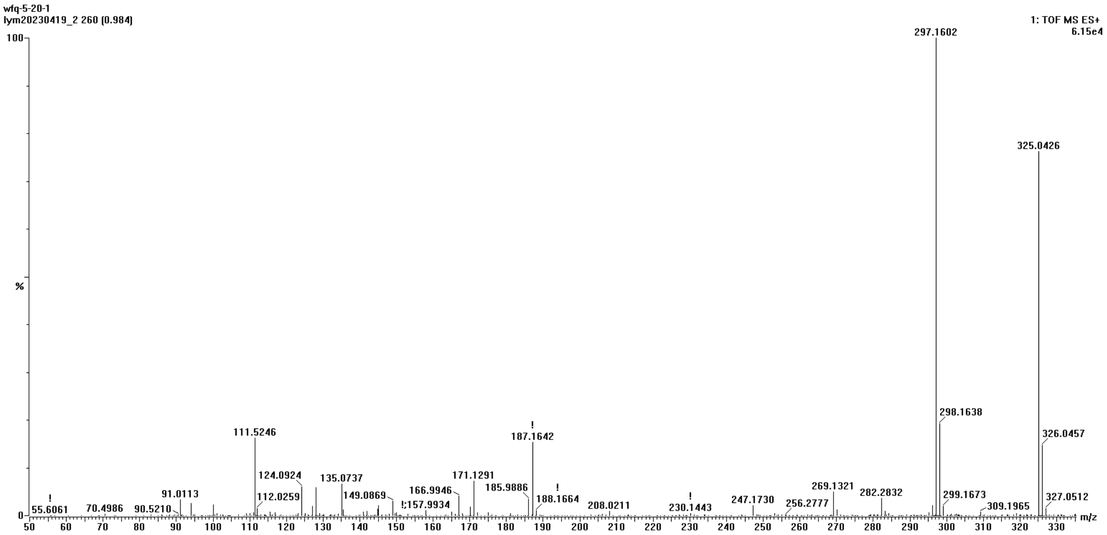

HPLC analysis

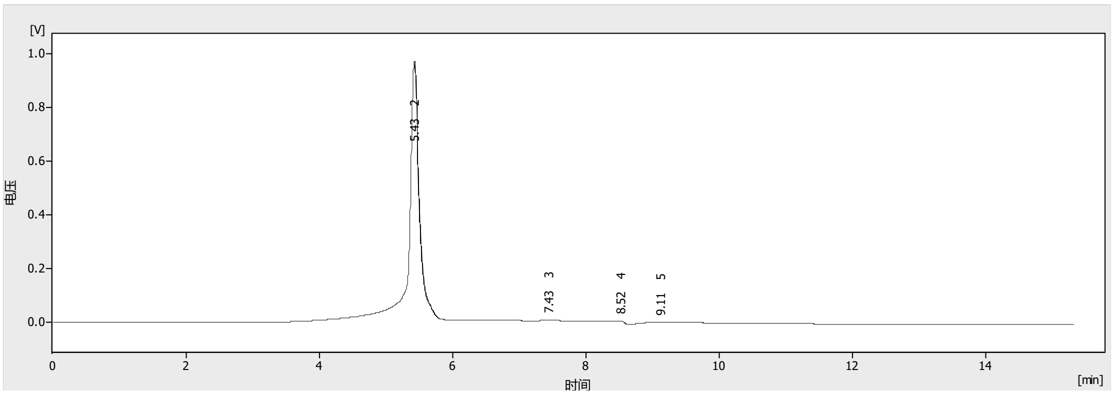

No	Name	Retention/min	Peak area/mV·s	Peak height/mV	Area/%
1	N.A.	0.128	6.835	0.753	0.1
2	N.A.	5.427	11993.129	969.640	96.6
3	N.A.	7.433	88.598	3.745	0.7
4	N.A.	8.517	129.712	11.035	1.0
5	N.A.	9.113	193.446	5.149	1.6
6	Total		12411.720	990.322	100.0

Compound 13a

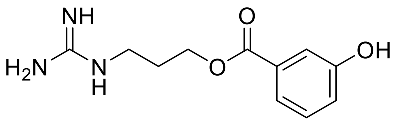

^{1}H-NMR

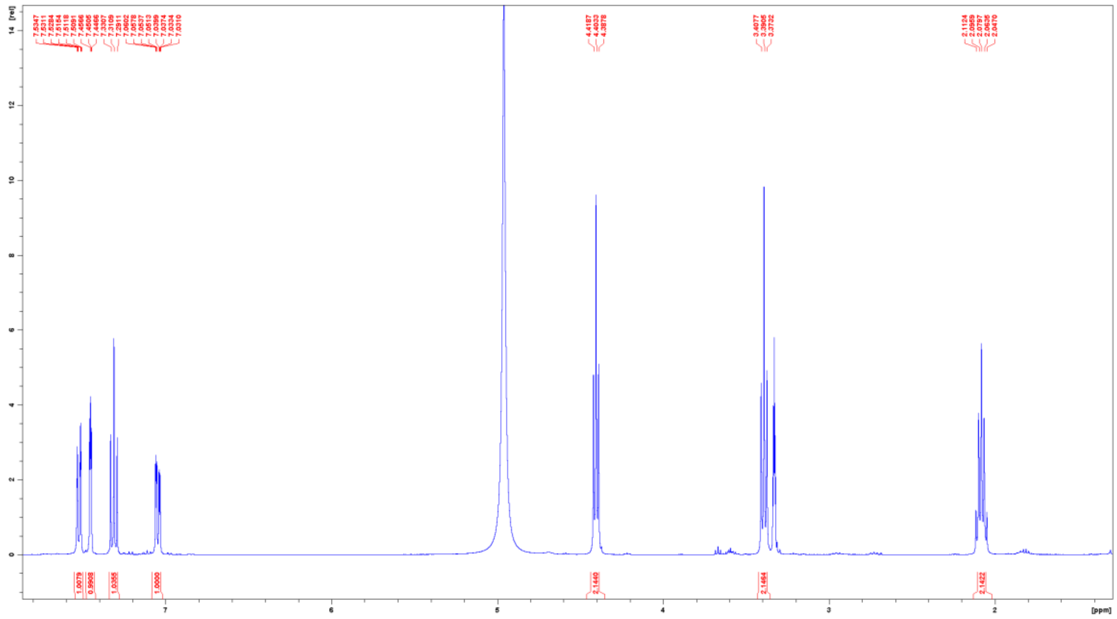

^{13}C-NMR

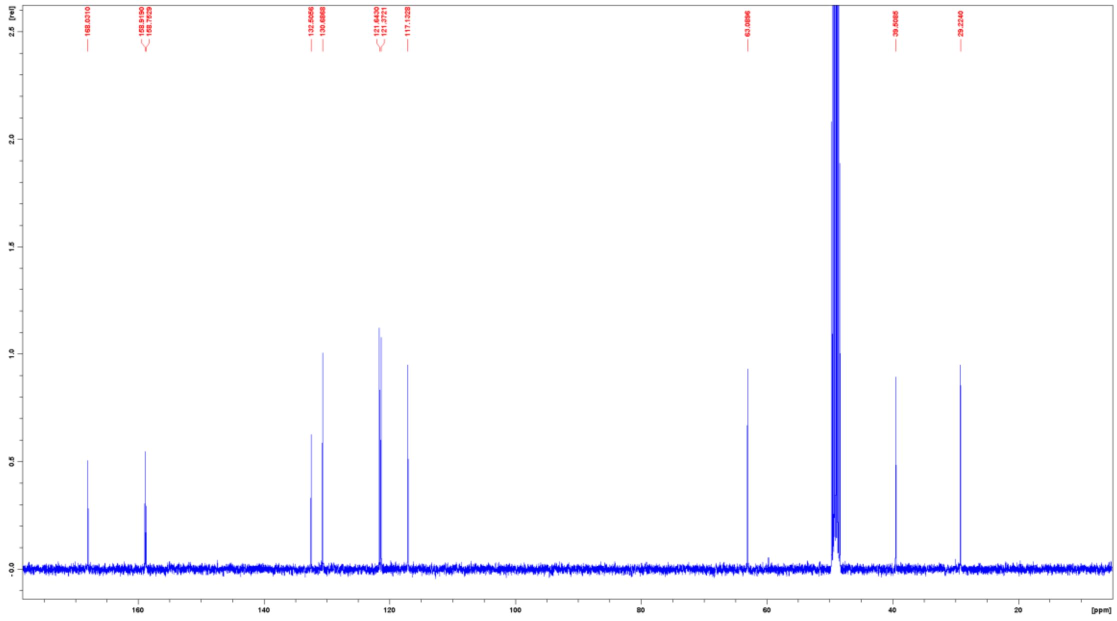

HR- ESI -MS $[M+H]^+$

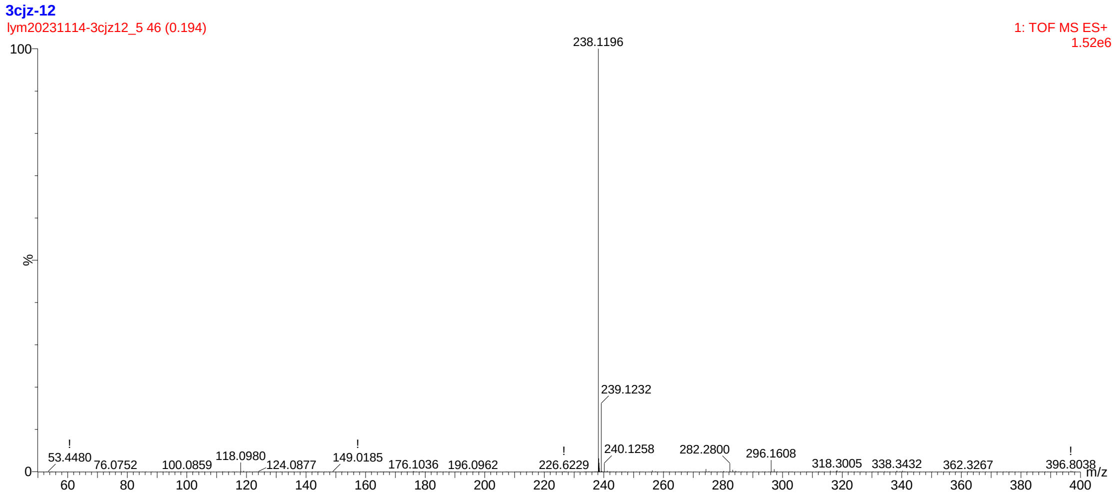

HPLC analysis

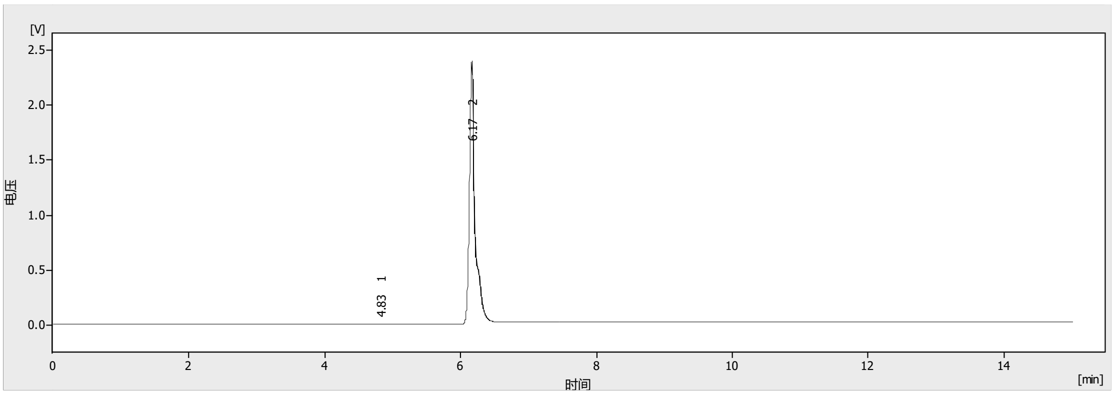

No	Name	Retention/min	Peak area/mV·s	Peak height/mV	Area/%
1	N.A.	4.833	22.516	0.934	0.2
2	N.A.	6.167	14623.947	2401.429	99.8
3	Total		14646.463	2402.363	100.0

Compound 13b

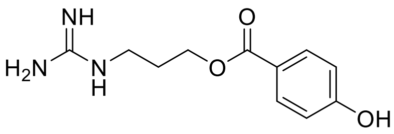

^{1}H-NMR

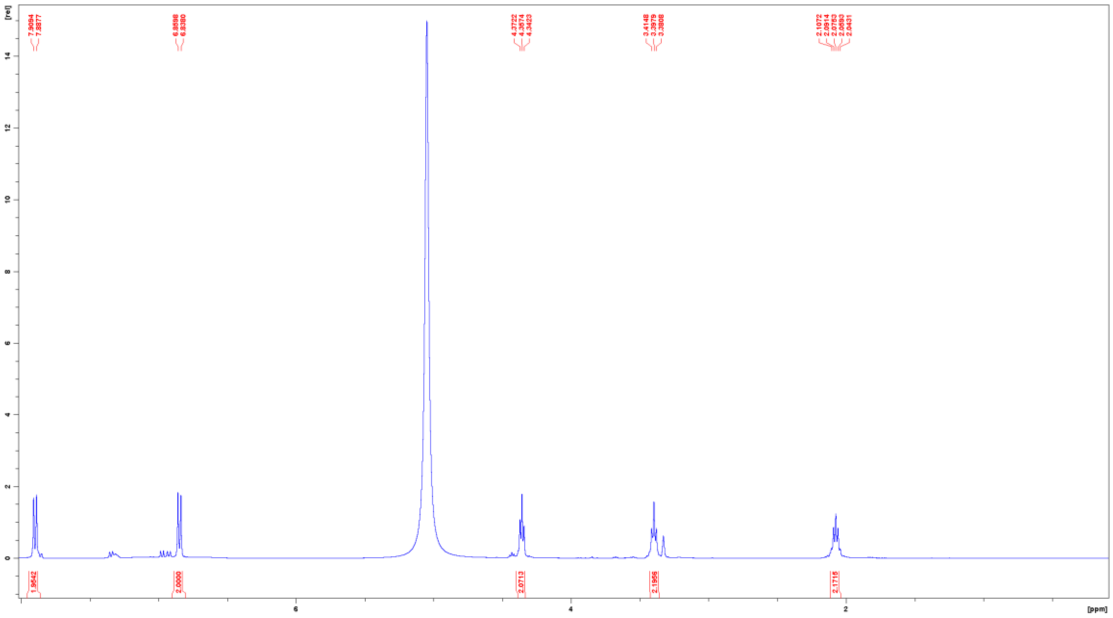

^{13}C-NMR

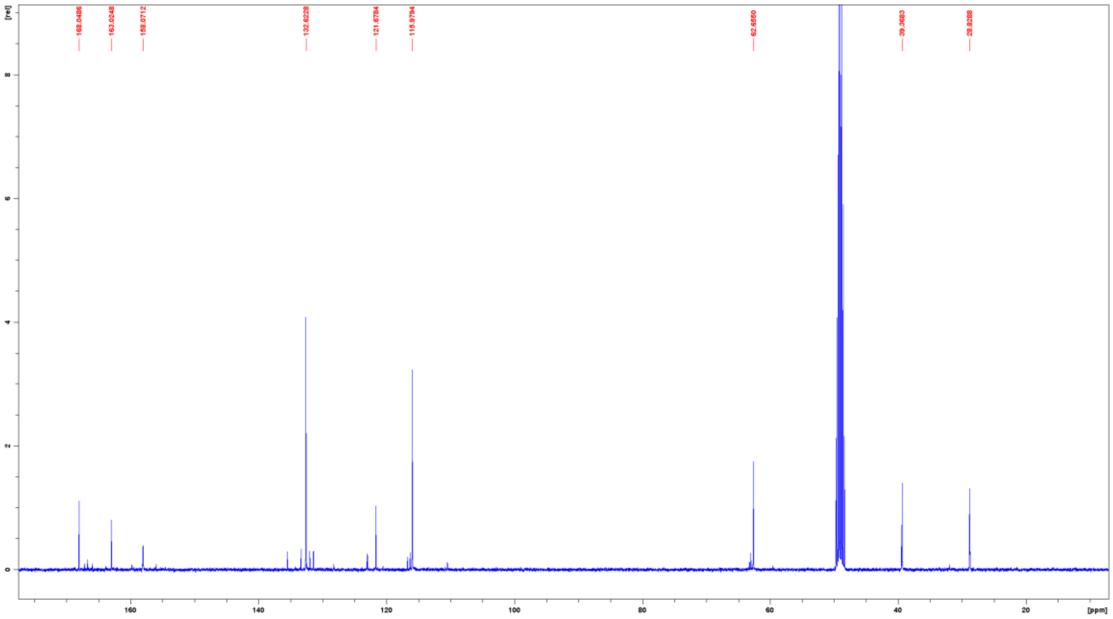

HR- ESI -MS $[M+H]^+$

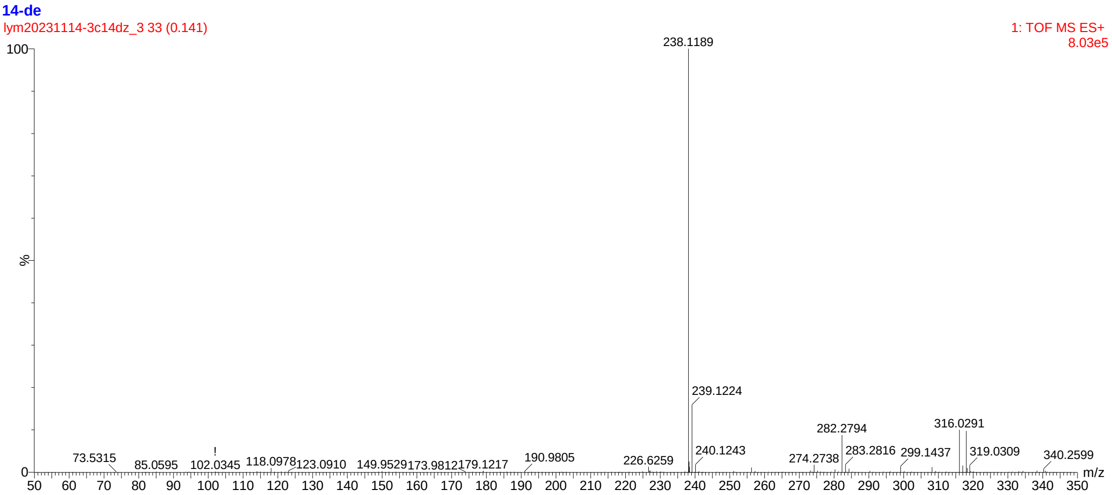

HPLC analysis

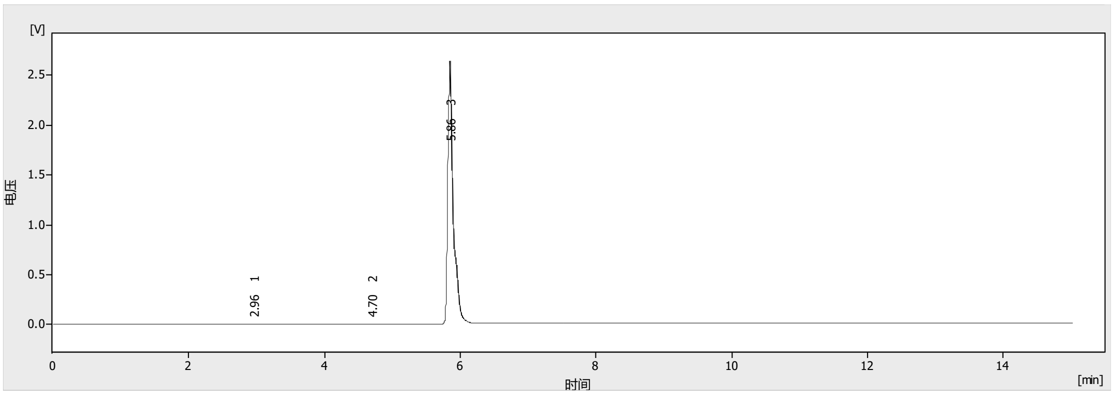

No	Name	Retention/min	Peak area/mV·s	Peak height/mV	Area/%
1	N.A.	2.957	30.110	0.777	0.2
2	N.A.	4.700	5.123	0.407	0.0
3	N.A.	5.862	19500.978	2651.862	99.8
4	Total		19536.211	2653.046	100.0

Compound 13c

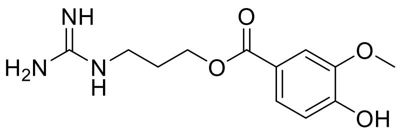

^{1}H-NMR

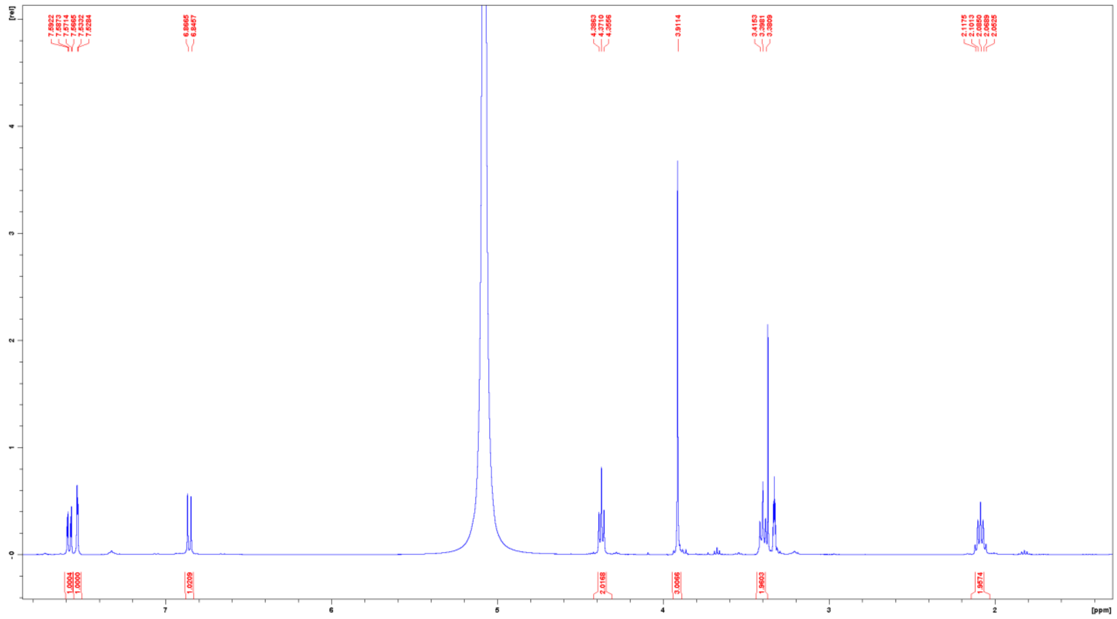

^{13}C-NMR

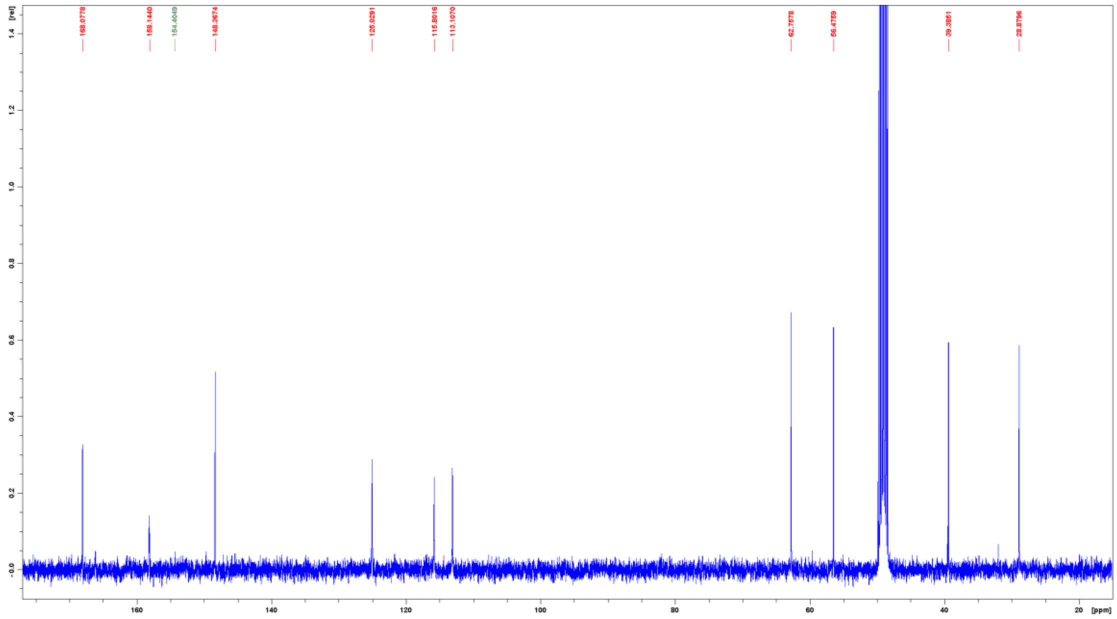

HR- ESI -MS $[M+H]^+$

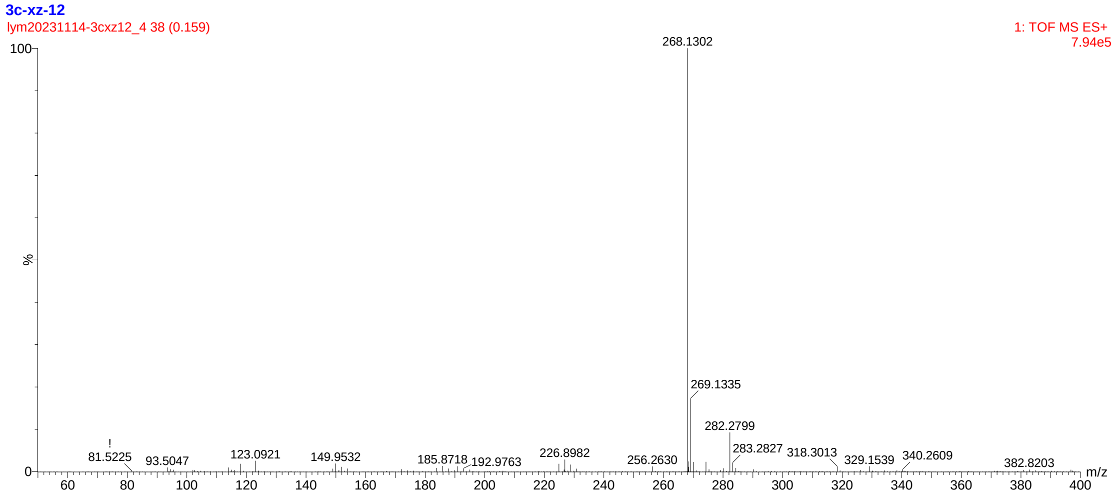

HPLC analysis

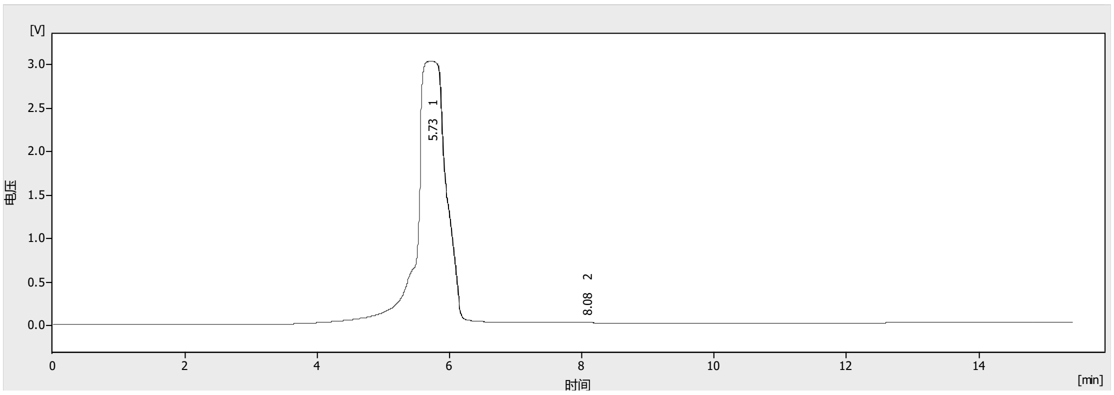

No	Name	Retention/min	Peak area/mV·s	Peak height/mV	Area/%
1	N.A.	5.730	93313.911	3017.107	99.8
2	N.A.	8.082	226.451	7.223	0.2
3	Total		93540.363	3024.331	100.0

Compound 13d

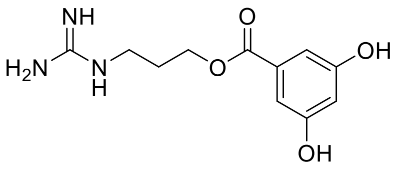

^{1}H-NMR

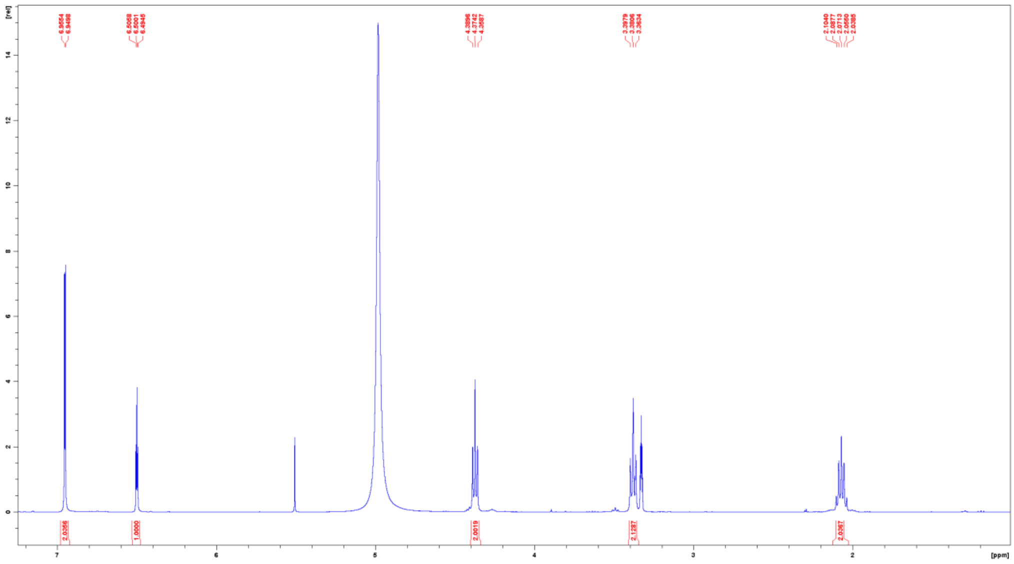

^{13}C-NMR

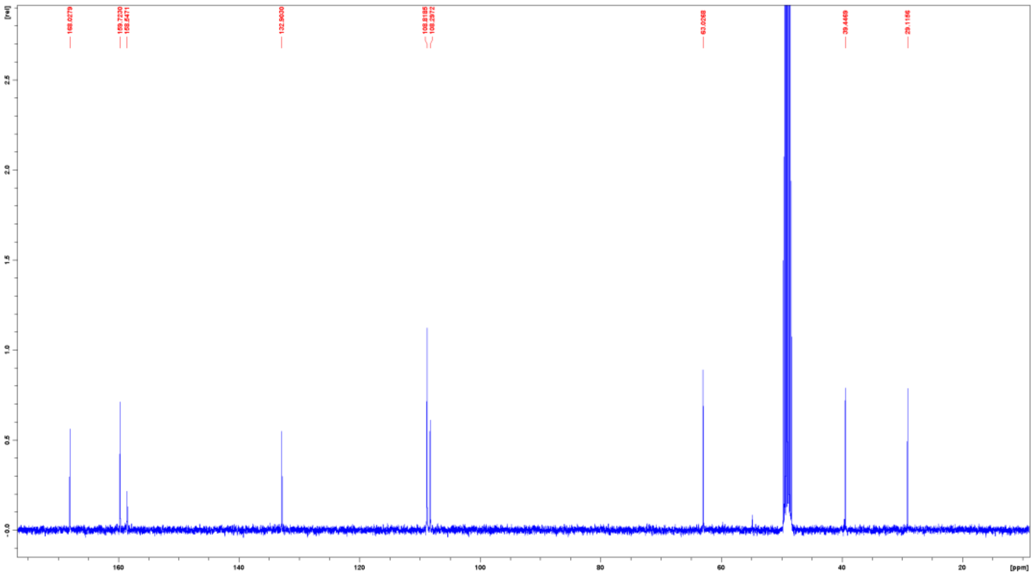

HR- ESI -MS $[M+H]^+$

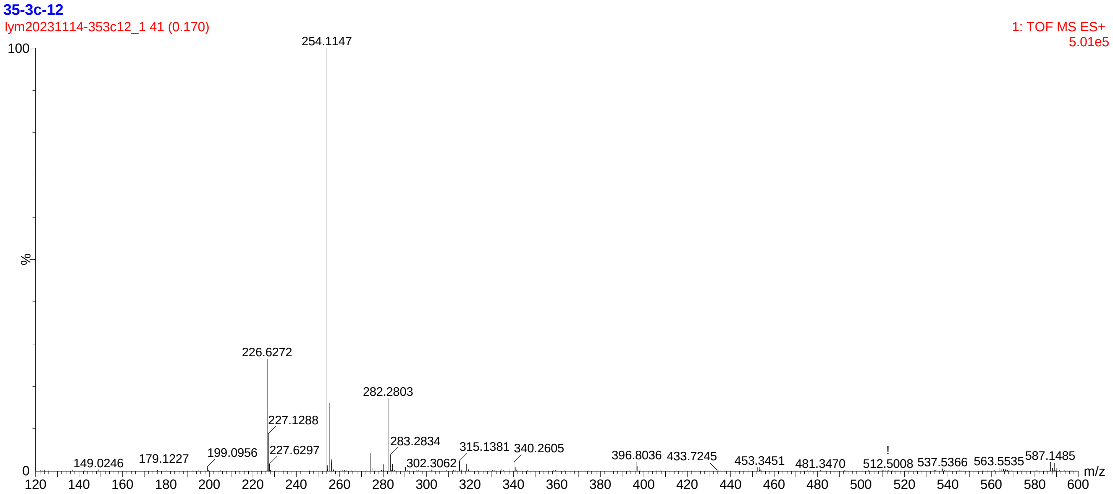

HPLC analysis

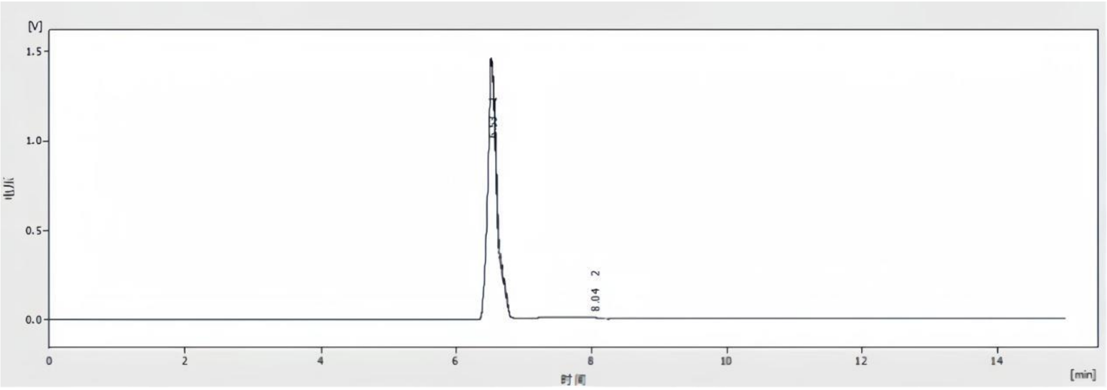

No	Name	Retention/min	Peak area/mV·s	Peak height/mV	Area/%
1	N.A.	6.528	14762.229	1462.821	98.3
2	N.A.	8.045	250.033	10.128	1.7
3	Total		15012.262	1472.948	100.0

Compound 13e

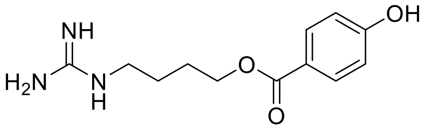

^{1}H-NMR

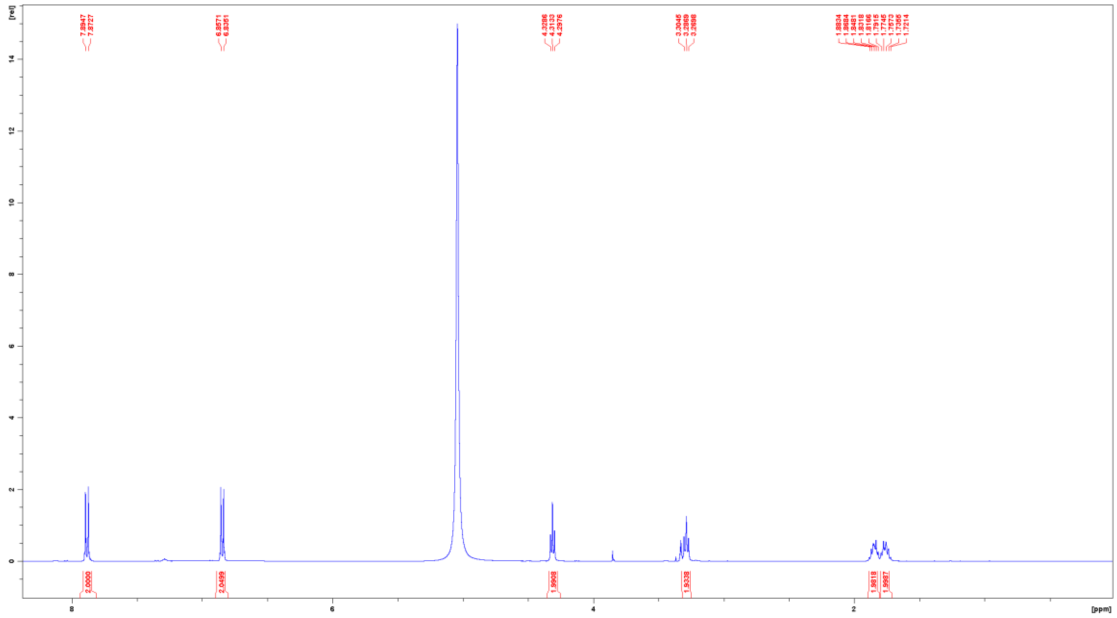

^{13}C-NMR

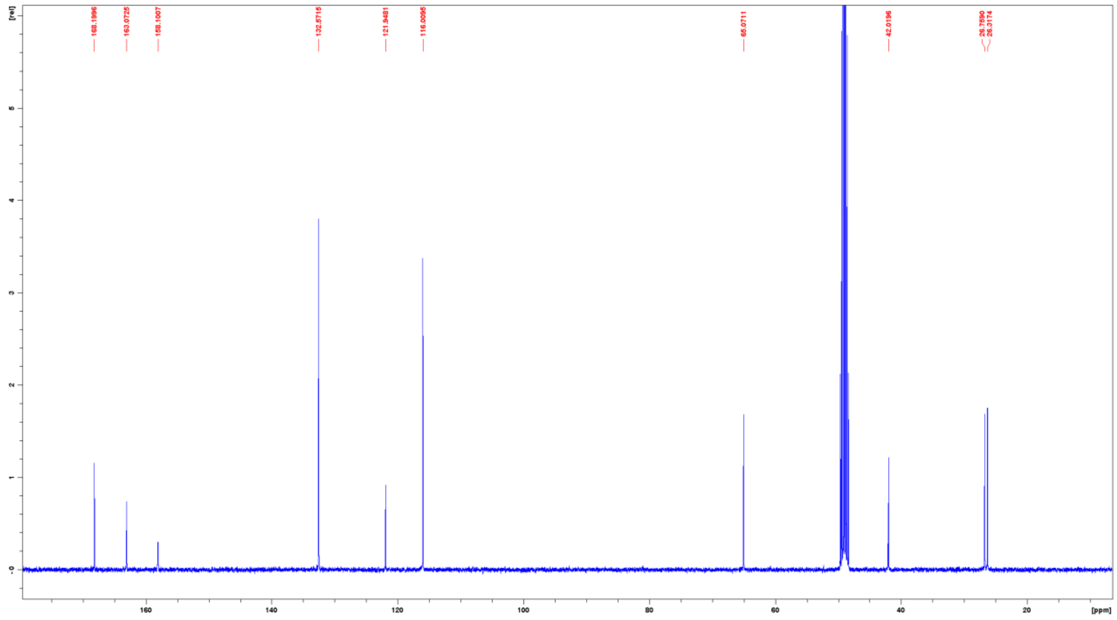

HR- ESI -MS $[M+H]^+$

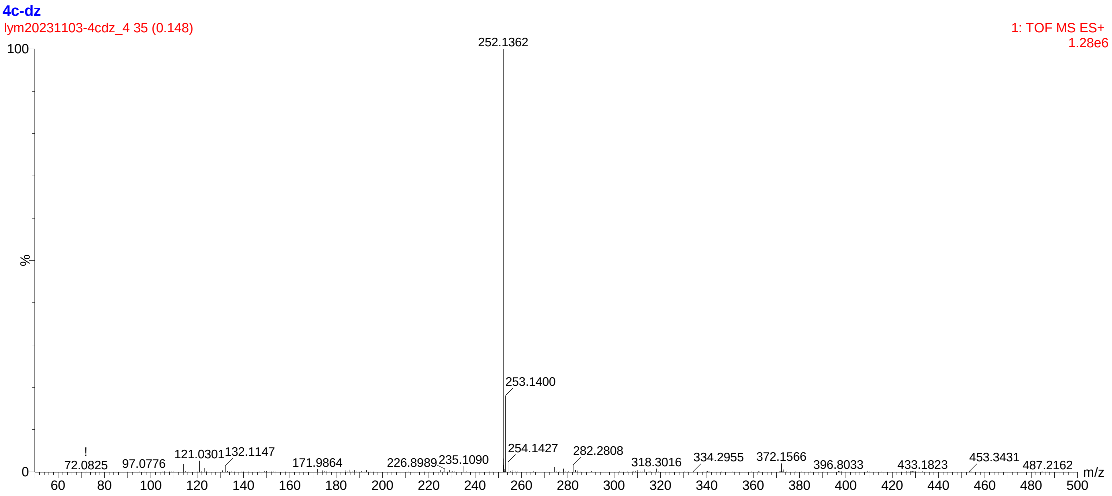

HPLC analysis

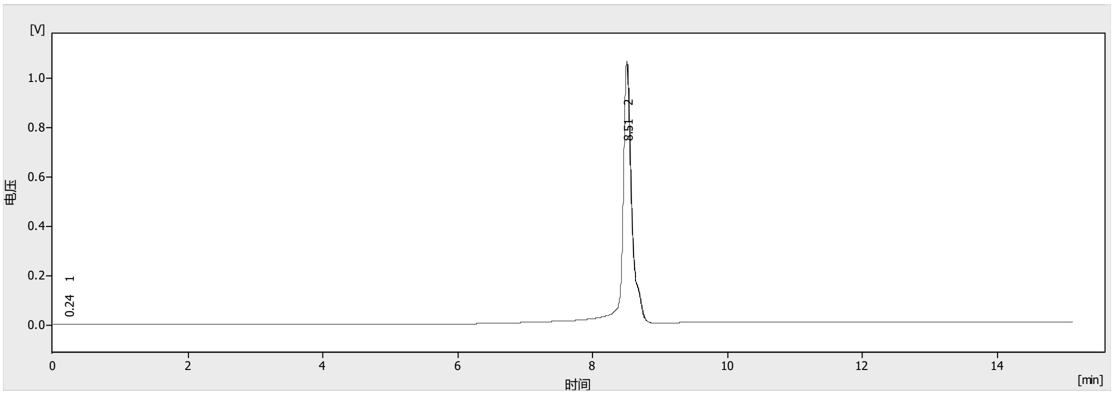

No	Name	Retention/min	Peak area/mV·s	Peak height/mV	Area/%
1	N.A.	0.238	30.389	1.136	0.3
2	N.A.	8.513	10170.239	1059.805	99.7
3	Total		10200.628	1060.941	100.0
